# Ubiquitin-conjugating enzyme UBE2N modulates proteostasis in immunoproteasome-positive acute myeloid leukemia

**DOI:** 10.1172/JCI184665

**Published:** 2025-05-15

**Authors:** Chiharu Ishikawa, Laura Barreyro, Avery M. Sampson, Kathleen M. Hueneman, Kwangmin Choi, Sophia Y. Philbrook, Issac Choi, Lyndsey C. Bolanos, Mark Wunderlich, Andrew G. Volk, Stephanie S. Watowich, Kenneth D. Greis, Daniel T. Starczynowski

**Affiliations:** 1 Division of Experimental Hematology and Cancer Biology, Cincinnati Children’s Hospital Medical Center, Cincinnati, Ohio, USA.; 2 Department of Cancer Biology, University of Cincinnati, Cincinnati, Ohio, USA.; 3 Department of Immunology, MD Anderson Cancer Center, Houston, Texas, USA.; 4 Department of Pediatrics, University of Cincinnati, Cincinnati, Ohio, USA.; 5 University of Cincinnati Cancer Center, Cincinnati, USA.

**Keywords:** Hematology, Oncology, Leukemias, Ubiquitin-proteosome system

## Abstract

Altered protein homeostasis through proteasomal degradation of ubiquitinated proteins is a hallmark of many cancers. Ubiquitination, coordinated by E1, E2, and E3 enzymes, involves up to 40 E2-conjugating enzymes in humans to specify substrates and ubiquitin linkages. In a screen for E2 dependencies in acute myeloid leukemia (AML), ubiquitin conjugating enzyme E2 N (UBE2N) emerged as the top candidate. To investigate UBE2N’s role in AML, we characterized an enzymatically defective mouse model of UBE2N, revealing UBE2N’s requirement in AML without an impact on normal hematopoiesis. Unlike other E2s, which mediate lysine-48 (K48) polyubiquitination and degradation of proteins, UBE2N primarily synthesizes K63-linked chains, stabilizing or altering protein function. Proteomic analyses and a whole-genome CRISPR-activation screen in pharmacologically and genetically UBE2N-inhibited AML cells unveiled a network of UBE2N-regulated proteins, many of which are implicated in cancer. UBE2N inhibition reduced their protein levels, leading to increased K48-linked ubiquitination and degradation through the immunoproteasome and revealing UBE2N activity is enriched in immunoproteasome-positive AML. Furthermore, an interactome screen identified tripartite motif–containing protein 21 (TRIM21) as the E3 ligase partnering with activated UBE2N in AML to modulate UBE2N-dependent proteostasis. In conclusion, UBE2N maintains proteostasis in AML by stabilizing target proteins through K63-linked ubiquitination and prevention of K48 ubiquitin–mediated degradation by the immunoproteasome. Thus, inhibition of UBE2N catalytic function suppresses leukemic cells through selective degradation of critical proteins in immunoproteasome-positive AML.

## Abstract 2

UBE2N enzyme stabilizes key oncoproteins in AML, preventing their degradation through K63-linked ubiquitination. Inhibiting UBE2N selectively degrades these proteins, suppressing leukemia.

## Introduction

**Figure 1:**
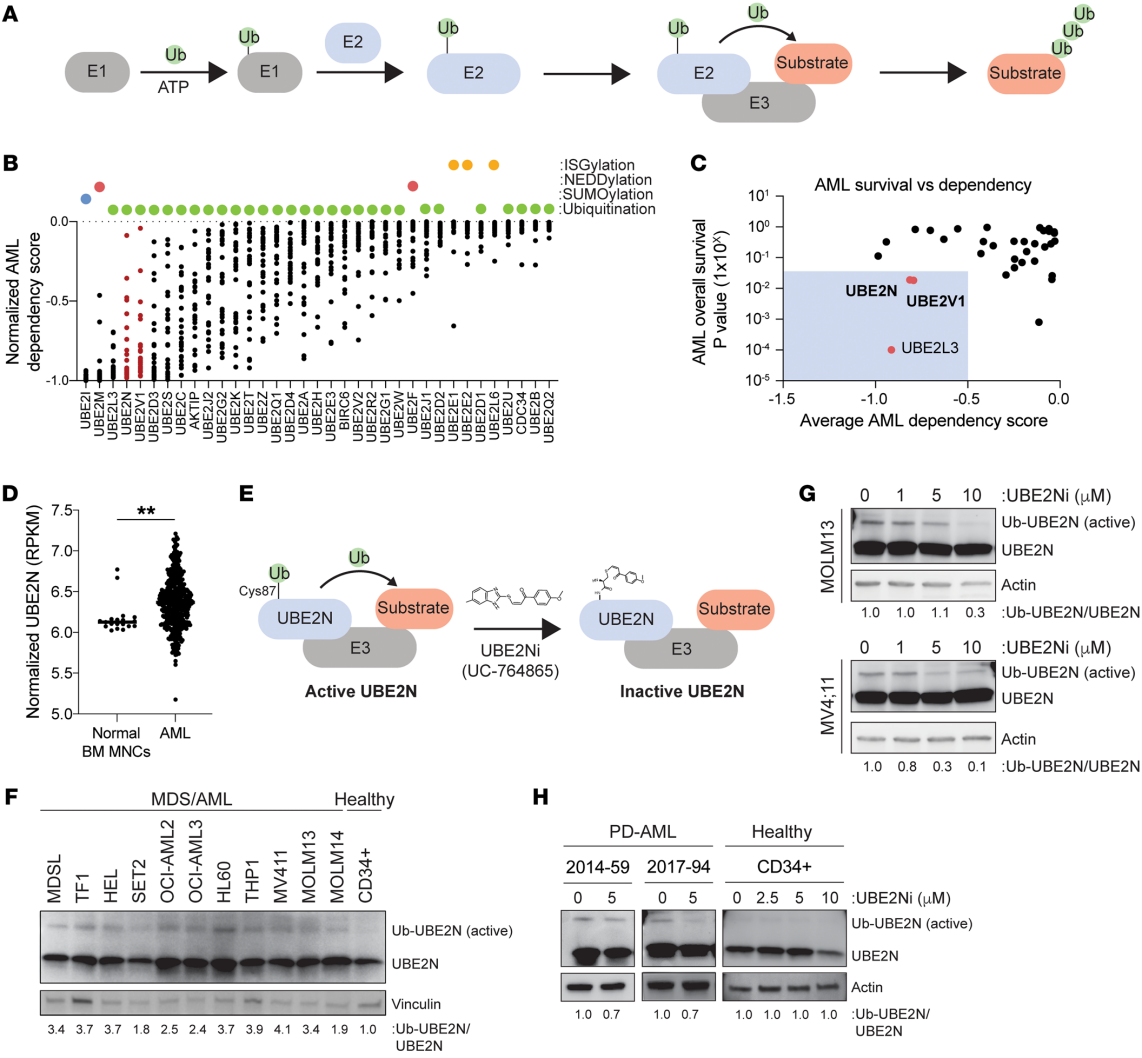
Activated UBE2N is a dependency in human AML. (**A**) Schematic of ubiquitination. E1 activates a ubiquitin molecule and transfers to E2. E2 and E3 ligase transfer ubiquitin to substrates. (**B**) CRISPR/Cas9 screen (DepMap) to identify E2 dependencies in AML cell lines. Green dots represent E2s involved in ubiquitination. Each dot in the graph represents an individual cell line. (**C**) AML dependency score from DepMap for each E2 enzyme versus *P* value of the survival in AML patients. Top 20% and bottom 20% expression levels were used for each E2 (BEAT-AML). (**D**) mRNA expression of *UBE2N* in AML patients and healthy BM mononuclear cells (MNCs) (BEAT-AML). Student’s *t* test (unpaired, 2-tailed) was used to determine significance. Error bars represent the SEM. **P < 0.01. (**E**) UBE2N binding with ubiquitin at cysteine 87 (C87). UBE2Ni (UC-764865) binds C87 and blocks the binding of ubiquitin. (**F**) Activated UBE2N (Ub-UBE2N) and total UBE2N protein expression in AML and healthy CD34^+^ cells. Densitometric values were calculated based on the expression of Ub-UBE2N relative to total UBE2N. (**G**) Immunoblots of UBE2Ni-treated MOLM13 and MV4;11 cells. Densitometric values were calculated based on the expression of Ub-UBE2N relative to total UBE2N. Cells were treated with 1, 5, and 10 μM for 12 hours. (**H**) Immunoblots of UBE2Ni-treated PD-AML and healthy CD34^+^ cells. AML (5 μM) and CD34^+^ cells (2.5, 5, and 10 μM) were treated with UBE2Ni for 12 hours. Densitometric values were calculated based on the expression of Ub-UBE2N relative to total UBE2N.

Acute myeloid leukemia (AML) originates in hematopoietic stem and progenitor cells (HSPCs) following the acquisition of mutations that result in impaired myeloid differentiation and increased self-renewal potential (1, 2). Our understanding of the molecular and genetic factors driving adult AML has increased; however, the 5-year survival rate remains under 20%, with only modest gains over the past 30 years. A combination of chemotherapy, allogeneic stem cell transplantation, and/or emerging targeted therapies has demonstrated promising responses in clinical settings (3–5). However, durable clinical remissions are rarely achieved in nearly all cases, which underscores the urgency for identifying AML dependencies to improve treatment strategies. Protein homeostasis (proteostasis), particularly through ubiquitination, plays a role in the development of many cancers. Ubiquitination is a prevalent and multifaceted posttranslational modification implicated in nearly all aspects of eukaryotic biology (6). The ubiquitination process involves 3 enzymes: the E1-activating enzyme, the E2-conjugating enzyme, and the E3 ligase (Figure 1A). First, the E1-activating enzyme activates the ubiquitin molecule using adenosine triphosphate (ATP). Then, the activated ubiquitin molecule is transferred to the E2-conjugating enzyme. Finally, the E2-conjugating enzyme binds with the E3 ligase, mediating the activated ubiquitin molecule to be covalently attached to a given substrate. Ubiquitin molecules can be added to substrates in various forms, including mono- and polyubiquitination. Ubiquitin can form distinct chain-like configurations by utilizing different lysines on itself. Importantly, the specific ubiquitin linkages have diverse effects on the substrate, leading to various biological processes. For example, K48 and K63 linkages are the most abundant and account for approximately 80% of total linkages in mammalian cells (7). However, K48- and K63-linked ubiquitin chains lead to different fates for substrates. K48-linked ubiquitin chains are the most common linkage in cells, usually making up more than 50% of all ubiquitin linkage chains and direct target proteins to proteasomal degradation (8, 9). In contrast, K63-linked ubiquitin chains mediate scaffolding functions and signal transduction, particularly in immune signaling (10, 11). The type of ubiquitin linkage is primarily determined by the E2-conjugating enzymes (12–14).

Alterations of ubiquitin enzymes are associated with various human malignancies including cancers as a result of dysregulated degradation of proteins or downstream pathways. Therefore, various small molecules have been developed to interfere with ubiquitin processes by inhibiting E1, E2, and E3 ligases, their cofactors, or the proteasome (15–34). Targeting the first step of ubiquitination by blocking E1 function has shown limited clinical benefit due to toxicity, while inhibiting E3 ligases has proven challenging due to the lack of druggable pockets or functional redundancy of E3 ligases. More recently, targeted protein degradation approaches utilizing heterobifunctional molecules or molecular glues effectively induce the degradation of specific proteins by promoting K48-mediated proteasomal degradation (35–37). These strategies have enabled the targeting of challenging-to-drug proteins. However, resistance mechanisms and the drug-like properties of these molecules have restricted their therapeutic potential.

E2 enzymes typically function as intermediaries in the ubiquitin pathway, yet they also play a direct role in tumor development by affecting processes such as DNA repair, cell-cycle regulation, apoptosis, and oncogenic signaling (38–60). In addition, few attempts have been reported to target specific E2-conjugating enzymes, most of which remain at the preclinical stage (38, 61–66). Comprising 40 members, E2 enzymes facilitate ubiquitin-substrate conjugation, thereby regulating the stability and interactions of numerous proteins in a cell-type–dependent manner (39). Therefore, one potential therapeutic strategy for AML involves interfering with specific E2 ubiquitin–conjugating enzymes. While ubiquitination regulates protein homeostasis and function, the functional contributions of E2-dependent processes to leukemic transformation have not been sufficiently explored. To identify E2 dependencies in AML, we utilized publicly available CRISPR/Cas9 screens and identified the ubiquitin conjugating enzyme E2 N–UBE2V1 (UBE2N-UBE2V1) complex among the top targets in AML. UBE2N is a K63-specific ubiquitin E2-conjugating enzyme and its nonenzymatic cofactor, UBE2V1, adds K63-linked polyubiquitin chains along with select E3 ligases to protein substrates. Unlike K48-linked polyubiquitin chains that lead to protein degradation, UBE2N-mediated K63-linked polyubiquitin chains result in the activation of downstream pathways. UBE2N is involved in various cellular processes, including innate immune and inflammatory signaling, DNA damage response, and mitophagy (10, 67–71). Moreover, inhibiting UBE2N has been shown to suppress cancer cell growth in several solid cancers and various types of leukemia and lymphoma (13, 16, 72–76). We recently reported that inhibiting UBE2N with a selective small molecule inhibitor can suppress myelodysplastic syndrome (MDS) and AML cells (77). Despite the significance of UBE2N in AML, the precise mechanistic basis for its dependency in these leukemias has remained unknown. Herein, we demonstrate that the catalytic function of UBE2N is indispensable for maintaining protein homeostasis within oncoprotein networks in AML by preventing immunoproteasome degradation.

## Results

### Activated UBE2N is a dependency in human AML.

To identify E2 dependencies in AML, we analyzed the Cancer Dependency Map (DepMap) CRISPR data sets for all human E2s in 26 diverse AML cell lines (Figure 1B and Supplemental Table 1; supplemental material available online with this article; https://doi.org/10.1172/JCI184665DS1). In parallel, we examined the expression of the E2s in primary AML samples and further narrowed the candidate E2s to ones in which their expression inversely correlated with overall survival (Figure 1C). We focused on E2 genes depleted in the greatest number of AML samples that were not essential across other cell types (78) and that correlated with AML survival outcomes (79) (Supplemental Table 2). Among the E2s, UBE2N emerged as a top hit, as it exhibited a high AML dependency score (score = –0.813) (Figure 1, B and C) and its elevated expression in AML correlated with worse overall survival (*P* = 0.019) (Supplemental Figure 1A). Across human cancers, the dependency of UBE2N in AML is among the highest, but other cancers also show a UBE2N dependency (Supplemental Figure 1B). UBE2N expression is also elevated in a proportion of AML patients compared with normal bone marrow (BM) mononuclear cells (Figure 1D). UBE2N expression is elevated in more monocytic AML subtypes (FAB M3-M6); however, its expression does not correlate with genetic subtypes or risk stratification (Supplemental Figure 1C). Moreover, its cofactor UBE2V1 also ranked highly in the dependency map and outcome correlation for AML, underscoring the catalytic requirement of UBE2N (Figure 1, B and C). UBE2N’s active site includes a cysteine at position 87 (Cys-87), which is critical for binding ubiquitin, facilitating its transfer, and extending K63-linked ubiquitin chains on substrates (65, 80) (Figure 1E). The ubiquitin-conjugating enzyme function of UBE2N can be inferred by the thioester formation between ubiquitin and the active site cysteine (Ub-UBE2N) (65, 80, 81). Conversely, interfering with the thioester bond between ubiquitin and Cys-87, such as with the covalent small molecule inhibitor UC-764865, inhibits UBE2N function (77) (Figure 1E). In AML cell lines, UBE2N is activated (Ub-UBE2N) as indicated by the higher molecular weight protein, but not in normal CD34^+^ cells (Figure 1F). Moreover, activated UBE2N is associated with increased K63-linked ubiquitination in patient-derived AML (PD-AML) samples relative to normal CD34^+^ cells (Supplemental Figure 1E). To confirm that UBE2N is indeed activated, AML cell lines and PD-AML samples treated with the UBE2N inhibitor (UBE2Ni) UC-764865 showed reduction of Ub-UBE2N (Figure 1, G and H). In contrast, the migration of UBE2N protein was unaffected in healthy CD34^+^ cells (Figure 1H). These findings suggest that UBE2N exists in an activated state and is implicated in AML.

### Catalytically inactive UBE2N suppresses AML.

**Figure 2:**
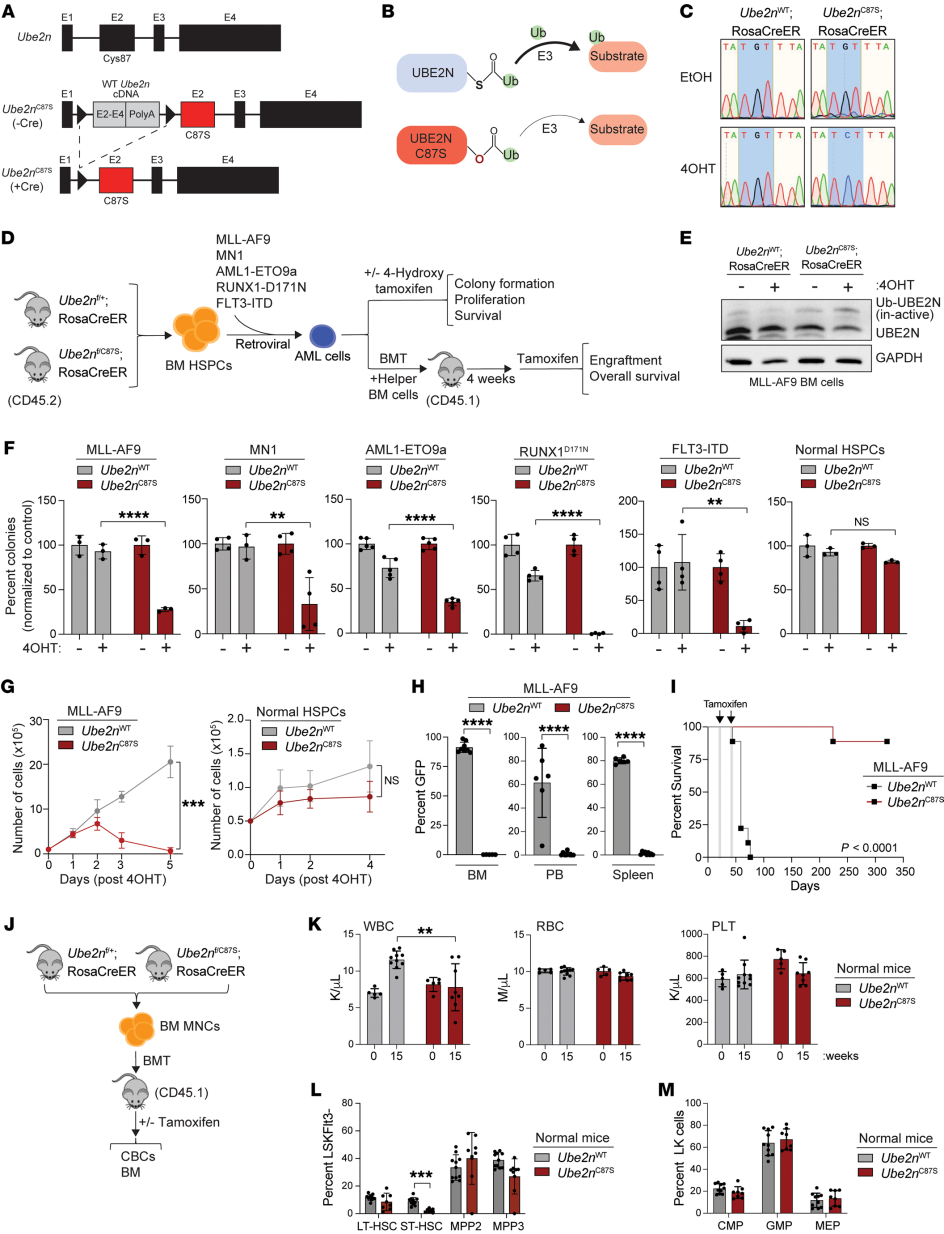
A catalytic inactive mutant of UBE2N suppresses AML. (**A**) UBE2N mouse model to substitute cysteine-87 to serine (C87S). WT UBE2N cDNA cassette is excised by tamoxifen and replaced by mutant (C87S) exon 2. (**B**) UBE2N^C87S^ mutation inhibits the transfer of ubiquitin to its substrates. (**C**) DNA-Seq confirmed recombination from cysteine (TGT) to serine (TCT) in MLL-AF9 cells. (**D**) AML models were developed by retroviral expression of leukemia oncogenes in *Ube2n^WT^* or *Ube2n^C87S^* HSPCs. (**E**) Immunoblots of MLL-AF9 *Ube2n^WT^* and *Ube2n^C87S^* cells. (**F**) Colony formation of normal HSPCs (*n* = 3) or MLL-AF9– (*n* = 3), MN1- (*n* = 4), AML1-ETO9a– (*n* = 5), RUNX1^D171N^- (*n* = 4), and FLT3-ITD–transduced (*n* = 4) *Ube2n^WT^* and *Ube2n^C87S^* cells. (**G**) Cell proliferation of normal HSPCs or MLL-AF9 *Ube2n^WT^* and *Ube2n^C87S^* cells (*n* = 3). (**H**) Flow cytometry analysis of GFP^+^ BM, spleen, and peripheral blood cells. (**I**) Kaplan-Meier survival of *Ube2n^WT^* and *Ube2n^C87S^* MLL-AF9 cells transplanted into irradiated BoyJ mice (*n* = 9 per group). Tamoxifen was injected at weeks 4 and 6. Mantel-Cox test was used to determine significance. (**J**) Total BM transplant to assess hematopoiesis. BM cells from *Ube2n^WT^* (*n* = 10) or *Ube2n^C87S^* mice (*n* = 8) were transplanted to lethally irradiated mice. (**K**) Peripheral blood counts of white blood cells, red blood cells, and platelets (PLT) from *Ube2n^WT^* and *Ube2n^C87S^* mice before and 15 weeks after tamoxifen. (**L** and **M**) HSPC frequency in BM was analyzed by flow cytometry at week 15 after tamoxifen administration. LK, lineage^–^ cKit^+^; LSK, lineage^–^Sca1^+^cKit^+^; CMP, common myeloid progenitor; GMP, granulocyte-monocyte progenitor, MEP, megakaryocyte erythroid progenitor. Two-way ANOVA (**F**, **G**, and **K**–**M**) or Student’s *t* test (unpaired, 2-tailed) (**H**) was used to determine significance. Error bars represent the SEM. *P < 0.05; **P < 0.01; ***P < 0.001; ****P < 0.0001.

Cys-87 of UBE2N is conserved in humans and mice and is critical for transferring ubiquitin to substrates via K63 linkages (Figure 1E). To determine the enzymatic requirement of UBE2N in AML, we generated a mouse model in which Cys-87 is replaced by serine (C87S) to impair its catalytic function (Figure 2A). This mutation leads to reduced — but not entirely lost — enzyme function, effectively modeling the loss of UBE2N catalysis and the impact of UBE2N inhibitors (Figure 2B). Ubiquitin bound to serine through an oxyester bond (Ser-Ub) is thermodynamically more stable than the thioester bond between ubiquitin and cysteine (Cys-Ub) (82). Therefore, Ser-Ub hydrolysis and transfer of ubiquitin from *Ube2n^C87S^* to its substrate is impaired compared with WT *Ube2n* (Figure 2B). The targeted allele was designed to express WT Ube2n protein via codon-optimized *Ube2n* exons 2–4 cDNA (Figure 2A). *Ube2n^C87S^* mice were bred to an inducible recombinase strain (Rosa26CreERT2), leading to expression of the *Ube2n^C87S^* mutant allele following in vivo tamoxifen or in vitro 4-hydroxytamoxifen (4-OHT) treatment. 4-OHT treatment of BM cells confirmed recombination of the WT *Ube2n* exons 2–4 cDNA resulting in expression of the mutant exon (Figure 2C). To evaluate the catalytic function of UBE2N in AML, we modeled AML in lineage-negative (Lin^–^) BM cells from *Ube2n^C87S^*;Rosa26CreERT2 or control mice by retroviral expression of MLL-AF9, MN1, AML1-ETO9a, RUNX1^D171N^, or FLT3-ITD AML oncogenes (Figure 2D). Following flow cytometric isolation of oncogene-expressing AML cells, in vitro 4-OHT treatment resulted in *Ube2n^C87S^* recombination and protein expression, as indicated by the thermodynamically stable version of Ube2n (Ub-Ube2n inactive) (Figure 2E and Supplemental Figure 2, A, and B). To confirm the hypomorphic activity of *Ube2n^C87S^*, cells were stimulated with IL-1β, and the expression of *Ube2n^C87S^* was shown to suppress activation of NF-κB signaling (Supplemental Figure 2C). Expression of *Ube2n^C87S^* resulted in suppression of leukemic progenitor cell function in methylcellulose colony assays (Figure 2F). In addition, expression of *Ube2n^C87S^* resulted in reduced proliferation of AML cells in vitro (Figure 2G and Supplemental Figure 2D). In contrast, *Ube2n^C87S^* expression did not impact WT progenitor cell function, proliferation, or viability (Figure 2, F and G). These findings indicate that UBE2N catalytic function is necessary for murine AML cells.

We next determined the role of UBE2N on AML development in vivo. Lin^–^ BM cells from *Ube2n^C87S^* or WT Rosa26CreERT2 mice (*Ube2n^WT^*) were transduced with MLL-AF9 or MN1 and transplanted into lethally irradiated mice (Figure 2D). Three to four weeks after transplantation, recipient mice were injected with tamoxifen intraperitoneally to induce recombination of *Ube2n^C87S^*. Mice transplanted with *Ube2n^WT^* MLL-AF9 or MN1 AML cells showed signs of leukemia, including myeloid blasts in the BM and peripheral blood (Supplemental Figure 2E). In contrast, mice engrafted with *Ube2n^C87S^* MLL-AF9 or MN1 AML cells had fewer circulating leukemic cells (Figure 2H and Supplemental Figure 2F). Moreover, recipient mice engrafted with *Ube2n^C87S^* AML cells had prolonged survival as compared with mice engrafted with *Ube2n^WT^* AML cells (Figure 2I and Supplemental Figure 2G). To determine whether the catalytic function of UBE2N is essential for normal hematopoiesis, *Ube2n^C87S^* and *Ube2n^WT^* BM cells were transplanted into lethally irradiated WT recipient mice. Following engraftment (4 weeks), recipient mice were injected with tamoxifen intraperitoneally to induce recombination of *Ube2n^C87S^* (Figure 2J). Cre-mediated expression of the *Ube2n^C87S^* mutant allele in Lin^–^ BM cells was confirmed (Supplemental Figure 2H). *Ube2n^C87S^* expression in healthy BM did not affect the overall survival of recipient mice, BM HSPC proportions, or blood counts (Figure 2, K–M). These findings demonstrate that UBE2N’s catalytic activity is not essential for normal adult hematopoiesis, while underscoring its critical role in AML.

### Regulation of proteostasis in AML by UBE2N.

**Figure 3:**
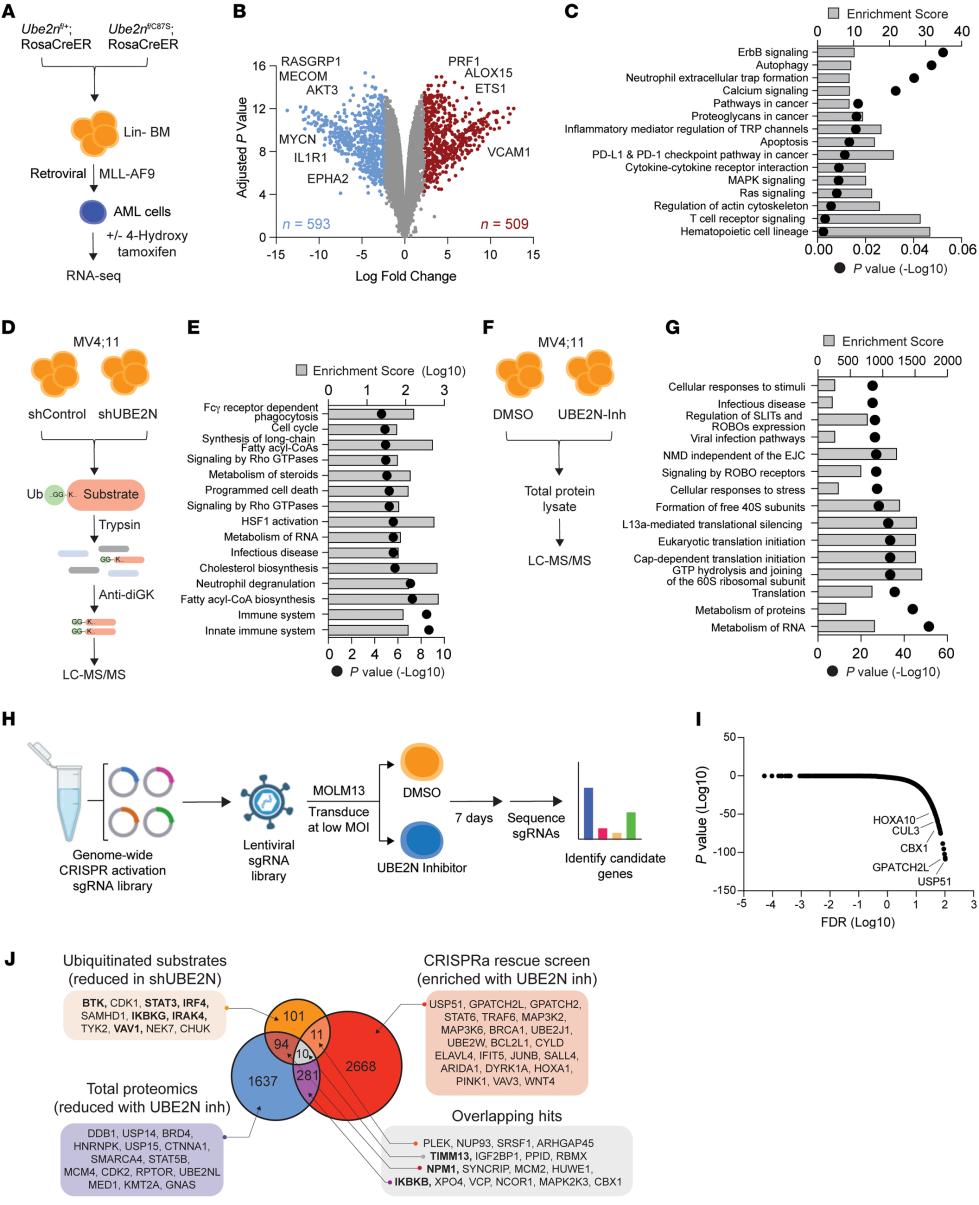
Proteostasis regulation in AML by UBE2N. (**A**) Gene-expression analysis of MLL-AF9–transduced *Ube2n^WT^* and *Ube2n^C87S^* cells. The cells were treated with 4-OHT for 48 hours and RNA was collected for sequencing. (**B**) Volcano plots of differentially expressed genes. (**C**) Pathway enrichment analysis using Kyoto Encyclopedia of Genes and Genomes (KEGG) of the significantly downregulated genes in *Ube2n^C87S^* cells (>2-fold change; *P* < 0.05). The enrichment score and corresponding *P* value is shown. (**D**) Ubiquitin-enriched proteomic of MV4;11 cells transduced with nontargeting shRNA (shControl) or shUBE2N. After selection, these cells were lysed and digested by trypsin, followed by the enrichment of the ubiquitin-bound peptides using K-e-GG magnetic beads and analysis by liquid chromatography–tandem mass spectrometry (LC-MS/MS). (**E**) Pathway enrichment analysis using KEGG of the significantly reduced ubiquitinated substrates in UBE2N-deficient condition. The enrichment score and corresponding *P* value are shown. (**F**) Total proteomics analysis of MV4;11 cells treated with UBE2Ni (UC-65, 5 μM) or vehicle for 24 hours and then lysed and digested by trypsin, followed by LC-MS/MS. (**G**) Pathway enrichment analysis using KEGG of the significantly reduced proteins in UBE2N-inhibited conditions. The enrichment score and corresponding *P* value are shown. (**H**) Overview of the CRISPRa screen in MOLM13 cells expressing dCas9-VP64 and lentiviral sgRNA pooled library. After selection, the cells were treated with DMSO or 2.5 μM UBE2Ni for 7 days, and deep sequencing was performed. (**I**) The FDR and the corresponding *P* value of top hits from CRISPRa screen. (**J**) Venn diagram of the commonly identified hits from UBE2N-dependent substrates from MS and enriched genes from CRISPRa screen.

To uncover the key signaling pathways and gene-expression alterations resulting from suppression of the catalytic activity of UBE2N in AML cells, we performed RNA-Seq on *Ube2n^C87S^* and *Ube2n^WT^* MLL-AF9 AML cells. Expression of *Ube2n^C87S^* resulted in significantly differentially expressed genes as compared with WT MLL-AF9 AML cells (Figure 3A). 593 Genes exhibited a 2-fold decrease in expression, while 509 genes showed a 2-fold increase in expression (Figure 3B and Supplemental Table 3). Pathway analysis of downregulated genes in *Ube2n^C87S^* compared with *Ube2n^WT^* AML cells revealed reduced enrichment of hematopoietic stem cell– and cancer-related pathways (Figure 3C). *Ube2n^C87S^* AML cells showed a reduction in genes associated with Ras, MAPK, apoptosis, and ErbB signaling (Figure 3C). Prior studies have primarily linked UBE2N to the regulation of immune and inflammatory signaling, making its widespread impact on multiple cancer-related pathways unexpected.

Since UBE2N regulates protein ubiquitination, we also performed global quantitative ubiquitin capture proteomics in MV4;11 AML cells expressing nontargeting shRNA (shControl) or shUBE2N (Figure 3D and Supplemental Table 10). Ubiquitinated peptides immunoprecipitated from nontargeting shRNA (shControl) or shUBE2N-MV4;11 cells were analyzed by mass spectrometry. Since a proteasome inhibitor was not used to enrich for K48-linked ubiquitinated proteins, most ubiquitinated proteins captured would represent K63 ubiquitin linkages. The proteomic analysis identified 277 peptides corresponding to 217 proteins that exhibited reduced ubiquitination following knockdown of UBE2N (fold change >|0.5|; *P* < 0.05) (Supplemental Table 4). As expected, UBE2N regulates the ubiquitination of proteins involved in immune signaling as well as other pathways, such as cholesterol biosynthesis, HSF1 activation, and fatty acyl-CoA biosynthesis, that have not been previously implicated in UBE2N-dependent function (Figure 3E and Supplemental Figure 3A). Differential K63-linked ubiquitination can also impact protein stability and expression; we therefore investigated the change in total protein levels upon suppression of UBE2N. For this, we performed global quantitative proteomics in MV4;11 AML cells treated with the UBE2Ni followed by mass spectrometry (Figure 3F). The proteomic analysis identified 1,159 proteins that showed reduced expression following inhibition of UBE2N (fold change >|0.5|; *P* < 0.05) (Supplemental Table 5). Of these targets, 104 were also implicated in UBE2N-dependent ubiquitination, representing approximately 50% of the ubiquitinated substrates (Figure 3G and Supplemental Table 4). Therefore, UBE2N regulates the ubiquitination and protein expression of a network of cancer-related targets in AML.

To understand which cellular processes lead to the loss of AML cells when UBE2N is inhibited, we performed a whole-genome CRISPR activation (CRISPRa) screen to identify genes that could rescue the growth potential of UBE2N-inhibited AML cells. MOLM13 cells expressing dCas9-VP64 were transduced with a CRISPRa library consisting of sgRNA activating 18,000 coding isoforms (Figure 3H). Transduced cells were treated with the UBE2Ni for 7 days and the sgRNA libraries were deep sequenced. MAGeCK (version 0.5.9, https://sourceforge.net/p/mageck/wiki/Home/) was then performed to identify candidate genes enriched in UBE2Ni-treated cells relative to control cells (Figure 3H and Supplemental Table 6). The top enriched genes (top 20%) in UBE2Ni-treated cells included USP51 (rank #1, *P* = 2.03 × 10^-9^) and GPATCH2L (rank #2, *P* = 1.73 × 10^-8^). USP51 is a deubiquitinase implicated in the progression and metastasis of several cancers (83–87). In contrast, not much is reported on GPATCH2L. Several top-ranking candidate genes enriched in UBE2Ni-treated cells mediate cancer cell properties, such as CBX1 (88), CUL3 (89, 90), and HOXA10 (91–95) (Figure 3I). This analysis revealed that UBE2N inhibition can be rescued by reactivation of cancer-related signaling programs. To identify the mechanistic basis for UBE2N dependency in AML, we prioritized the top targets from the ubiquitin-enrichment screen (Figure 3D), the total proteome analysis (Figure 3F), and the CRISPRa screen (Figure 3H). The objective of this approach is to identify targets of UBE2N, which would exhibit decreased ubiquitination and expression upon UBE2N suppression (either through knockdown or inhibition), but could restore leukemic cell function when overexpressed in UBE2Ni-treated AML cells (Figure 3J). Based on this strategy, we focused on candidates that appeared in at least 2 of the orthogonal screens or are putative ubiquitinated substrates of UBE2N, which included several key regulators of cancer cells, such as TIMM13 (96, 97), STAT3 (98–104), BTK (105–107), IRAK4 (108–113), NPM1 (114–116), IKKβ (117, 118), and SYNCRIP (119, 120) (Figure 3J). These findings suggest that UBE2N positively regulates critical oncoprotein networks via ubiquitination in AML.

### UBE2N maintains oncoproteins by preventing immunoproteasome-mediated degradation.

**Figure 4:**
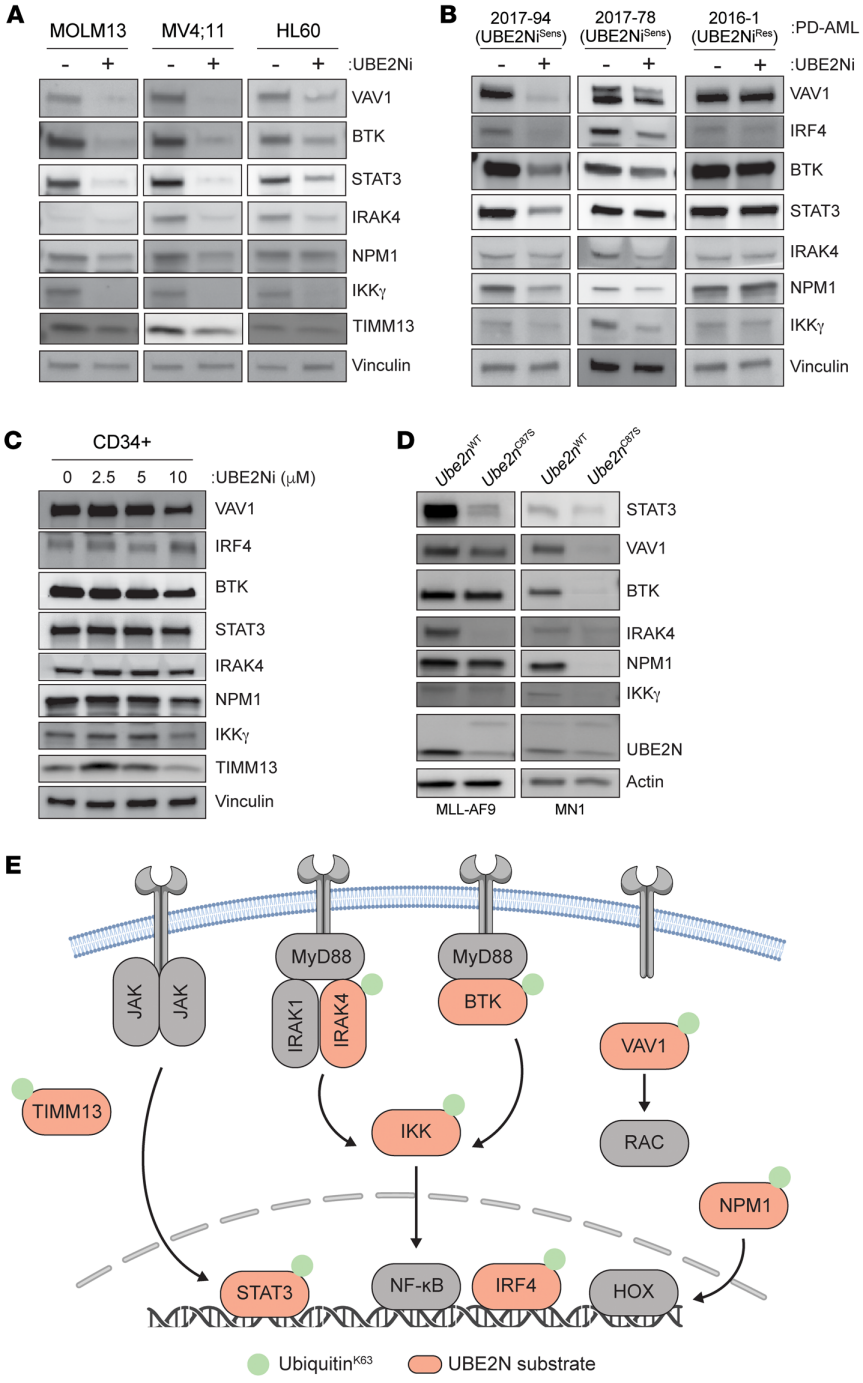
UBE2N maintains oncoprotein expression by preventing immunoproteasome-mediated degradation. (**A**) Immunoblots of identified targets in MOLM13, MV;411, and HL60 cells treated with UBE2Ni (UC-65, UBE2Ni) for 24 hours (5 μM). (**B**) UBE2Ni-sensitive (2017-94 and 2017-78) or -resistant AML PD-AML cells (2016-1) were treated with UBE2Ni (5 μM) for 12 hours and immunoblotted for the indicated proteins. (**C**) Healthy CD34^+^ cells were treated with UBE2Ni (0, 2.5, 5, 10 μM) for 12 hours and immunoblotted for the indicated proteins. (**D**) *Ube2n^WT^* or *Ube2n^C87^* MLL-AF9 and MN1 cells were treated with 4-OHT for 24 hours and immunoblotted for the indicated proteins. (**E**) Schematic figure of signaling pathways of UBE2N substrates.

UBE2N-mediated K63-linked ubiquitination of substrates does not lead to protein degradation, but rather influences protein activation, localization, recycling, or stability. To increase protein stability, K63-linked ubiquitination can interfere with K48-linked ubiquitin on the same protein substrate (121). Thus, K63 linkages can modulate the recognition and processing of K48-linked ubiquitinated proteins by the proteasome, thereby stabilizing the protein. Therefore, we first wanted to determine whether UBE2N inhibition impacts the expression of the identified target proteins. UBE2Ni-treated AML cells resulted in a reduction in nearly all the target proteins (Figure 4A), which was observed within 6 hours of UBE2Ni treatment (Supplemental Figure 3B). We next examined target protein stability in PD-AML samples that are sensitive (UBE2Ni^Sens^) or resistant (UBE2Ni^Res^) to UBE2Ni treatment. The UBE2Ni^Sens^ AML samples treated with the UBE2Ni exhibited a reduction in the target proteins (Figure 4B). In contrast, the expression of these proteins in UBE2Ni^Res^ AML samples or healthy CD34^+^ cells was not affected by the UBE2Ni (Figure 4, B and C). Moreover, the target protein levels were reduced in *Ube2n^C87S^* MLL-AF9 and MN1 mouse AML cells as compared with WT AML cells (Figure 4D). Importantly, the reduction in target protein levels was not due to changes in mRNA expression (Supplemental Figure 3, C–E). These data suggest that UBE2N maintains the expression of cancer-associated proteins, such as STAT3, IRAK4, IKKγ, BTK, VAV1, and NPM1 in AML (Figure 4E).

**Figure 5:**
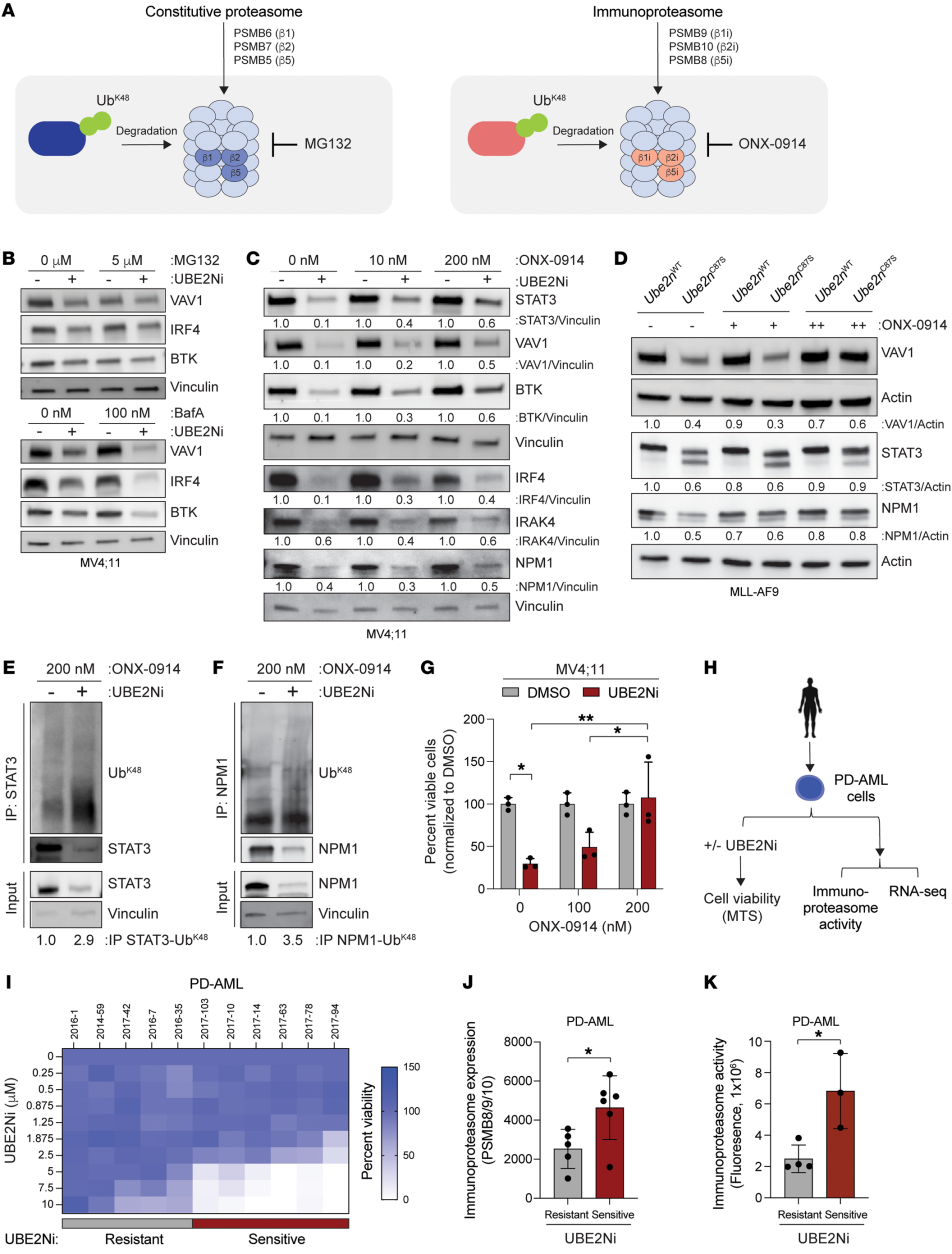
UBE2N dependency is predominant in immunoproteasome-positive AML. (**A**) Proposed model. (**B**) Immunoblots of MV4;11 cells cotreated with DMSO or UBE2Ni (5 μM) and the constitutive proteasome inhibitor MG132 (5 μM, 6 hours) or the lysosomal inhibitor bafilomycin A (100 nM, 20 hours). (**C**) Immunoblots of MV4;11 cells treated with DMSO or UBE2Ni (5 μM) and the immunoproteasome inhibitor ONX-0914 (10 nM or 200 nM) for 24 hours. (**D**) Immunoblots of *Ube2n^WT^* or *Ube2n^C87S^* MLL-AF9 cells cotreated with 4-OHT (0.5 μM) and ONX-0914 (10, 100, or 200 nM) for 24 hours. (**E**) Coimmunoprecipitation in MV4;11 cells treated with DMSO or UBE2Ni (5 μM) and ONX-0914 (200 nM) for 24 hours. NPM1 and STAT3 were immunoprecipitated and immunoblotted for K48-linked polyubiquitination. Densitometric values were calculated based on the expression of K48-ubiquitinated NPM1 (left) or STAT3 (right) relative to immunoprecipitated NPM1 or STAT3. (**F**) Cell viability of MV4;11 cells (n = 3 per group) 48 hours after treatment with UBE2Ni (2.5 μM) and ONX-0914 (100 nM and 200 nM). Two-way ANOVA was used to determine significance. (**G**) Overview of experiments using PD-AML. PD-AML cells were (a) treated with UBE2Ni and MTS assay was conducted or (b) analyzed by RNA-Seq. (**H**) Heatmap of percentage of viability of UBE2Ni-treated PD-AML cells determined by MTS assay. PD-AMLs were classified as UBE2Ni resistant or sensitive. (**I**) The mRNA expression levels of immunoproteasome genes in UBE2Ni-sensitive (*n* = 5) and -resistant (*n* = 6) PD-AML. (**J**) Immunoproteasome activity was measured in UBE2Ni-sensitive and -resistant PD-AML. Student’s *t* test (unpaired, 2-tailed) was used to determine significance (**I** and **J**). Error bars represent the SEM. *P < 0.05; **P < 0.01.

Since UBE2N activity correlates with increased protein expression of specific targets in AML, we hypothesized that UBE2N-mediated K63-linked ubiquitin chains prevent degradation of its targets by K48-linked ubiquitination and subsequent proteasomal degradation (Figure 5A). Degradation of K48-linked ubiquitinated proteins primarily occurs via the 26S proteasome. However, constitutive proteasome inhibitors (MG132) failed to restore expression of UBE2N-regulated proteins (Figure 5B). To investigate whether these proteins were being degraded by lysosomes, we also employed a lysosomal inhibitor (bafilomycin A1). However, bafilomycin A1 did not restore expression of the UBE2N-regulated proteins (Figure 5B). In hematopoietic cells and under inflammatory or oxidative stress, an alternative form of the proteasome, the immunoproteasome, is utilized to degrade proteins (Figure 5A). The immunoproteasome consists of the 20S core and 19S regulatory subunits similar to the constitutive proteasome. However, the 3 subunits of the 20S core (PSMB6/β1, PSM7/β2, and PSMB5/β5) are substituted in the immunoproteasome with PSMB9/β1i, PSMB10/β2i, and PSMB8/β5i (122–124). Previous studies have demonstrated that AML cells express the immunoproteasome to prevent accumulation of protein aggregates and damaged proteins (122, 125–128). Therefore, we next investigated whether UBE2N stabilizes the target proteins by preventing immunoproteasome degradation. The immunoproteasome inhibitor ONX-0914 (129) restored the expression of the target proteins, including BTK, IRAK4, IRF4, NPM1, VAV1, and STAT3, in UBE2Ni-treated AML cells (Figure 5C). Additionally, ONX-0914 restored the expression of the oncoproteins in *Ube2n^C87S^* MLL-AF9 AML cells (Figure 5D). Upon UBE2N inhibition, K48-mediated ubiquitination of NPM1 and STAT3 was increased in AML cells treated with ONX-0914, suggesting that loss of UBE2N results in increased K48-linked ubiquitination and degradation of oncoproteins via the immunoproteasome (Figure 5E). Moreover, immunoproteasome inhibition with ONX-0914 rescued the functional defect of AML cells treated with UBE2Ni (Figure 5F). These data reveal that UBE2N protects target proteins from degradation to maintain leukemic cell function via the immunoproteasome in AML.

### UBE2N dependency is predominant in immunoproteasome-positive AML.

The immunoproteasome genes are overexpressed in more than 50% of AML patients and are correlated with adverse risk and myelomonocytic subtypes (125) (Supplemental Figure 4, A and B). To determine whether UBE2N activity is preferentially required in AML cells with elevated immunoproteasome expression, we examined the correlation between UBE2Ni sensitivity and the expression of immunoproteasome subunit genes (Figure 5G and Supplemental Table 7). RNA-Seq was performed on PD-AML samples with diverse genetics (*n* = 11), followed by in vitro treatment with increasing concentrations of the UBE2Ni for cell-viability analysis (Figure 5G). Immunoproteasome activity using a fluorometric assay was assessed in parallel on a subset of samples. Among these, 6 AML samples exhibited sensitivity to UBE2Ni treatment, showing more than 50% cell death at 5 μM, while 5 samples displayed resistance to treatment (Figure 5H). Notably, the AML samples sensitive to UBE2Ni treatment showed an increase in immunoproteasome gene expression (*P* = 0.03, Figure 5I) and activity (*P* = 0.019, Figure 5J) compared with the resistant samples. Furthermore, we utilized a UBE2N-dependency gene signature that correlated with the response of PD-AMLs to increasing concentrations of the UBE2Ni. This allowed us to correlate immunoproteasome gene expression with UBE2N activity in an independent cohort of primary AML samples from BEAT-AML (http://vizome.org/aml2/) (79). The UBE2N-dependency signature was most significantly enriched in AML subtypes (M4 and M5) with the highest expression of immunoproteasome genes (Supplemental Figure 4C). These findings suggest that UBE2N activity is preferentially required in AML subtypes that have developed a dependency on the immunoproteasome.

### UBE2N utilizes TRIM21 for immunoproteasome-dependent degradation of oncoproteins.

**Figure 6:**
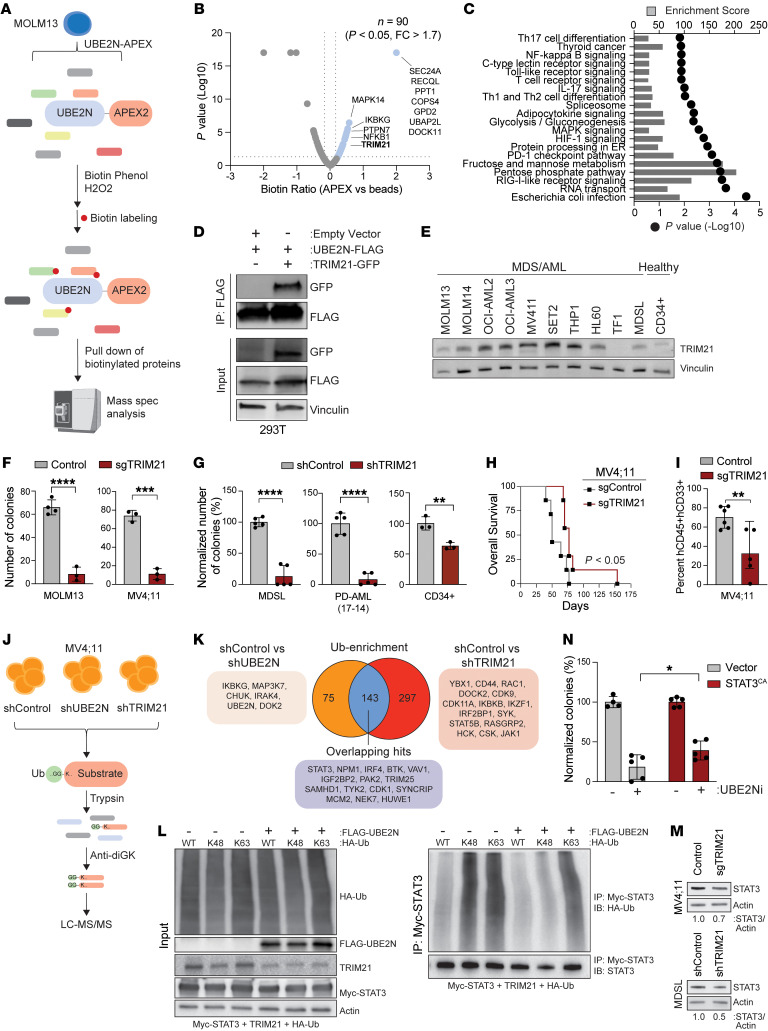
UBE2N utilizes TRIM21 for immunoproteasome-dependent degradation of oncoproteins. (**A**) Proximity assay using doxycycline-inducible UBE2N-V5-APEX2. Cells were treated with biotin phenol to activate APEX2 for biotin labeling and quenched by hydrogen peroxide. Biotinylated proteins were pulled down and analyzed by LC-MS/MS. (**B**) Enriched proximal proteins compared with control (DOX-untreated sample) (1.7 > fold change, *P* < 0.05). (**C**) KEGG pathway enrichment of the hits. The enrichment score and *P* value are shown. (**D**) Coimmunoprecipitation in HEK293T cells expressing empty vector, FLAG-UBE2N, or GFP-TRIM21. FLAG was immunoprecipitated and GFP was immunoblotted. (**E**) Immunoblots in AML and healthy CD34^+^ cells. (**F**) Colony formation of TRIM21-deleted cells (*n* = 3 per group). (**G**) Colony formation assay of TRIM21-deficient MDSL, PD-AML (patient ID 17-14) cells (*n* = 5 per group), and healthy CD34^+^ cells (*n* = 3 per group). (**H**) Kaplan-Meier survival analysis of xenografted mice (*n* = 7 per group). Mantel-Cox test was used to determine significance. (**I**) Engraftment of hCD45^+^hCD33^+^ cells in BM at time of death. (**J**) Ubiquitin enrichment of cells expressing shControl, shUBE2N, or shTRIM21. After selection, protein lysates were digested by trypsin, followed by enrichment of ubiquitinated peptides using K-e-GG magnetic beads and analysis by LC-MS/MS. (**K**) Venn diagram of significantly reduced substrates in UBE2N- and TRIM21-deficient cells from the ubiquitin-enriched analysis. (**L**) Coimmunoprecipitation in HEK293T cells expressing FLAG-UBE2N, TRIM21, myc-STAT3 and HA-Ub (WT, K48-specific, or K63-specific). Myc was immunoprecipitated and HA-ubiquitin was immunoblotted. (**M**) Immunoblots of MV4;11 and MDSL cells. (**N**) Colony formation of MV4;11 cells (n = 5) expressing empty vector or active STAT3 (STAT3^CA^) and treated with UBE2Ni (5 μM). Two-way ANOVA was used to determine significance. Student’s *t* test (unpaired, 2-tailed) was used to determine significance (**F**, **G**, and **I**). Error bars represent the SEM. *P < 0.05; **P < 0.01; ***P < 0.001.

Although we demonstrate that UBE2N is essential to protect target proteins from degradation via the immunoproteasome in AML, the relevant E3 ligases in this process remain unknown. To identify the UBE2N-dependent E3 ligases in AML, we performed proximity labeling of proteins with biotin using APEX2 followed by mass spectrometry (130) (Figure 6A and Supplemental Figure 5). We identified 90 proteins proximal to UBE2N (*P* < 0.05) (Figure 6B and Supplemental Table 8). Several proteins associated with canonical UBE2N signaling were identified, such as IKBKG/IKKγ, NFKB1, and MAPK14 (Figure 6B). To identify UBE2N-signaling networks in AML, we performed an ontology pathway analysis on the list of proteins associated with UBE2N. Proximal UBE2N proteins include effectors of RIG-I, TLR, NF-κB, TCR, MAPK, and HIF-1 signaling (Figure 6C). We next focused on UBE2N proximal proteins that exhibit E3 ligase function. Tripartite motif–containing protein 21 (TRIM21) emerged as the top candidate (Figure 6B). TRIM21 is a RING finger E3 ubiquitin ligase that is implicated in a variety of cancer mechanisms (131–135). Coimmunoprecipitations confirmed the interaction of UBE2N and TRIM21 in HEK293T cells (Figure 6D). Moreover, TRIM21 RNA and protein expression are increased in AML compared with healthy CD34^+^ cells (Figure 6E and Supplemental Figure 6A). Although TRIM21 expression in AML ranks high compared with other human cancers, its dependency score in DepMap across human cancers is relatively low when compared with UBE2N (Supplemental Figure 6, B and C) To investigate whether TRIM21 is required for AML cells, we targeted TRIM21 using RNAi or CRISPR/Cas9 approaches in human AML cell lines and patient-derived samples (Supplemental Figure 6D and Supplemental Table 10). In all TRIM21-deficient AML samples, we observed a reduction (>75% reduction) in the number of leukemic colonies compared with the control cells (Figure 6, F and G). In contrast, knockdown of TRIM21 in normal CD34^+^ cells only had a modest effect (~25% reduction) on colony formation (Figure 6G). Additionally, we conducted a xenograft using isogenic TRIM21^WT^ or TRIM21^KO^ MV4;11 AML cells in immunocompromised mice. Deletion of TRIM21 in MV4;11 cells resulted in a reduction in leukemic cell engraftment in the BM and an extension of overall survival (Figure 6, H and I). We next investigated whether TRIM21 is a critical E3 ligase in UBE2N-dependent AML. TRIM21 overexpression in AML cells deficient for UBE2N or treated with the UBE2Ni rescued the leukemic cell colony–forming defect (Supplemental Figure 6, E–G). These findings suggest that TRIM21 activity is required for UBE2N-dependent AML cells and that its loss phenocopies inhibition of UBE2N.

To delineate the target proteins of the UBE2N/TRIM21 axis in AML, we conducted quantitative ubiquitin capture proteomics (Figure 6J). Ubiquitinated peptides from shControl or shTRIM21 MV4;11 cells were identified and analyzed by mass spectrometry. The proteomic analysis identified 657 peptides corresponding to 445 proteins that exhibited reduced ubiquitination following knockdown of TRIM21 (fold change >|0.5|; *P* < 0.05) (Supplemental Table 9). We then compared the ubiquitination substrates reduced upon knockdown of TRIM21 to the substrates reduced upon UBE2N knockdown (Figure 6J). Among substrates ubiquitinated by both TRIM21 and UBE2N, we identified STAT3, NPM1, IRF4, BTK, and VAV1 (Figure 6K). We focused on the regulation of STAT3 by UBE2N/TRIM21, as STAT3 has been implicated in AML and leukemic stem cells (98–104). Thus, we hypothesized that UBE2N/TRIM21 stabilizes STAT3 by adding K63-linked ubiquitin chains and preventing K48 polyubiquitination and immunoproteasome degradation. To test this, we coexpressed UBE2N and TRIM21 and measured K48- and K63-linked ubiquitination of STAT3. In the absence of UBE2N, STAT3 undergoes both K48- and K63-linked ubiquitination (Figure 6L). However, expression of UBE2N resulted in reduction of K48-linked STAT3 ubiquitination, thereby increasing the ratio of K63-linked versus K48-linked ubiquitination of STAT3 (Figure 6L). This is consistent with the reduced expression of STAT3 protein and increased K48-linked ubiquitination in UBE2N-inhibited AML cells (see Figure 5, C–E). Moreover, deletion or knockdown of TRIM21 in leukemic cells resulted in 30%–50% reduced protein levels of STAT3 (Figure 6M). Lastly, we determined whether the reexpression of STAT3 can restore leukemic cell function following UBE2N inhibition (Supplemental Figure 6H). Overexpression of an active STAT3 partially rescued the leukemic colony formation in AML cells treated with the UBE2Ni (Figure 6N). These data show that UBE2N utilizes the E3 ubiquitin ligase TRIM21 for immunoproteasome-dependent degradation of oncoproteins, such as STAT3, in AML.

## Discussion

We uncovered a critical function of UBE2N in AML through the modulation of oncoprotein proteostasis. Unlike other E2 enzymes that facilitate the degradation of proteins via K48-linked ubiquitination, UBE2N generates K63-linked polyubiquitin chains. This can prevent K48-linked ubiquitination, thereby leading to the stabilization of proteins critical for leukemic cells. Inhibiting UBE2N reduced target protein levels, such as STAT3, BTK, NPM1, and IRAK4, leading to K48-linked ubiquitination and their degradation. UBE2N is involved in various processes, including innate immune and inflammatory signaling, DNA damage response, and mitophagy (10, 67–69). However, we revealed UBE2N regulates oncoproteins that are susceptible to immunoproteasome degradation in AML. Cancer cells exhibit adaptability to disruptions in protein homeostasis, employing mechanisms such as altered protein translational rates, increased proteolytic activity, improved protein repair functions, and/or increased protein recycling (136–140). In normal hematopoiesis, distinct levels of proteostasis regulation are observed between hematopoietic stem cells (HSCs) and progenitor cells (141, 142). Within HSCs, there is evidence of lower rates of protein synthesis, even when these cells are not in a quiescent state. Despite having lower proteasome activity compared with progenitor cells, HSCs exhibit fewer unfolded or misfolded proteins. Yet when HSCs sense the accumulation of misfolded proteins, there is an increase in c-Myc, which could lead to proliferation and self-renewal (141, 142). These adaptive responses are evident in AML as well, where unfolded protein responses (UPR) are elevated, promoting cell proliferation and metabolic functions (139, 143). Additionally, elevated ROS in leukemic cells further induce the UPR. Dysregulation or mutations within the ubiquitin proteolysis system also play a role in cancer by impacting the stability of tumor suppressors or oncoproteins (15, 16, 144). For example, it was recently reported that the resistance mechanism of KRAS inhibitors is due to dysregulated proteostasis, leading to the stabilization of the UPR-regulating protein IRE1a (137). Therapeutic strategies targeting the ubiquitin proteasome system, such as bortezomib, have shown promise in some hematologic malignancies (145). Combined inhibition of autophagy and proteasome activity has demonstrated efficacy in disrupting protein homeostasis and reducing cell viability in AML cells, highlighting the therapeutic potential of targeting protein homeostasis pathways.

Our study revealed TRIM21 as an E3 ligase partnering with UBE2N to regulate proteostasis in AML. TRIM21 interacts with multiple E2 conjugating enzymes, including UBE2N/UBE2V2, UBE2W, UBE2E1, and CDC34, to mediate various types of ubiquitination (e.g., monoubiquitination, K48-, and K63-linked polyubiquitin chains) (146–149). TRIM21 is essential in antiviral immunity, acting by sensing antibody-coated viruses that have evaded extracellular neutralization and breached the cell membrane (132). Upon engagement of antibody, TRIM21 is monoubiquitinated by UBE2W, which leads to ubiquitin chain extension via K63 linkages via UBE2N/UBE2V2 (150). TRIM21 is also implicated in cancer. Multiple proteins involved in cancer metabolism, immunity, and inflammation-associated tumorigenesis are ubiquitination substrates of TRIM21 (135). For example, TRIM21 is involved in degradation of cyclin-dependent kinase 2 (CDK2) and ATG5 in AML and multiple myeloma cells (151, 152). TRIM21 is implicated in both cancer progression and suppression, primarily through K48-linked proteasomal degradation. For instance, it degrades phosphorylated p27 to promote cell-cycle progression and regulates p53 stability (153, 154), supporting tumor progression. Conversely, it suppresses tumorigenesis by degrading mutant p53, HIF-1α, and acetylated FASN (84, 155–157). TRIM21 also mediates K63-linked ubiquitination in cancer cells (134, 158). While TRIM21 has UBE2N-independent roles, our findings suggest the UBE2N/TRIM21 axis plays a critical role in leukemic function, including stabilization of STAT3 by UBE2N/TRIM21 in AML. STAT3, a well-studied gene supporting AML cell proliferation, preventing apoptosis, and sustaining leukemic stem cells, is a target of extensive research as a therapeutic target (98–104). Although we provide a link between UBE2N and STAT3 in AML, both UBE2N and STAT3 are known to cooperatively regulate normal immune and hematopoietic cell function. STAT3 transcriptional activity is required to repress UBE2N expression, thereby influencing the levels of UBE2N in hematopoietic cells to restrain inflammatory signaling (159, 160). Nevertheless, understanding the intricate regulation of STAT3 by various factors, including UBE2N, provides insights into potential therapeutic strategies for AML.

The immunoproteasome is a cell- and context-dependent alternative to the constitutive proteasome, acting by mediating the degradation of proteins with unique amino acid sequences. The immunoproteasome is primarily expressed in hematopoietic cells, in particular antigen-presenting cells, and induced upon oxidative stress and proinflammatory cytokine stimulation (122, 161). The immunoproteasome yields unique peptides, thus allowing for optimal MHC class I antigen presentation during immune responses (124, 162). Thus, immunoproteasome expression in solid tumors can increase antigen presentation and immunogenicity (163). Immunoproteasome genes can be either upregulated or downregulated in various cancers and contribute to both pro- and antitumorigenesis. In our study, as well as in studies conducted by other groups, immunoproteasome genes are found to be elevated in AML (125, 128). Notably, the assembly of immunoproteasomes occurs 4 times faster than that of constitutive proteasomes (164, 165), highlighting a potential dependency of AML cells on immunoproteasome activity. In AML, particularly in the M5 subtype or with KMT2A rearrangements, immunoproteasomes are important for alleviating proteolytic stress and regulating critical pathways (125). In these studies, the genetic and pharmacologic inactivation of the immunoproteasome via PSMB8 resulted in impaired proliferation of murine and human leukemic cells, while normal hematopoietic cells remained unaffected (128). Given the increase in immunoproteasome activity and dependency in AML and the resulting broader degradation of critical proteins, our data suggest that coactivation of UBE2N is required in these leukemias to regulate proteostasis of oncogenic pathways by stabilizing critical proteins. Given the critical role of UBE2N in regulating protein turnover and ubiquitin signaling, uncovering specific deubiquitinating enzymes involved in this pathway could provide valuable insights into how UBE2N-dependent processes contribute to immunoproteasome-positive AML pathogenesis. In summary, UBE2N maintains proteostasis in AML by stabilizing multiple target proteins and preventing their degradation through the immunoproteasome. Moreover, inhibiting UBE2N’s catalytic activity suppressed leukemic stem and progenitor cell functions by destabilizing these critical proteins in AML, which represents a potential therapeutic strategy for immunoproteasome-dependent cancers.

## Methods

### Sex as a biological variable.

Our study used both female and male mice. For further information, see Supplemental Methods.

### Data availability.

The RNA-Seq data have been deposited in the NCBI’s Gene Expression Omnibus database (GEO GSE286041). Publicly available RNA-Seq data were downloaded from The Cancer Genome Atlas (cbioportal.org) (GEO GSE68833) and BEAT-AML (http://www.vizome.org/aml/). Cell lines and mouse models used in these studies are publicly available through commercial sources or may be made available from the authors upon written request and material transfer agreement approval. Plasmids and antibodies used in the study are included in Supplemental Tables 10–13.Values for all data points in graphs are reported in the Supporting Data Values file.

### Statistics.

Statistical analyses were performed as indicated in the figure legends and in the Supplemental Methods section.

### Study approval.

All work was approved by the Institutional Review Board (ID # 2008-0021) and Institutional Animal Care and Use Committee at Cincinnati Children’s Hospital (IACUC 2019-0072, 2022-0054). For further information, see Supplemental Methods.

## Author contributions

CI, LB, and AMS performed experiments, analyzed and interpreted data, and wrote the manuscript. KMH, LB, MW, KDG, LCB, SYP, and IC performed experiments and analyzed and interpreted data. KC performed bioinformatics analyses. AV and SW provided input and reagents and interpreted data. DTS conceived and directed the study, analyzed and interpreted data, and wrote and/or edited the manuscript. All authors approved the final version of the manuscript.

## Supplementary Material

Supplemental data

Unedited blot and gel images

Supplemental tables 1-13

Supporting data values

## Version 1 05/15/2025

Electronic publication

## References

[1] Pollyea DA, Jordan CT (2017). Therapeutic targeting of acute myeloid leukemia stem cells. Blood.

[2] Arnone M (2020). Acute myeloid leukemia stem cells: the challenges of phenotypic heterogeneity. Cancers (Basel).

[3] Lowenberg B (2009). High-dose daunorubicin in older patients with acute myeloid leukemia. N Engl J Med.

[4] Fernandez HF (2009). Anthracycline dose intensification in acute myeloid leukemia. N Engl J Med.

[5] Kantarjian H (2021). Acute myeloid leukemia: current progress and future directions. Blood Cancer J.

[6] Swatek KN, Komander D (2016). Ubiquitin modifications. Cell Res.

[7] Kaiser SE (2011). Protein standard absolute quantification (PSAQ) method for the measurement of cellular ubiquitin pools. Nat Methods.

[8] Komander D, Rape M (2012). The ubiquitin code. Annu Rev Biochem.

[9] Grice GL, Nathan JA (2016). The recognition of ubiquitinated proteins by the proteasome. Cell Mol Life Sci.

[10] Hodge CD (2016). Ubc13: the Lys63 ubiquitin chain building machine. Oncotarget.

[11] Madiraju C (2022). K63 ubiquitination in immune signaling. Trends Immunol.

[12] Liu W (2020). The ubiquitin conjugating enzyme: an important ubiquitin transfer platform in ubiquitin-proteasome system. Int J Mol Sci.

[13] Stewart MD (2016). E2 enzymes: more than just middle men. Cell Res.

[14] Ye Y, Rape M (2009). Building ubiquitin chains: E2 enzymes at work. Nat Rev Mol Cell Biol.

[15] Morrow JK (2015). Targeting ubiquitination for cancer therapies. Future Med Chem.

[16] Deng L (2020). The role of ubiquitination in tumorigenesis and targeted drug discovery. Signal Transduct Target Ther.

[17] Hyer ML (2018). A small-molecule inhibitor of the ubiquitin activating enzyme for cancer treatment. Nat Med.

[18] Yang Y (2007). Inhibitors of ubiquitin-activating enzyme (E1), a new class of potential cancer therapeutics. Cancer Res.

[19] Adams J, Kauffman M (2004). Development of the proteasome inhibitor Velcade (Bortezomib). Cancer Invest.

[20] Vij R (2012). An open-label, single-arm, phase 2 study of single-agent carfilzomib in patients with relapsed and/or refractory multiple myeloma who have been previously treated with bortezomib. Br J Haematol.

[21] Vij R (2012). An open-label, single-arm, phase 2 (PX-171-004) study of single-agent carfilzomib in bortezomib-naive patients with relapsed and/or refractory multiple myeloma. Blood.

[22] Potts BC (2011). Marizomib, a proteasome inhibitor for all seasons: preclinical profile and a framework for clinical trials. Curr Cancer Drug Targets.

[23] Kumar SK (2014). Safety and tolerability of ixazomib, an oral proteasome inhibitor, in combination with lenalidomide and dexamethasone in patients with previously untreated multiple myeloma: an open-label phase 1/2 study. Lancet Oncol.

[24] Piva R (2008). CEP-18770: A novel, orally active proteasome inhibitor with a tumor-selective pharmacologic profile competitive with bortezomib. Blood.

[25] Ito T (2010). Identification of a primary target of thalidomide teratogenicity. Science.

[26] Kim Y, Schmidt-Wolf IG (2015). Lenalidomide in multiple myeloma. Expert Rev Anticancer Ther.

[27] Lacy MQ, Rajkumar SV (2010). Pomalidomide: a new IMiD with remarkable activity in both multiple myeloma and myelofibrosis. Am J Hematol.

[28] Vassilev LT (2007). MDM2 inhibitors for cancer therapy. Trends Mol Med.

[29] Vassilev LT (2004). In vivo activation of the p53 pathway by small-molecule antagonists of MDM2. Science.

[30] Ungermannova D (2013). High-throughput screening AlphaScreen assay for identification of small-molecule inhibitors of ubiquitin E3 ligase SCFSkp2-Cks1. J Biomol Screen.

[31] Kapuria V (2010). Deubiquitinase inhibition by small-molecule WP1130 triggers aggresome formation and tumor cell apoptosis. Cancer Res.

[32] Lee BH (2010). Enhancement of proteasome activity by a small-molecule inhibitor of USP14. Nature.

[33] Aleo E (2006). Identification of new compounds that trigger apoptosome-independent caspase activation and apoptosis. Cancer Res.

[34] Altun M (2011). Activity-based chemical proteomics accelerates inhibitor development for deubiquitylating enzymes. Chem Biol.

[35] Sakamoto KM (2001). Protacs: chimeric molecules that target proteins to the Skp1-Cullin-F box complex for ubiquitination and degradation. Proc Natl Acad Sci U S A.

[36] Bekes M (2022). PROTAC targeted protein degraders: the past is prologue. Nat Rev Drug Discov.

[37] Yoon H (2024). Induced protein degradation for therapeutics: past, present, and future. J Clin Invest.

[38] Qi S (2022). Targeting E2 ubiquitin-conjugating enzyme UbcH5c by small molecule inhibitor suppresses pancreatic cancer growth and metastasis. Mol Cancer.

[39] Hosseini SM (2019). E2 ubiquitin-conjugating enzymes in cancer: Implications for immunotherapeutic interventions. Clin Chim Acta.

[40] Ramatenki V (2015). Homology modeling and virtual screening of ubiquitin conjugation enzyme E2A for designing a novel selective antagonist against cancer. J Recept Signal Transduct Res.

[41] Matsumoto A (2014). High UBCH10 protein expression as a marker of poor prognosis in esophageal squamous cell carcinoma. Anticancer Res.

[42] Han SS (2011). [UbcH10 expression in hepatocellular carcinoma and its clinicopathological significance]. Nan Fang Yi Ke Da Xue Xue Bao.

[43] Qin T (2017). Exceptionally high UBE2C expression is a unique phenomenon in basal-like type breast cancer and is regulated by BRCA1. Biomed Pharmacother.

[44] Okamoto Y (2003). UbcH10 is the cancer-related E2 ubiquitin-conjugating enzyme. Cancer Res.

[45] Tokumoto M (2011). Cadmium toxicity is caused by accumulation of p53 through the down-regulation of Ube2d family genes in vitro and in vivo. J Toxicol Sci.

[46] Hou L (2018). UBE2D1 RNA expression was an independent unfavorable prognostic indicator in lung adenocarcinoma, but not in lung squamous cell carcinoma. Dis Markers.

[47] Shukla S (2014). KRAS protein stability is regulated through SMURF2: UBCH5 complex-mediated β-TrCP1 degradation. Neoplasia.

[48] Zhou W (2017). Neddylation E2 UBE2F promotes the survival of lung cancer cells by activating CRL5 to degrade NOXA via the K11 linkage. Clin Cancer Res.

[49] Yu B (2015). Oncogenesis driven by the Ras/Raf pathway requires the SUMO E2 ligase Ubc9. Proc Natl Acad Sci U S A.

[50] Chen S (2017). UBE2J2 promotes hepatocellular carcinoma cell epithelial-mesenchymal transition and invasion in vitro. Oncotarget.

[51] Falvey CM (2017). UBE2L6/UBCH8 and ISG15 attenuate autophagy in esophageal cancer cells. Oncotarget.

[52] Vila IK (2017). A UBE2O-AMPKα2 axis that promotes tumor initiation and progression offers opportunities for therapy. Cancer Cell.

[53] Chang R (2015). Upregulated expression of ubiquitin-conjugating enzyme E2Q1 (UBE2Q1) is associated with enhanced cell proliferation and poor prognosis in human hapatocellular carcinoma. J Mol Histol.

[54] Maeda H (2009). Ubiquitin-conjugating enzyme UBE2Q2 suppresses cell proliferation and is down-regulated in recurrent head and neck cancer. Mol Cancer Res.

[55] Ayesha AK (2016). UBE2S is associated with malignant characteristics of breast cancer cells. Tumour Biol.

[56] Lin M (2019). UBE2S mediates tumor progression via SOX6/β-catenin signaling in endometrial cancer. Int J Biochem Cell Biol.

[57] Li Z (2018). Ube2s stabilizes β-catenin through K11-linked polyubiquitination to promote mesendoderm specification and colorectal cancer development. Cell Death Dis.

[58] Wen M (2015). Elevated expression of UBE2T exhibits oncogenic properties in human prostate cancer. Oncotarget.

[59] Hu W (2016). UBE2T promotes nasopharyngeal carcinoma cell proliferation, invasion, and metastasis by activating the AKT/GSK3β/β-catenin pathway. Oncotarget.

[60] Hao J (2008). Elevated expression of UBE2T in lung cancer tumors and cell lines. Tumour Biol.

[61] Tsukamoto S (2008). Leucettamol A: a new inhibitor of Ubc13-Uev1A interaction isolated from a marine sponge, Leucetta aff. microrhaphis. Bioorg Med Chem Lett.

[62] Dalisay DS (2009). Absolute configuration of the alpha,omega-bifunctionalized sphingolipid leucettamol A from Leucetta microrhaphis by deconvoluted exciton coupled CD. J Nat Prod.

[63] Ceccarelli DF (2011). An allosteric inhibitor of the human Cdc34 ubiquitin-conjugating enzyme. Cell.

[64] Ushiyama S (2012). Manadosterols A and B, sulfonated sterol dimers inhibiting the Ubc13-Uev1A interaction, isolated from the marine sponge Lissodendryx fibrosa. J Nat Prod.

[65] Hodge CD (2015). Covalent inhibition of Ubc13 affects ubiquitin signaling and reveals active site elements important for targeting. ACS Chem Biol.

[66] Cheng J (2014). A small-molecule inhibitor of UBE2N induces neuroblastoma cell death via activation of p53 and JNK pathways. Cell Death Dis.

[67] Lee MJ (2024). UBE2N is essential for maintenance of skin homeostasis and suppression of inflammation. J Invest Dermatol.

[68] Chang JH (2012). Ubc13 maintains the suppressive function of regulatory T cells and prevents their conversion into effector-like T cells. Nat Immunol.

[69] Geisler S (2014). The ubiquitin-conjugating enzymes UBE2N, UBE2L3 and UBE2D2/3 are essential for Parkin-dependent mitophagy. J Cell Sci.

[70] Eddins MJ (2006). Mms2-Ubc13 covalently bound to ubiquitin reveals the structural basis of linkage-specific polyubiquitin chain formation. Nat Struct Mol Biol.

[71] Campbell SJ (2012). Molecular insights into the function of RING finger (RNF)-containing proteins hRNF8 and hRNF168 in Ubc13/Mms2-dependent ubiquitylation. J Biol Chem.

[72] Gallo LH (2017). The importance of regulatory ubiquitination in cancer and metastasis. Cell Cycle.

[73] Zhu Q (2020). UBE2N regulates paclitaxel sensitivity of ovarian cancer via fos/P53 axis. Onco Targets Ther.

[74] Yang B (2024). UBE2N promotes cell viability and glycolysis by promoting Axin1 ubiquitination in prostate cancer cells. Biol Direct.

[75] Dikshit A (2018). UBE2N promotes melanoma growth via MEK/FRA1/SOX10 signaling. Cancer Res.

[76] Yan H (2019). Inhibition of UBE2N-dependent CDK6 protein degradation by miR-934 promotes human bladder cancer cell growth. FASEB J.

[77] Barreyro L (2022). Blocking UBE2N abrogates oncogenic immune signaling in acute myeloid leukemia. Sci Transl Med.

[78] Zhang R (2004). DEG: a database of essential genes. Nucleic Acids Res.

[79] Bottomly D (2022). Integrative analysis of drug response and clinical outcome in acute myeloid leukemia. Cancer Cell.

[80] Strickson S (2013). The anti-inflammatory drug BAY 11-7082 suppresses the MyD88-dependent signalling network by targeting the ubiquitin system. Biochem J.

[81] Klomsiri C (2011). Cysteine-based redox switches in enzymes. Antioxid Redox Signal.

[82] McClellan AJ (2019). Cellular functions and molecular mechanisms of non-lysine ubiquitination. Open Biol.

[83] Zhou Z (2017). USP51 promotes deubiquitination and stabilization of ZEB1. Am J Cancer Res.

[84] Chen J (2023). USP51 promotes non-small cell lung carcinoma cell stemness by deubiquitinating TWIST1. J Transl Med.

[85] Li J (2023). USP51/PD-L1/ITGB1-deployed juxtacrine interaction plays a cell-intrinsic role in promoting chemoresistant phenotypes in non-small cell lung cancer. Cancer Commun (Lond).

[86] Mu M (2023). USP51 facilitates colorectal cancer stemness and chemoresistance by forming a positive feed-forward loop with HIF1A. Cell Death Differ.

[87] Zhou F (2023). The role of USP51 in attenuating chemosensitivity of lung cancer cells to cisplatin by regulating DNA damage response. Biotechnol Appl Biochem.

[88] Zhou F (2023). An integrative bioinformatics investigation and experimental validation of chromobox family in diffuse large B-cell lymphoma. BMC Cancer.

[89] Chen RH (2020). Cullin 3 and its role in tumorigenesis. Adv Exp Med Biol.

[90] Anderica-Romero AC (2013). Cullin 3 as a novel target in diverse pathologies. Redox Biol.

[91] Thorsteinsdottir U (1997). Overexpression of HOXA10 in murine hematopoietic cells perturbs both myeloid and lymphoid differentiation and leads to acute myeloid leukemia. Mol Cell Biol.

[92] Guo C (2020). Overexpression of HOXA10 is associated with unfavorable prognosis of acute myeloid leukemia. BMC Cancer.

[93] Shah CA (2011). HoxA10 regulates transcription of the gene encoding transforming growth factor beta2 (TGFbeta2) in myeloid cells. J Biol Chem.

[94] Wang H (2009). Constitutively active SHP2 cooperates with HoxA10 overexpression to induce acute myeloid leukemia. J Biol Chem.

[95] Al-Kershi S (2019). The stem cell-specific long noncoding RNA HOXA10-AS in the pathogenesis of KMT2A-rearranged leukemia. Blood Adv.

[96] Han Q (2023). HOXC13-driven TIMM13 overexpression promotes osteosarcoma cell growth. Cell Death Dis.

[97] Zhou S (2022). TIMM13 as a prognostic biomarker and associated with immune infiltration in skin cutaneous melanoma (SKCM). Front Surg.

[98] Moser B (2021). The ups and downs of STAT inhibition in acute myeloid leukemia. Biomedicines.

[99] Redell MS (2011). Stat3 signaling in acute myeloid leukemia: ligand-dependent and -independent activation and induction of apoptosis by a novel small-molecule Stat3 inhibitor. Blood.

[100] Rezvani K, Barrett J (2014). STAT3: the “Achilles” heel for AML?. Blood.

[101] Shi Y (2018). Roles of STAT3 in leukemia (Review). Int J Oncol.

[102] Spiekermann K (2001). Constitutive activation of STAT transcription factors in acute myelogenous leukemia. Eur J Haematol.

[103] Wingelhofer B (2018). Implications of STAT3 and STAT5 signaling on gene regulation and chromatin remodeling in hematopoietic cancer. Leukemia.

[104] Yoyen-Ermis D (2019). Myeloid maturation potentiates STAT3-mediated atypical IFN-γ signaling and upregulation of PD-1 ligands in AML and MDS. Sci Rep.

[105] Rushworth SA (2014). Identification of Bruton’s tyrosine kinase as a therapeutic target in acute myeloid leukemia. Blood.

[106] Pillinger G (2015). Targeting BTK for the treatment of FLT3-ITD mutated acute myeloid leukemia. Sci Rep.

[107] Byrd JC (2013). Targeting BTK with ibrutinib in relapsed chronic lymphocytic leukemia. N Engl J Med.

[108] Bennett J, Starczynowski DT (2022). IRAK1 and IRAK4 as emerging therapeutic targets in hematologic malignancies. Curr Opin Hematol.

[109] Melgar K (2019). Overcoming adaptive therapy resistance in AML by targeting immune response pathways. Sci Transl Med.

[110] Smith MA (2019). U2AF1 mutations induce oncogenic IRAK4 isoforms and activate innate immune pathways in myeloid malignancies. Nat Cell Biol.

[111] Bennett J (2023). Paralog-specific signaling by IRAK1/4 maintains MyD88-independent functions in MDS/AML. Blood.

[112] Parrondo RD (2023). IRAK-4 inhibition: emavusertib for the treatment of lymphoid and myeloid malignancies. Front Immunol.

[113] Gimenez N (2020). Targeting IRAK4 disrupts inflammatory pathways and delays tumor development in chronic lymphocytic leukemia. Leukemia.

[114] Falini B (2005). Cytoplasmic nucleophosmin in acute myelogenous leukemia with a normal karyotype. N Engl J Med.

[115] Ranieri R (2022). Current status and future perspectives in targeted therapy of NPM1-mutated AML. Leukemia.

[116] Falini B (2020). NPM1-mutated acute myeloid leukemia: from bench to bedside. Blood.

[117] Carvalho G (2007). Inhibition of NEMO, the regulatory subunit of the IKK complex, induces apoptosis in high-risk myelodysplastic syndrome and acute myeloid leukemia. Oncogene.

[118] Di Francesco B (2022). NF-κB: a druggable target in acute myeloid leukemia. Cancers (Basel).

[119] Vu LP (2017). Functional screen of MSI2 interactors identifies an essential role for SYNCRIP in myeloid leukemia stem cells. Nat Genet.

[120] Herrejon Chavez F (2023). RNA binding protein SYNCRIP maintains proteostasis and self-renewal of hematopoietic stem and progenitor cells. Nat Commun.

[121] French ME (2021). Emerging functions of branched ubiquitin chains. Cell Discov.

[122] Tubio-Santamaria N (2021). Immunoproteasome function in normal and malignant hematopoiesis. Cells.

[123] Basler M, Groettrup M (2021). On the role of the immunoproteasome in protein homeostasis. Cells.

[124] van den Eshof BL (2021). The function of immunoproteasomes-an immunologists’ perspective. Cells.

[125] Rouette A (2016). Expression of immunoproteasome genes is regulated by cell-intrinsic and -extrinsic factors in human cancers. Sci Rep.

[126] Jenkins TW (2021). Activity of immunoproteasome inhibitor ONX-0914 in acute lymphoblastic leukemia expressing MLL-AF4 fusion protein. Sci Rep.

[127] Niewerth D (2013). Higher ratio immune versus constitutive proteasome level as novel indicator of sensitivity of pediatric acute leukemia cells to proteasome inhibitors. Haematologica.

[128] Tubio-Santamaria N (2023). Immunoproteasome function maintains oncogenic gene expression in KMT2A-complex driven leukemia. Mol Cancer.

[129] Muchamuel T (2009). A selective inhibitor of the immunoproteasome subunit LMP7 blocks cytokine production and attenuates progression of experimental arthritis. Nat Med.

[130] Lam SS (2015). Directed evolution of APEX2 for electron microscopy and proximity labeling. Nat Methods.

[131] Jones EL (2021). TRIM21/Ro52 - roles in innate immunity and autoimmune disease. Front Immunol.

[132] Foss S (2019). TRIM21-from intracellular immunity to therapy. Front Immunol.

[133] Alomari M (2021). TRIM21 - a potential novel therapeutic target in cancer. Pharmacol Res.

[134] Liu YX (2023). TRIM21 is a druggable target for the treatment of metastatic colorectal cancer through ubiquitination and activation of MST2. Cell Chem Biol.

[135] Chen X (2022). The emerging roles of TRIM21 in coordinating cancer metabolism, immunity and cancer treatment. Front Immunol.

[136] Chen XQ (2023). Protein homeostasis in aging and cancer. Front Cell Dev Biol.

[137] Lv X (2023). Modulation of the proteostasis network promotes tumor resistance to oncogenic KRAS inhibitors. Science.

[138] Sniegocka M (2022). Understanding ER homeostasis and the UPR to enhance treatment efficacy of acute myeloid leukemia. Drug Resist Updat.

[139] Martelli AM (2020). The unfolded protein response: a novel therapeutic target in acute leukemias. Cancers (Basel).

[140] Ojha R, Amaravadi RK (2017). Targeting the unfolded protein response in cancer. Pharmacol Res.

[141] Hidalgo San Jose L (2020). Modest declines in proteome quality impair hematopoietic stem cell self-renewal. Cell Rep.

[142] Chua BA, Signer RAJ (2020). Hematopoietic stem cell regulation by the proteostasis network. Curr Opin Hematol.

[143] Feral K (2021). ER Stress and unfolded protein response in leukemia: friend, foe, or both?. Biomolecules.

[144] Sun T (2020). The role of ubiquitination and deubiquitination in cancer metabolism. Mol Cancer.

[145] Aliabadi F (2021). Ubiquitin-proteasome system and the role of its inhibitors in cancer therapy. Open Biol.

[146] McEwan WA (2013). Intracellular antibody-bound pathogens stimulate immune signaling via the Fc receptor TRIM21. Nat Immunol.

[147] Fletcher AJ (2015). Sequential ubiquitination and deubiquitination enzymes synchronize the dual sensor and effector functions of TRIM21. Proc Natl Acad Sci U S A.

[148] Anandapadamanaban M (2019). E3 ubiquitin-protein ligase TRIM21-mediated lysine capture by UBE2E1 reveals substrate-targeting mode of a ubiquitin-conjugating E2. J Biol Chem.

[149] Sabile A (2006). Regulation of p27 degradation and S-phase progression by Ro52 RING finger protein. Mol Cell Biol.

[150] Fletcher AJ, James LC (2016). Coordinated neutralization and immune activation by the cytosolic antibody receptor TRIM21. J Virol.

[151] Zhang J (2022). Inhibition of the CDK2 and cyclin A complex leads to autophagic degradation of CDK2 in cancer cells. Nat Commun.

[152] Chen J (2023). TRIM21 enhances bortezomib sensitivity in multiple myeloma by halting prosurvival autophagy. Blood Adv.

[153] Reddy BA (2014). Nucleotide biosynthetic enzyme GMP synthase is a TRIM21-controlled relay of p53 stabilization. Mol Cell.

[154] Guha A (2019). Integrated regulation of HuR by translation repression and protein degradation determines pulsatile expression of p53 under DNA damage. iScience.

[155] Liu J (2023). The ubiquitin ligase TRIM21 regulates mutant p53 accumulation and gain of function in cancer. J Clin Invest.

[156] Chen X (2021). Trim21-mediated HIF-1α degradation attenuates aerobic glycolysis to inhibit renal cancer tumorigenesis and metastasis. Cancer Lett.

[157] Lin HP (2016). Destabilization of fatty acid synthase by acetylation inhibits de novo lipogenesis and tumor cell growth. Cancer Res.

[158] Gong J (2023). TRIM21-promoted FSP1 plasma membrane translocation confers ferroptosis resistance in human cancers. Adv Sci (Weinh).

[159] Zhang H (2014). STAT3 restrains RANK- and TLR4-mediated signalling by suppressing expression of the E2 ubiquitin-conjugating enzyme Ubc13. Nat Commun.

[160] Zhang H (2018). Genetic rescue of lineage-balanced blood cell production reveals a crucial role for STAT3 antiinflammatory activity in hematopoiesis. Proc Natl Acad Sci U S A.

[161] Ferrington DA, Gregerson DS (2012). Immunoproteasomes: structure, function, and antigen presentation. Prog Mol Biol Transl Sci.

[162] Winter MB (2017). Immunoproteasome functions explained by divergence in cleavage specificity and regulation. Elife.

[163] Kumar R (2023). Prognostic association of immunoproteasome expression in solid tumours is governed by the immediate immune environment. Mol Oncol.

[164] Heink S (2005). IFN-gamma-induced immune adaptation of the proteasome system is an accelerated and transient response. Proc Natl Acad Sci U S A.

[165] Tundo GR (2021). At the cutting edge against cancer: a perspective on immunoproteasome and immune checkpoints modulation as a potential therapeutic intervention. Cancers (Basel).

