# Supplementary material for: Ubiquitin-conjugating enzyme UBE2N modulates proteostasis in immunoproteasome-positive acute myeloid leukemia
Source: J Clin Invest. 2025 May 15;135(10):e184665. doi: 10.1172/JCI184665 (PMC12077902; doi:10.1172/JCI184665)
Supplement: Supplemental data [file jci-135-184665-s032.pdf]

Supplemental Figure 1

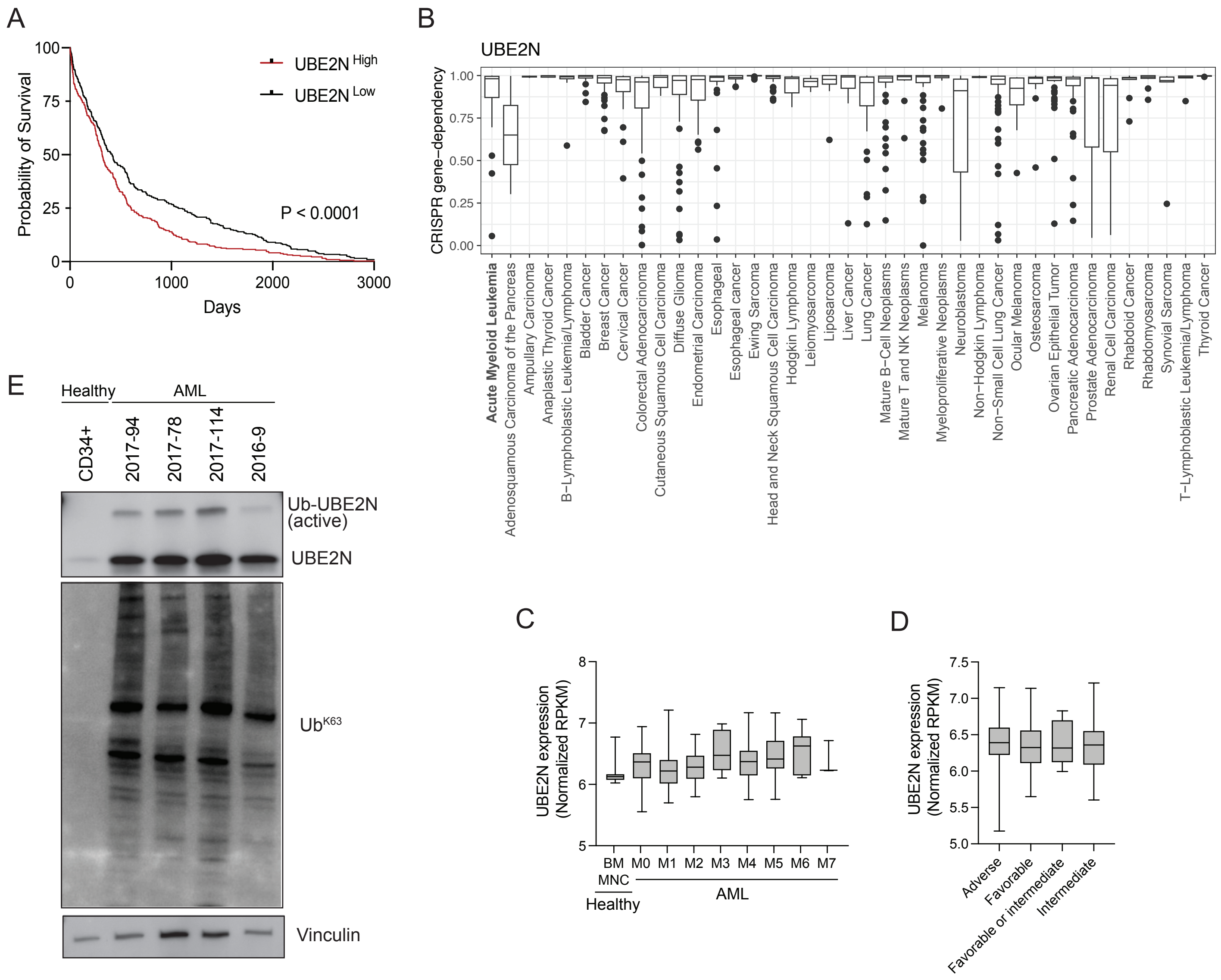

Supplemental Figure 1. UBE2N catalytic function is essential for AML. **(A)** Survival curve for AML patients classified as above or below the median in UBE2N mRNA expression (Beat-AML 2.0). Mantel-Conx test was used to determine the significance. **(B)** CRISPR-Cas9 screen (DepMap) to identify the dependency of UBE2N in human cancer cell lines. Each dot in the graph represents an individual cancer cell line. **(C-D)** mRNA expression level of UBE2N in AML patients and healthy BM MNCs (Beat AML 2.0) based on FAB classification (C) or on risk stratification (D). Error bars represent the standard error of the mean. **(E)** Activated UBE2N (Ub-UBE2N) and total UBE2N protein expression along with global K63-linked ubiquitination in patient-derived AML (PD-AML) cells and healthy CD34+ cells.

Supplemental Figure 2

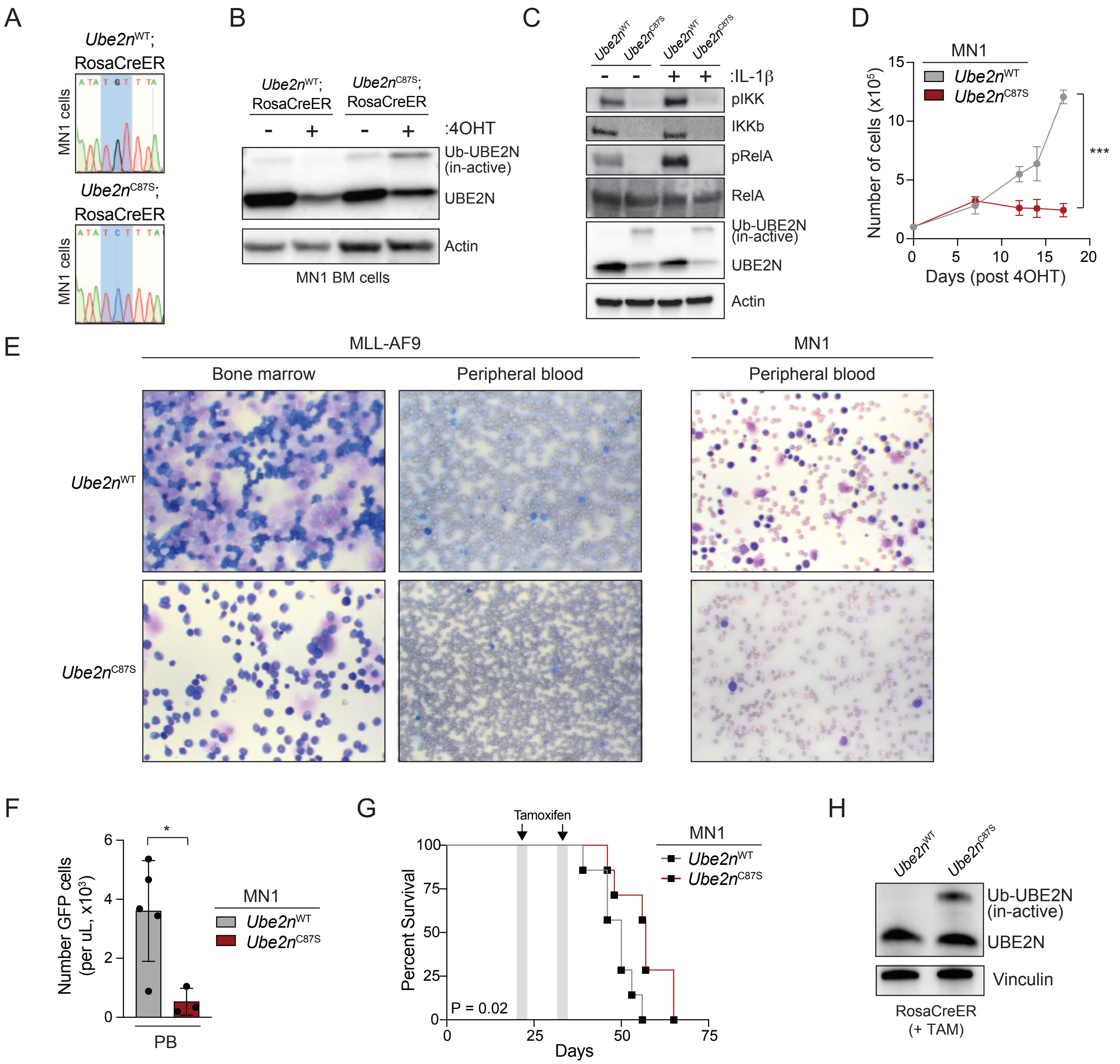

Supplemental Figure 2. UBE2N catalytic function is essential for AML. (A) DNA sanger sequencing was conducted to confirm the recombination from Cysteine (TGT) to Serine (TCT) upon Cre expression in MN1 transduced *Ube2n*^{WT} or *Ube2n*^{C87S} cells. **(B)** Immunoblots of *Ube2n*$^{+/fl}$; Rosa26CreERT2 (*Ube2n*^{WT}) or *Ube2n*$^{C87S/fl}$ Rosa26CreERT2 (*Ube2n*^{C87S}) cells transduced with MN1 and then treated with 1 μM 4OHT for 48 hours. **(C)** Immunoblots of *Ube2n*^{WT} or *Ube2n*^{C87S} cells expressing MN1 were evaluated following stimulation with IL-1β. **(D)** Cell proliferation of MN1 transduced *Ube2n*^{WT} or *Ube2n*^{C87S} AML cells pre-treated with 1 μM 4OHT for 48 hours. Cell numbers were counted by trypan blue exclusion (n = 3). 2-way ANOVA was used to determine significance. Error bars represent the standard error of the mean. ***, P< 0.001. **(E)** *Ube2n*^{WT} or *Ube2n*^{C87S} MLL-AF9 AML cells and *Ube2n*^{WT} or *Ube2n*^{C87S} MN1 AML cells were transplanted into lethally-irradiated recipient WT mice. Shown are Wright-Giemsa staining of bone marrow cytospins and peripheral blood smears from the indicated mice at the time of death. **(F)** Number of leukemic MN1 cells (GFP+) in peripheral blood at time of death. (*Ube2n*^{WT}, n = 5; *Ube2n*^{C87S}, n = 3) Student's t test (unpaired, two-tailed) was used to determine significance. Error bars represent the standard error of the mean. *, P < 0.05. **(G)** Kaplan-Meier survival analysis of mice transplanted with *Ube2n*^{WT} or *Ube2n*^{C87S} MN1 cells (GFP+). Tamoxifen was injected 5 constitutive days in week 3 and week 5 post transplantation to induce expression of *Ube2n*^{C87S} (n = 7/group). Log-rank (Mantel-Cox) test was used to determine the significance. **(H)** Immunoblots of BM cells from healthy *Ube2n*^{WT} or *Ube2n*^{C87S} mice (following treatment with Tamoxifen).

Supplemental Figure 3

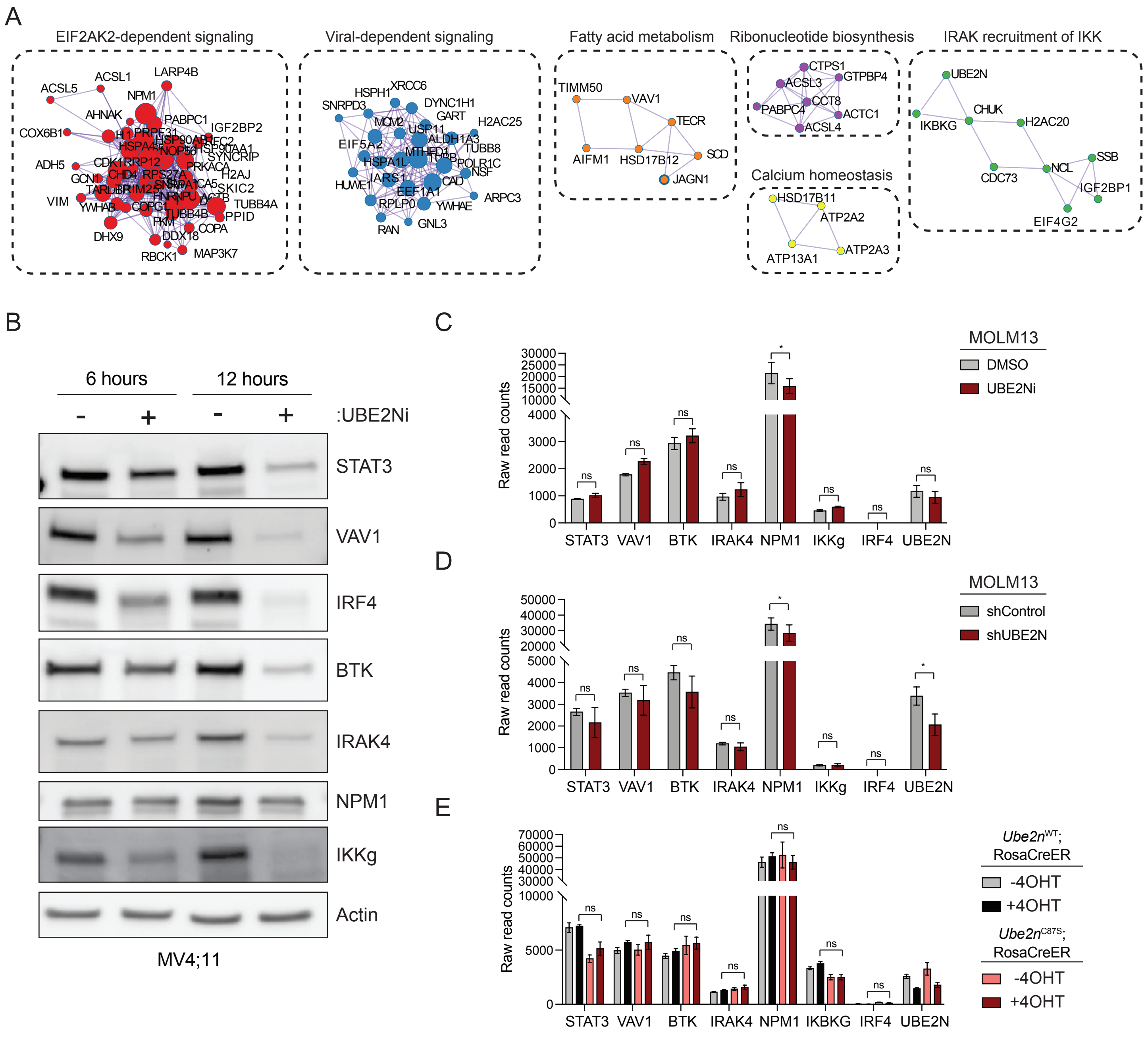

Supplemental Figure 3. UBE2N regulates oncoprotein levels but not transcript levels. (A) Signaling networks of UBE2N-dependent substrates. The size of the dots indicated the fold-change (related to Figure 3D,E) **(B)** Immunoblots of MV4;11 cells treated with UBE2N inhibitor (5 μM) for 6 hours and 12 hours. **(C)** Normalized raw read counts of the indicated genes from an RNA-sequencing analysis of MOLM13 cells treated with DMSO or UBE2N inhibitor (UBE2Ni) (2 μM) for 24 hours (n = 3). **(D)** Normalized raw read counts of the indicate genes from an RNA-sequencing analsyis of MOLM13 cells expressing shControl or shUBE2N. **(E)** Normalized raw read counts of the indicated genes from an RNA-sequencing analysis of MLL-AF9-expressing *Ube2n*^{WT} or *Ube2n*^{C87S} AML cells treated with control or 50 nM 4OHT for 48 hours. Student's t test (unpaired, two-tailed) was used to determine significance. Error bars represent the standard error of the mean. *, P < 0.05; **, P < 0.01; ***, P< 0.001; ns, not significant.

Supplemental Figure 4

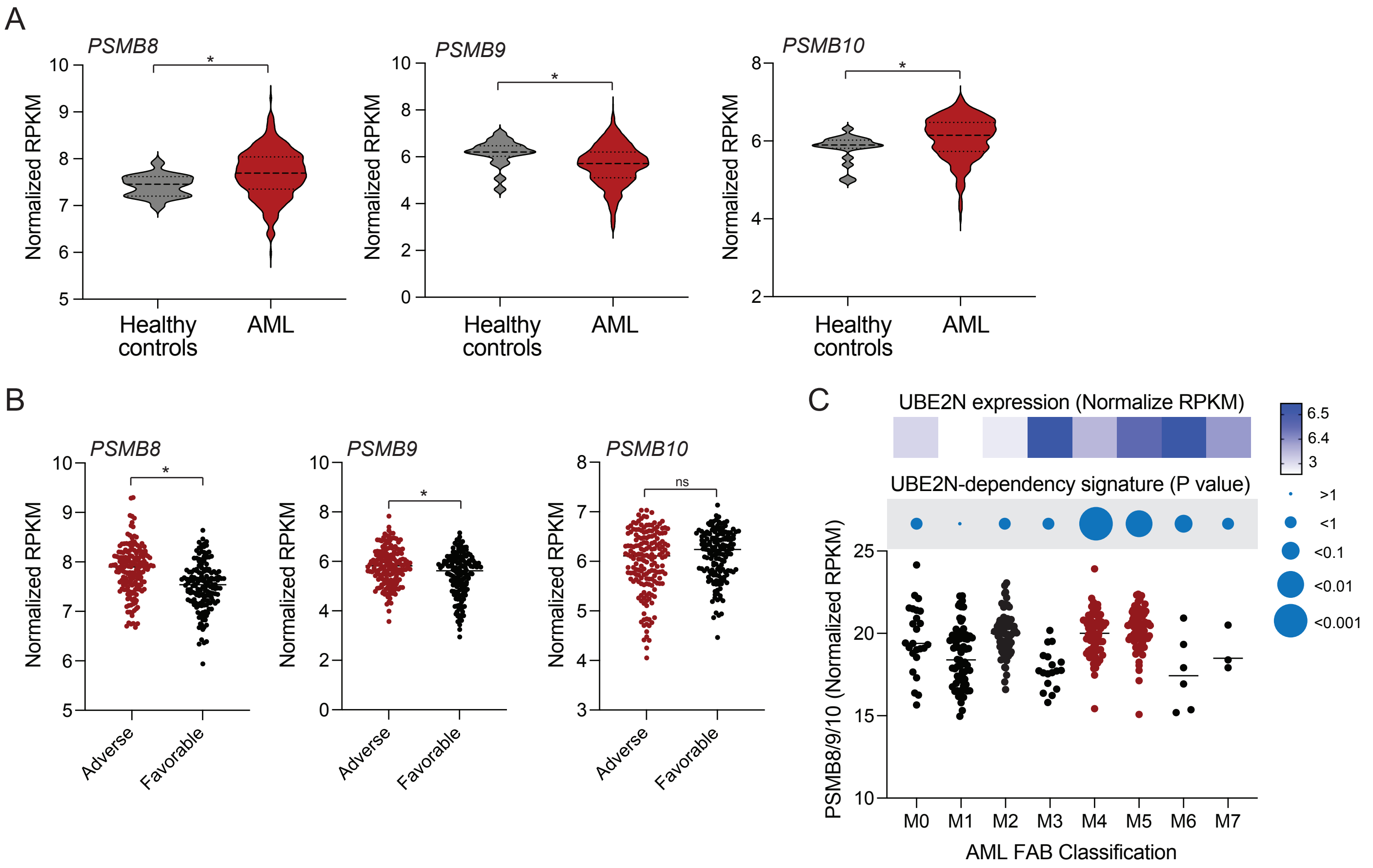

Supplemental Figure 4. Immunoproteasome genes are highly expressed in AML patients and correlate with UBE2N activation. (A) mRNA expression of immunoproteasome genes (*PSMB8, PSMB9, PSMB10*) in AML patients and healthy controls (Beat-AML 2.0). Student's t test (unpaired, two-tailed) was used to determine significance. Error bars represent the standard error of the mean. *, P < 0.05. **(B)** mRNA expression of Immunoproteasome genes (*PSMB8, PSMB9, PSMB10*) in AML patient samples stratified on risk classification (Beat AML 2.0). Student's t test (unpaired, two-tailed) was used to determine significance. Error bars represent the standard error of the mean. *, P < 0.05; ns, not significant. **(C)** The correlation of compositive immunoproteasome genes expression (*PSMB8, PSMB9, PSMB10*) and AML FAB classification. UBE2N dependency signature was determined in PD-AML samples treated with UBE2N inhibitor. RNA-sequencing was performed at baseline and the dependency signature was derived from sensitive and resistant samples. The UBE2N-dependency signature is represented by blue dots above. The size of the blue dot is corresponding to each P value. UBE2N gene expression was examined across the AML FAB classification. Heat map represents the average UBE2N mRNA expression across the FAB subtypes.

Supplemental Figure 5

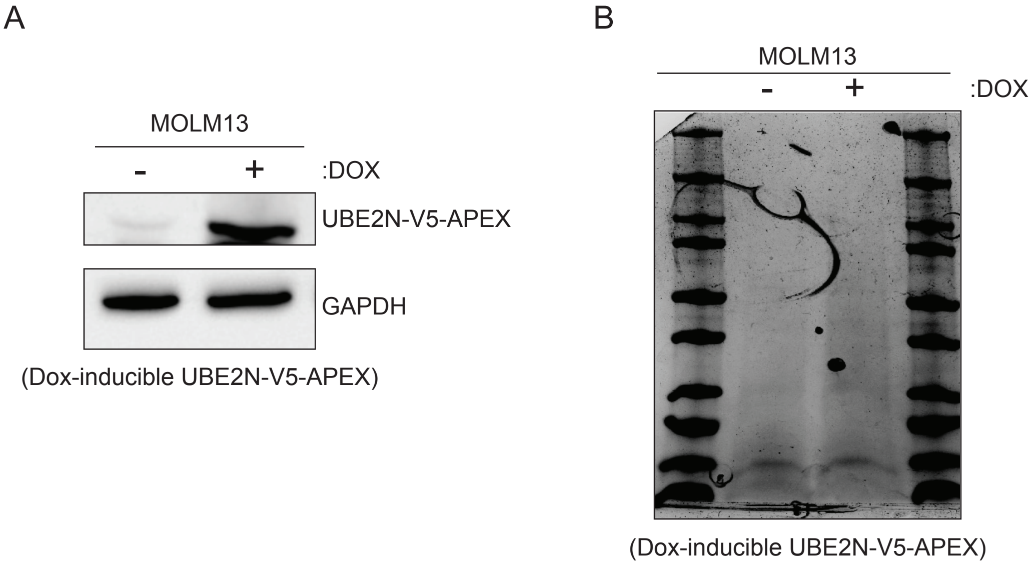

Supplemental Figure 5. APEX proximity labeling assay identifies TRIM21 as an interacting E3 ligase of UBE2N in AML cells. (A) Immunoblots of V5 tag in MOLM13 cells expressing UBE2N-V5 tag fused with APEX2 protein. 0.5 μg/ml of doxycycline was added to cell culture media for 24 hours. **(B)** Imperial staining after biotin-labeling of MOLM13 cells expressing UBE2N-V5 tag fused with APEX2 protein.

Supplemental Figure 6

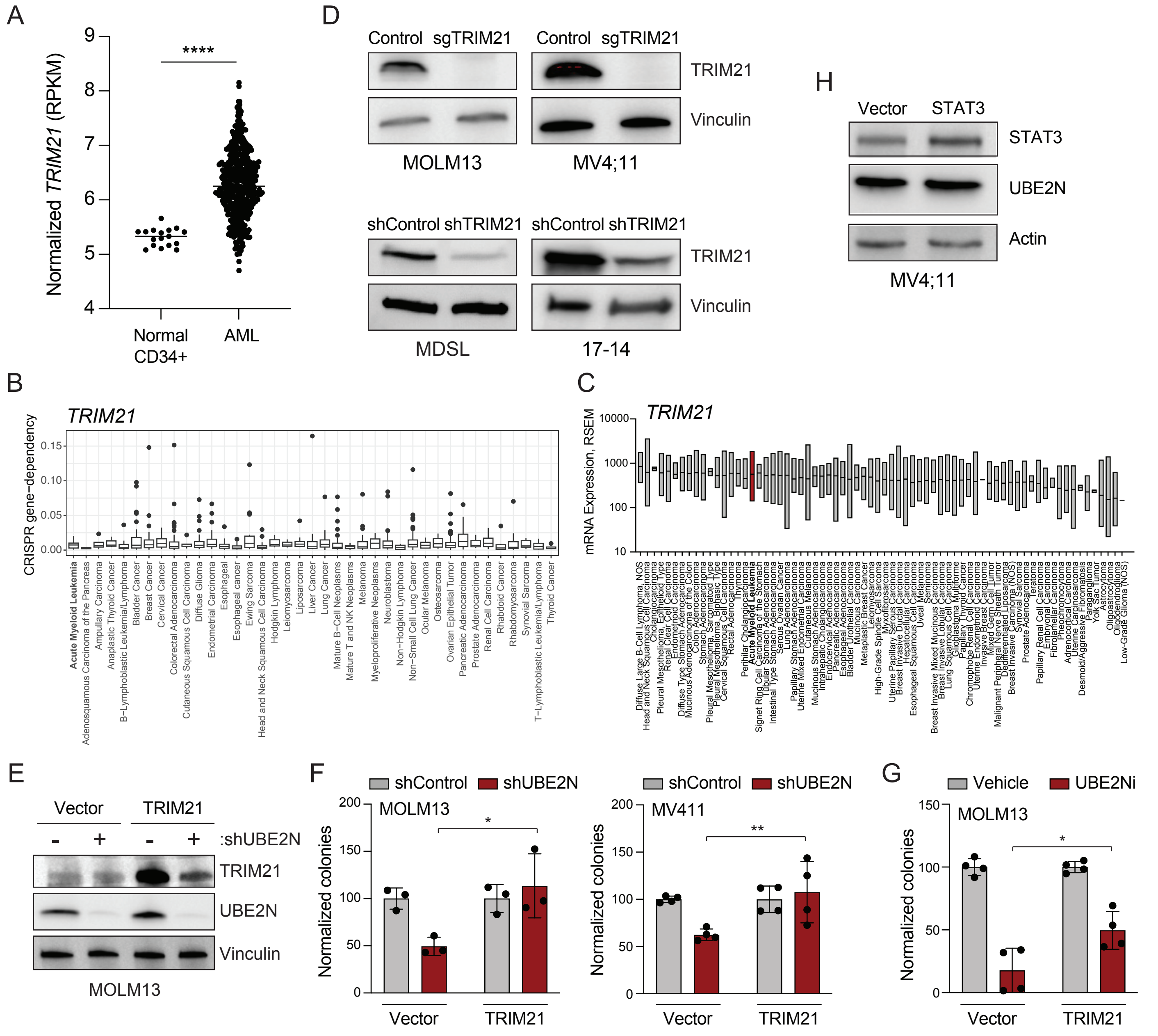

Supplemental Figure 6. Characterization of TRIM21 in AML. (A) mRNA expression of TRIM21 in AML patients and healthy controls (Beat-AML 2.0). Student's t test (unpaired, two-tailed) was used to determine significance. Error bars represent the standard error of the mean. *, P < 0.05; **, P < 0.01; ***, P< 0.001; ****, P< 0.0001. **(B)** CRISPR-Cas9 screen (DepMap) to identify the dependency of TRIM21 in human cancer cell lines. Each dot in the graph represents an individual cancer cell line. **(C)** TRIM21 mRNA expression in human cancer samples from cBioportal. **(D)** Immunoblots of TRIM21 in MOLM13, MV4;11, MDSL, and PD-AML (17-14) cells. TRIM21 in MOLM13 and MV4;11 was deleted by CRISPR/Cas9. TRIM21 in MDSL and PD-AML (17-14) cells was knocked down with nontargeting shRNA (shControl) or shTRIM21. **(E)** Immunoblot analysis of MOLM13 cells expressing shRNAs targeting UBE2N (+) or scrambled control (-) and TRIM21 cDNA or empty vector control. **(F)** Colony forming assay of MOLM13 or MV4;11 cells expressing shRNAs targeting UBE2N (+) or scrambled control (-) and TRIM21 cDNA or empty vector control. **(G)** Colony forming assay of MOLM13 cells expressing TRIM21 cDNA or empty vector control and treated with UBE2N inhibitor (UBE2Ni)(5 μM). **(H)** Immunoblot of STAT3 in MV4;11 cells transduced with empty vector or constitutive active STAT3.

Supplemental Methods

Cells

MOLM13 cells were purchased from AddexBio. MV4;11 cells were provided by L. Grimes (Cincinnati, CCHMC). HEK293T cells were provided from S. Wells (Cincinnati, CCHMC). MDS-L cells were provided by K. Tohyama (Kawasaki Medical School, Okayama, Japan). THP1, HL60, and TF1 were purchased from ATCC. SET2, OCI-AML2 and OCI-AML3 were purchased from DSMZ. MOLM-14 were provided by Dr. Neil Shah Lab (University of California San Francisco, CA). HEL cells were provided by Dr. Gang Huang (UT Health San Antonio, TX). All cells were authenticated by STR Profiling Service from ATCC. MOLM13, MOLM14, and SET2 cells were cultured in RPMI-1640 with 20% of fetal bovine serum (FBS) and 1% penicillin/streptomycin. MV4;11, HL60, THP1, HEL cells were cultures in RPMI-1640 with 10% of FBS and 1% penicillin/streptomycin. OCI-AML2 and OCI-AML3 cells were cultured in MEMalpha media with 20% FBS and 1% penicillin/ streptomycin. TF1 cells were cultured in RPMI-1640 with 10% of FBS and 1% penicillin/streptomycin, and 10 ng/ml of human IL3. MDS-L cells were cultured in RPMI with 10% FBS and 1% penicillin/ streptomycin and 10ng/ml recombinant human IL-3. MLL-AF9 transduced murine BM cells were cultured in Iscove's Modified Dulbecco's Medium supplemented with 10% FBS and 1% penicillin/ streptomycin and 10ng/ml of each murine IL-3, human IL-6, and murine SCF. Other murine HSPC cells were cultured in Iscove's Modified Dulbecco's Medium supplemented with 10% FBS and 1% penicillin/ streptomycin and 100 ng/ml of each murine SCF, human G-SCF, and human TPO.

Patient samples

Bone marrow (BM) samples from patients with AML were obtained with written informed consent and approved by the institutional review board of Cincinnati Children's Hospital Medical Center and University of Cincinnati, or from the Eastern Cooperative Oncology Group (ECOG). These samples had been obtained within the framework of routine diagnostic BM aspirations after written informed consent in accordance with the Declaration of Helsinki. De-identified leukemic cells from peripheral blood and bone marrow of patients with AML were obtained at CCHMC following consent under the IRB approved Study ID # 2008-0021. To obtain sufficient cell numbers, the AML samples were first expanded in immunocompromised mice and then viably frozen. Herein, these samples are referred to as patient-derived AML (PD-AML) cells. Human $CD34^+$ cells were either obtained from the Translational Research Development Support Laboratory of CCHMC under an approved Institutional Review Board protocol or purchased from Yale Cooperative Center of Excellence in Hematology, Yale University. The cells were cultured in X-VIVO-10 (Lonza cat# BE04-380Q), 20% BIT 9500 (Stemcell Technologies cat#09500), 1% penicillin/ streptomycin, 50 ng/ml of each human SCF, human TPO, human FLT3-L, 25 ng/ml of each human IL-3, human IL-6, and human G-CSF. Sex or gender information was not available. For patients' characteristics, see Supplemental Table 7.

Mice

All animals were bred and housed in the Association for Assessment and Accreditation of Laboratory Animal Care-accredited animal facility of Cincinnati Children's Hospital Medical Center. All mice were kept on C57BL/6N background. UBE2N conditional knock-in mouse was generated and purchased from Ozgene (Australia). These mice were bred to Rosa26-CreERT2 from Jackson lab in order to have tamoxifen-inducible excision of the floxed sites. The mutated C87S mice contains cDNA of WT UBE2N followed by polyA sequence between the introns of exon 1 and exon 2. This WT cDNA+polyA cassette is surrounded by LoxP sites. Downstream of this WT cDNA cassette, there is exon 2 which Cysteine 87 is mutated to Serine. These mice were crossed with *Ube2n*$^{fl/fl}$;Rosa26CreERT2 mice gifted from Dr. Watowich from Texas MD Anderson, Houston. We used *Ube2n*$^{+/fl}$;Rosa26CreERT2 and *Ube2n*$^{+/+}$;Rosa26CreERT2 as WT group and *Ube2n*$^{C87S/fl}$;Rosa26CreERT2 as C87S group for the study. Male and female recipient and donor mice were used.

Reagents

The UBE2N inhibitor UC-764865 (UC-65) was previously described(1). UC-764865 was synthesized at Wuxi AppTec. Chemical structure of the compound was analyzed by nuclear magnetic resonance (NMR). ONX-0914 (PR-957) was purchased from Selleckchem (cat #S7172). MG132 (Z-Leu-Leu-Leu-al) was purchased from Sigma (cat #C2211). Bafilomycin A1 was purchased from Sigma (cat#B1793). All LC-MS grade solvents were obtained from J.T. Baker (Fisher Scientific). shRNAs used in the study are described in Supplemental Table 10.

Retroviral expression of oncogenes to murine BM cells

Femur, tibia and humerus bones were harvested from *Ube2n*$^{+/fl}$;Rosa26CreERT2 or *Ube2n*$^{C87S/fl}$;Rosa26CreERT2 mice. The bones were crushed, and filtered in PBS, and then treated with red blood cell lysis buffer (Lysing buffer Cat #555899), followed by PBS wash. Lineage negative bone marrow cells were isolated using the Easy Sep Mouse Hematopoietic Progenitor Cell Enrichment Kit (Stem Cell Technologies, #19856) and incubated at 37C, 5% CO2 for overnight. Following day, the isolated HSPCs were then transduced with concentrated retrovirus encoding MLL-AF9, MN1, RUNX1-D171N, RUNX1-RUNX1T1, FLT3-ITD using retronectin coated plates. Briefly, the day before transduction, wells of the 6 -well plate were coated with 2 ml of 25 ug/ml retronectin in PBS at 4C and incubated for overnight. The following day, the retronectin was removed and after gentle wash, with IMDM, the concentrated retrovirus in IMDM was spun at 800 x g for 2 hours at 32C to allow virus to attach to the wells. After spinoculation, the media in the wells are discarded, and 1.5 - 3 million HSPCs in were plated in the wells with new fresh IMDM with 10% FBS+ 1% P/S + 100 ng/ml TPO, G-CSF, and SCF with 0.8 ug/ml polybrene. Due to the low transduction efficiency, some oncogenes required additional spinoculation with fresh virus (3000 rpm, 32C, 2 hours) the following day. For MLL-AF9 cells, transduced HSPCs were serially plated in methocult three times to select the transduced cells, and then sorted for GFP+ cells. The other oncogenes were transduced and sorted for GFP+ cells 3 days-7 days post infection. For MLL-AF9 cells, 1

million cells with 0.5 million bone marrow mononuclear cells from Boy J donor cells were transplanted to the lethally irradiated recipient Boy J mouse (n = 10/ group). For other oncogenes, after transducing HSPCs, the GFP+ cells were sorted using Sony SH800 or Sony MA900 cell sorter within a week, and colony forming ability assay was performed. For MN1 transplantation, 3000 cells of MN1 (GFP+) cells were mixed with 0.5 million bone marrow mononuclear cells from Boy J donor cells and transplanted to the lethally irradiated recipient Boy J mouse (n = 10/ group). However, 3 mice from each group were sickly euthanized before tamoxifen administration, so those 6 mice are excluded from the evaluation.

Bone marrow transplantation assays

Bone marrow cells harvested from femur, tibia and humerus bones from either *Ube2n*$^{fl/+}$; Rosa26CreERT2 (*Ube2n*^{WT}) or *Ube2n*$^{f/C87S}$;Rosa26CreERT2 (*Ube2n*^{C87S}) mouse, and after passing through the filter, the BM cells were treated with red blood cell lysis buffer and incubated for 8 minutes. The cells were washed 2 times using PBS after the RBC lyse, and the BM mononuclear cells were prepared. 1 million BM mononuclear cells were transplanted to the lethally irradiated recipient BoyJ mice (n = 10/ group). Tamoxifen is administered beginning 4 weeks post-transplantation, and 1mg of tamoxifen in corn oil was injected intraperitoneally for 5 constitutive days in week 4 and 6 post-transplantation. The transplanted mice were monitored for the blood counts every 4 weeks using Oxford Science GENESIS using mouse measurement mode. In the week of 15 post-transplantation, all the mice were euthanized with carbon dioxide following the AVMA Guidelines for the Euthanasia of Animals for the analysis.

Cell transductions

For knockdown studies, UBE2N and TRIM21 shRNA template were cloned into the pLKO.1 TRC cloning vector (Addgene: #10878) and pLKO.1 TRC cloning vector. The puromycin gene in pLKO.1 was replaced with GFP. pLKO.1 TRC control was used as non-silencing control (Addgene: #10879). For the APEX2 study, gblock containing the sequence of UBE2N-V5-APEX2 was cloned into backbone vector pCW57.1-eGFP (Gifted from Volk lab, Cincinnati, CCHMC) using restriction enzymes MluI and NheI sites. All the plasmids used are listed in Supplemental Table 11. For production of lentiviral particles, lentiviral constructs were transfected together with packaging vectors into 293T producer cells using TransIT transfection reagent (Mirus cat#MIR 2306). The media was exchanged with fresh media 24 hours later, and the supernatants were harvested 48 hours after the transduction. The harvested lentivirus containing supernatants were spinoculated with cells in addition of 0.8 ug/ml polybrene at 32C, 3000 rpm, 90 minutes.

Analysis of cell morphology

Peripheral blood smears were spread onto a glass slide and stained with Wright-Giemsa stain using an automatic slide stainer (Hematek, Siemens). Bone marrow cells were cytospun in coated glass slides for 5 minutes at 500 rpm with low acceleration. The slides were stained with Giemsa staining using an automatic stainer. Pictures were acquired with an Olympus LC30 camera and Motic BA310 microscope at the indicated magnification.

Generation of CRISPR knock-out cells

MOLM13 and MV411 cells were transfected with three sgRNAs targeting exon 2 of TRIM21 purchased from Synthego (UUCCCCCUGUGUGCCCUCUC, AUGUUGGCUAGCUGUCGAUU, CUGCCAGGAAUGCAUCUCUC) together with Cas9 2NLS Nuclease (Synthego) using Neon transfection system (Invitrogen). After incubating the cells for 2 days, they were plated in 96 well plates in order to have single clone per well. We collected the clones that grew, and were screened for TRIM21 ablation by immunoblotting as well as PCR amplification followed by sanger sequencing.

Colony forming assays

Colony-forming assay was done using either Methocult H4434 Classic (Stem Cell Technologies, Cat# 04434) for human cells, and Methocult GF M3434 (Stem Cell Technologies, Cat# 03434) for mouse BM cells. The cells in Methocult were plated in SmarDish 6 well plates (Stem Cell Technologies, Cat# 27371) and kept in 37℃ and 5% CO2. Colonies were imaged and counted using STEMvision after 7-14 days.

Xenograft assays

250,000 cells of TRIM21^{WT} and TRIM21^{KO} MV411 cells were transplanted to unconditioned NSGS mice. The mice were monitored over time, and when the mice are moribund, the mice were euthanized and BM, spleen, PB was collected. The complete blood counts were performed using PB, flow cytometry analysis for human CD33 and human CD45 was conducted using bone marrow mononuclear cells, spleen cells, and peripheral blood.

Cell proliferation and cell viability assays

For MLL-AF9 transduced cells and HSPCs, the cells were the appropriate density (up to 0.5 million/ml) and treated with 0.5 μM 4OHT and the cell numbers were counted using trypan blue exclusion assay over 4-5 days. For MN1 transduced cells, the cells were treated with 1 μM 4OHT the cell numbers were counted using trypan blue exclusion assay over 17 days. When the cells became confluent, the cells were resuspended in the new fresh media (without 4OHT) with 1:1 dilution. For MTS assay in PDX cells, it has been conducted in previously published paper(1). Briefly, the cells were plated in duplicate in a 96 well plate in IMDM containing 20% FBS plus 10 ng/ml of each SCF, IL3, IL6, TPO, and FLT3. UBE2N inhibitor UC-764864 was added at the indicated concentrations and cells were incubated with drug for 3 days, at which point cell viability was measured by MTS according to manufacturer's instructions.

Flow cytometry analysis

To determine the engraftment of the MLL-AF9 or MN1 transduced UBE2N WT or C87S BM cells, the engraftment of MLL-AF9 or MN1 cells (both GFP+) was determined by flow cytometry analysis in BD FACS Canto and BD FACS Canto II. To analyze HSPC frequency in total and competitive transplants, the stained cells were analyzed in BD LSR Fortessa. For the stem cell and stem progenitor cell population, the following panel is used: DAPI, CD45.1, CD45.2, Lineage, cKit, Sca1, CD135, CD150, CD48. For the committed progenitor cell population, the following panel is used: DAPI, CD45.1, CD45.2, Lineage, cKit, Sca1, CD16/32, CD34. The antibodies used for these panels are listed in the Supplemental Table 12.

Immunoblotting and co-immunoprecipitation

For immunoblotting, cells were lysed in RIPA buffer (50 mM Tris-HCl, 150 mM NaCl, 1 mM ethylenediaminetetraacetic acid (EDTA), 1% Triton X-100, and 0.1% sodium dodecyl sulfate (SDS)) with phenylmethylsulfonyl fluoride (PMSF), protease inhibitor (Sigma-Aldrich Cat #11836153001), and phosphatase inhibitors (Sigma Aldrich Cat # P5726 and #P0044). The lysates were incubated on ice for 30 minutes, followed by centrifugation to clear the lysates. The protein concentration was measured by BCA assay and 10 ug-30 ug of protein was used for each sample. The lysates were mixed with sample buffer and denatured by boiling at 95℃ for 5 minutes, followed by SDS-polyacrylamide gel electrophoresis (SDS-PAGE). Then transfer step was conducted using trans-blot turbo transfer system (BIO-RAD), and immunoblotted with the antibodies listed in Supplemental Table 13. For exogenous co-immunoprecipitation, HEK293T cells were transfected using Transit LT (MIRUS Cat #MIR2300) transfection reagent, and the cells were incubated for 48 hours (changed media after 24 hours) and lysed in Triton Lysis Buffer (30mM Tris-HCL pH7.4, 300mM NaCl, 1mM EDTA, 1% Triton, 0.5% IGEPAL, Protease inhibitor (Halt), Phosphatase Inhibitor Cocktail 2 and 3 (Sigma Aldrich), 0.75mM PMSF, and 0.25mM DTT). The lysates were kept in rotator at 4C for 1 hour, followed by centrifugation to clear the lysates. 1 mg of total protein measured by BCA assay were filled up to 1ml with lysis buffer and incubated with anti-FLAG M2 affinity gel (Sigma Cat #A2220) at 4C overnight. After immunoprecipitation, the beads were washed using Triton Wash Buffer (30mM Tris-HCL pH7.4, 500mM NaCl, 1mM EDTA, 1% Triton, 0.5% IGEPAL), leaving on a rotator at 4C for 15min and repeating this step for additional 3 times. The bound proteins were eluted from the bead using 5x sample buffer followed by boiling the samples at 95C for 5 min. The eluted samples were analyzed with SDS-PAGE and immunoblotting. For denatured endogenous co-immunoprecipitation, MV4;11 cells were treated with 200 nM ONX-0914 in combination with DMSO or 5 μM UBE2N inhibitor (UC-65) for 24 hours and then collected in lysis buffer (30 mM Tris-HCl, 300 mM NaCl, 1 mM EDTA, 1% Triton, 0.1% IGEPAL, 1x Halt protease inhibitor, phosphatase inhibitor cocktail 2, phosphatase inhibitor cocktail 3, 0.75 mM PMSF, 0.25 mM DTT). The lysates were kept in a rotator at 4C for 1 hour and passed through a 25-gauge 1ml syringe 15 times followed by centrifugation to clear the lysate. 1 mg of total protein measured by BCA assay was filled up to 1ml with lysis buffer and boiled at 95C for 10 min. After the lysate cooled down, the lysates were incubated in pre-cleared Protein A/G PLUS-agarose beads (Santa Cruz, SC-2003) for 1 hour. The beads and the lysates were spun down and the supernatant (precleared lysate) was incubated with fresh precleared Protein A/G PLUS-agarose beads with NPM1 antibody or STAT3 antibody and rotated at 4C overnight. Following day, the beads were washed using washing buffer (30 mM Tris HCl, 500 mM NaCl, 1 mM EDTA, 1% Triton, 0.1% IGEPAL) for 15 minutes, and this step was repeated for additional 3 times. After washing, the beads were eluted in 20 ul of sample buffer and boiled at 95C for 10 minutes with frequent vortexing. Finally, the beads were isolated using Bio-Rad micro bio-spin chromatography columns (Bio-Rad, #7326204), and the samples

were analyzed with SDS-PAGE and immunoblotting. For IL-1β stimulation, mouse MLL-AF9-expressing AML cells were treated with ethanol control or 0.5 μM 4OHT for 48 hours, and then the cells were washed and treated with 10 ug/ml of IL-1 β for 30 minutes, followed by lysate collection. The antibodies used for immunoblotting experiments are listed in the Supplemental Table 13.

RNA-sequencing

MLL-AF9 transduced $Ube2n^{WT}$ or $Ube2n^{C87S}$ cells were treated in either 100% ethanol control or 50nM 4OHT for 48 hours in same density in triplicates. The cells were collected, and RNA was extracted using ZYMO RESEARCH Quick-RNA Miniprep kit (cat #R1055). After the confirmation of the RNA quality using Agilent 2100 Bioanalyzer, the libraries were prepared with polyA selection using the Truseq RNA Library Prep Kit, and the libraries were sequenced at an average depth of 30M paired-end 100bp nucleotide reads. After the quality of reads was examined using FastQC (v0.11.7, https://www.bioinformatics.babraham.ac.uk/projects/fastqc), paired-end reads were aligned against mouse mm10 genome (iGenome,https://support.illumina.com/sequencing/sequencing_software/igenome.html) using HISAT2 (v2.0.5,http://daehwankimlab.github.io/hisat2). The raw gene counts were calculated using featureCounts (v1.5.2, http://subread.sourceforge.net/) and normalized using edgeR (v3.16.5, https://bioconductor.org/packages/release/bioc/html/edgeR.html) in iGEAK (https://pubmed.ncbi.nlm.nih.gov/30841853/). Differentially expressed genes were predicted using limma/voom (v3.30.6, https://bioconductor.org/packages/release/bioc/html/limma.html). For RNA-sequencing methods of 1) UBE2N inhibitor sensitive or resistant PDX cells, 2) MOLM13 cells expressing shControl vs shUBE2N, and 3) MOLM13 cells treated with DMSO or UBE2N inhibitor (UC-764864), it has been previously published(1).

Ubiquitin-enriched mass spectrometry

MV411 cells were infected with lentiviral constructs of shControl, shUBE2N, or shTRIM21, followed by selection by sorting for mCherry-positive cells. Each condition was prepared in triplicate, and 10,000,000 cells were collected. After probe sonication in a urea buffer as described in the Cell Signaling Technology (CST) protocol (cat #59322), a 660nm protein assay was performed on the samples. 1.3mg of protein was removed from each sample the volume was adjusted to 1ml with the lysis buffer described in the protocol. The cells were lysed, reduced with DTT, alkylated with IAA and digested with trypsin, followed by ubiquitin enrichment using HS Ub/SUMO (K-e-GG) enrichment kit (Cell Signaling, cat #59322). After digestion with trypsin, the peptides were desalted and concentrated by running the samples through a C18 Sep-Pak (WAT051910) as described in the CST 5622S protocol and dried in a SpeedVac. Total ubiquitin (GG-K) peptides were enriched by passing the peptides over the HS (K-e-GG) magnetic beads according to the protocol. The eluted ubiquitin peptides were passed over a C18 stage tip for desalting and dried in a SpeedVac. The samples were reconstituted in 0.1% Formic acid (FA) and analyzed by nanoLC-MS/MS

(Thermo Orbitrap Eclipse) with the LC, elution, and mass spectrometry parameters for label-free quantitation all documented previously(2). The data were searched against a combined database of common contaminants and the uniprot *homo sapiens* database with Proteome Discoverer ver 3.0 using the Sequest HT search algorithm (Thermo Scientific) and a LFQ quantitation workflow incorporating the IMP-ptmRS node to calculate probabilities of site modifications. Ratios were calculated using the pairwise method with normalization to all ubiquitin peptides. P-values were calculated using the t-test background method of Proteome Discoverer.

Total proteomics mass spectrometry

MV4;11 cells were treated with DMSO or 5 μM UBE2Ni for 24 hours (three replicates for each group and each group contains 3 million cells). After 24 hours, the cells were washed with PBS for 3 times. The samples were solubilized in 100 μl of Thermo Easypep lysis buffer (A45735) with 1 μl of universal nuclease added. The samples were then reduced, alkylated and digested with LysC/trypsin according to the Easypep MS sample prep kit (A40006) instructions. The peptides were desalted using the columns provided in the Easypep MS sample prep kit according to the provided instructions and then dried in a speed vac. The samples were resuspended in 0.1% Formic acid (FA) and 10% of each sample was analyzed by nanoLC-MS/MS (Orbitrap Eclipse). The results were searched against a combined contaminant database plus the swissprot homo sapiens database using the Sequest HT search algorithm and the LFQ quantitation workflow in Proteome discoverer ver 3.0 (Thermo scientific). Abundances were normalized to total peptides and ratios were calculated using the pairwise method. P values were calculated using the background-based method in PD.

CRISPR activation screen

A pooled lentiviral preparation of the Calabrese P65-HSF activation library was purchased from Addgene (Cat # 92379-LV). MOLM13 cells were transduced with a lentiviral dCas9-VP64-GFP (Addgene Cat #61422) and sorted for GFP expression. GFP positive cells were next transduced in triplicates with pooled lentiviral library, and were selected in puromycin for 7 days at an MOI of 0.3. After puromycin selection, the cells were treated with DMSO or 2.5 μM the UBE2N inhibitor (UC-65) for 7 to 14 days. UC-65 or DMSO was treated in every 3 days after media change. At day 7 and 14 of the treatment, genomic DNA was harvested using the DNeasy Blood and Tissue kit (Qiagen Cat #69504). The genomic library was amplified and indexed following the Broad Institute protocol for PCR of sgRNAs for Illumina sequencing. The barcoded libraries were pooled and sequenced on an Illumina MiSeq at a read depth of 15M with 250 bp pair ends. We used the MAGeCK pipeline for data processing and analysis (v0.5.9; https://sourceforge.net/p/mageck/wiki/Home/). First, original paired-end reads were automatically trimmed using MAGeCK. Next, MAGeCK-count was used to calculate sgRNA read counts using trimmed reads and human CRISPRa sgRNA library (https://media.addgene.org/data/plasmids/92/92379/92379-attachment_IRjBnhb_GMxk.txt). Finally,

MAGeCK-test was used to predict gene rescue from the resulting count matrix after considering a control sample. The default FDR threshold for gene test is 0.25.

Immunoproteasome functional assay

Immunoproteasome function was assessed using Immunoproteasome Activity Fluorometric Assay Kit I (UBPBio, #J4160). Briefly, PDX cells were lysed in lysis buffer (40mM Tris, pH7.2, 50mM NaCl, 2mM beta-ME, 2mM ATP, 5mM MgCl2, 10% glycerol). The lysates were incubated on ice for 30 minutes, followed by centrifugation to clear the lysates. The protein concentration was measured by BCA assay and 25 ug of protein was used for each sample. 2x Ac-ANW-AMC substrate was prepared in proteasome assay buffer, and warmed at 37C water bath for 10 minutes. 25ug of PDX lysates in 50ul and 50ul of 2x Ac-ANW-AMC substrate were mixed in black 96 well plate with flat and clear bottom (Corning #3631) and immediately read in a plate reader with excitation and emission filters at 360/40 and 460/30 nm respectively. The kinetics were measured for 30 minutes with 1 minute interval.

APEX2 proximity labeling assay

APEX2 fused with V5 tagged UBE2N was cloned into doxycycline-inducible lentiviral backbone vector pCW57.1-eGFP (provided by Volk lab, CCHMC). The plasmid was transduced to MOLM13 cells and GFP-positive cells were sorted and expanded. 20,000,000 cells were prepared for each condition. The cells were treated with either control or 0.5 ug/ml doxycycline for 24h, and the cells were treated with biotin phenol for 30 minutes followed by hydrogen peroxide to activate the APEX2 protein. After 30 seconds of adding hydrogen peroxide, the reaction was immediately quenched by adding quenching reagent (5mM Trolox, 10mM Sodium Azide, 10mM Sodium Ascorbate) in ice-cold PBS and the cells were washed 3 times. The cells were lysed in RIPA buffer (50mM Tris-HCl pH8, 150 mM NaCl, 0.1% w/v SDS, 0.5% v/v Sodium Deoxycholate, 1% v/v Triton X-100) with protease inhibitor and quenchers on ice for 4^0C followed by sonication. After the cell lysates were clarified with high-speed centrifuge, the excess biotin was removed by 7kDa desalting column (Thermofisher Cat #89889). The lysates flow through was concentrated with Amicon 3kDa centrifugal filter unit (Millipore sigma Cat #UFC500324) and then mixed with magnetic neutravidin beads and rotated at 4C overnight. The lysates with neutravidin beads were washed in successive order with RIPA + quencher, 1M KCl, 0.1M Na_2CO_3, 1M Urea in 10mM Tris-HCl, and RIPA. After the wash, 60ul of Laemmli sample buffer containing 2mM biotin was added to the beads and gently shaken for 5 min then the samples were centrifuged at 4000 x g for 2 min. The supernatant was removed to a new tube and concentrated in a SpeedVac to about 40ul. The samples were then run 1.5cm into an Invitrogen 4-12% B-T gel using MOPS buffer with molecular weight marker lanes in between. The sections were excised, reduced with DTT, alkylated with IAA, and digested overnight with trypsin as described previously(3). After trypsin digestion, the peptides were extracted, dried in a SpeedVac and reconstituted in 7 ul of 0.1% Formic acid (FA). 5.5 uL of each sample was analyzed by nanoLC-MS/MS (Thermo Orbitrap Eclipse) and was searched against the homo sapiens database using Proteome

discoverer ver 2.4, the Sequest HT search algorithm (Thermo Scientific) and quantitation workflow as described previously(2). The data was normalized to all proteins.

Statistics

The number of animals, cells, and experimental/biological replicates can be found in the figure legends. Differences among multiple groups were assessed by one-way analysis of variance (ANOVA) followed by Tukey's multiple comparison posttest for all possible combinations. Comparison of two groups was performed using the Mann-Whitney test or the Student's *t* test (unpaired, two tailed) when sample size allowed. A P value less than 0.05 was considered significant. Unless otherwise specified, results are depicted as the mean ± standard deviation or standard error of the mean. A normal distribution of data was assessed for data sets >30. For correlation analysis, Pearson correlation coefficient (r) was calculated. D'Agostino and Pearson and Shapiro-Wilk tests were performed to assess data distributions. For Kaplan-Meier analysis, Mantel-Cox test was used. All graphs and analysis were generated using GraphPad Prism software or using the package ggplot2 from R(4).

Study approval

All bone marrow samples from patients with AML were obtained with written informed consent and approved by the institutional review board of Cincinnati Children's Hospital Medical Center and University of Cincinnati, or from the Eastern Cooperative Oncology Group (ECOG). These samples had been obtained within the framework of routine diagnostic BM aspirations after written informed consent in accordance with the Declaration of Helsinki. De-identified leukemic cells from peripheral blood and bone marrow of patients with AML were obtained at CCHMC following consent under the IRB approved Study ID # 2008-0021. All mice were bred and housed in the Association for Assessment and Accreditation of Laboratory Animal Care-accredited animal facility of Cincinnati Children's Hospital Medical Center. The animal handling was strictly followed IACUC protocols approved by the Institutional Animal Care and Use Committee at Cincinnati Children's Hospital Medical Center (protocol no. IACUC 2019-0072, 2022-0054).

Supplemental References

1. Barreyro L, Sampson AM, Ishikawa C, Hueneman KM, Choi K, Pujato MA, et al. Blocking UBE2N abrogates oncogenic immune signaling in acute myeloid leukemia. *Sci Transl Med.* 2022;14(635):eabb7695.
2. Bennett J, Ishikawa C, Agarwal P, Yeung J, Sampson A, Uible E, et al. Paralog-specific signaling by IRAK1/4 maintains MyD88-independent functions in MDS/AML. *Blood.* 2023;142(11):989-1007.
3. Eismann T, Huber N, Shin T, Kuboki S, Galloway E, Wyder M, et al. Peroxiredoxin-6 protects against mitochondrial dysfunction and liver injury during ischemia-reperfusion in mice. *Am J Physiol Gastrointest Liver Physiol.* 2009;296(2):G266-74.
4. Wickham H. *Ggplot2: Elegant Graphics for Data Analysis.* 2009.
