# Supplementary material for: Ubiquitin-conjugating enzyme UBE2N modulates proteostasis in immunoproteasome-positive acute myeloid leukemia
Source: J Clin Invest. 2025 May 15;135(10):e184665. doi: 10.1172/JCI184665 (PMC12077902; doi:10.1172/JCI184665)

Figure 1G

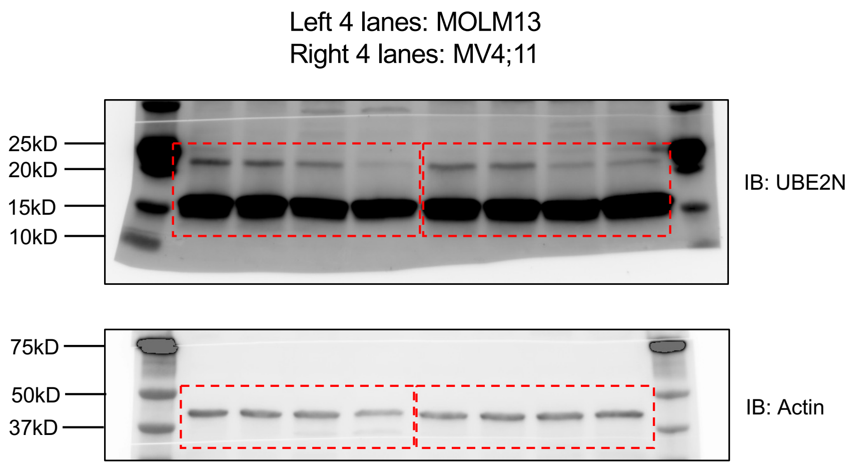

Figure 1F

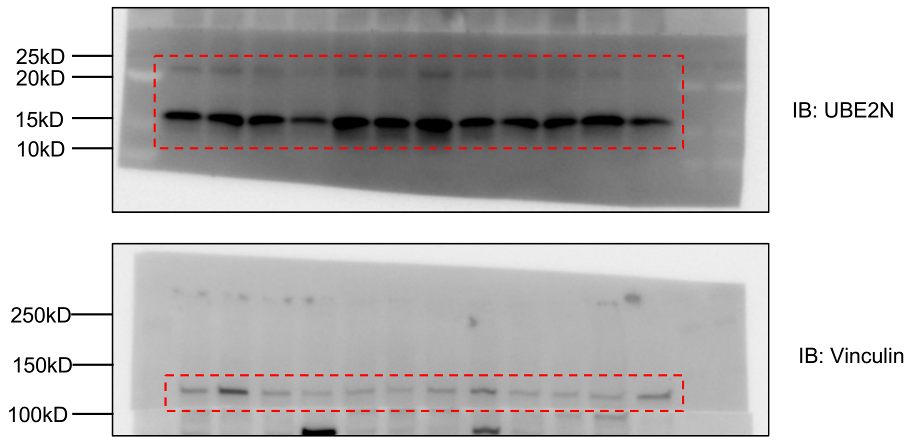

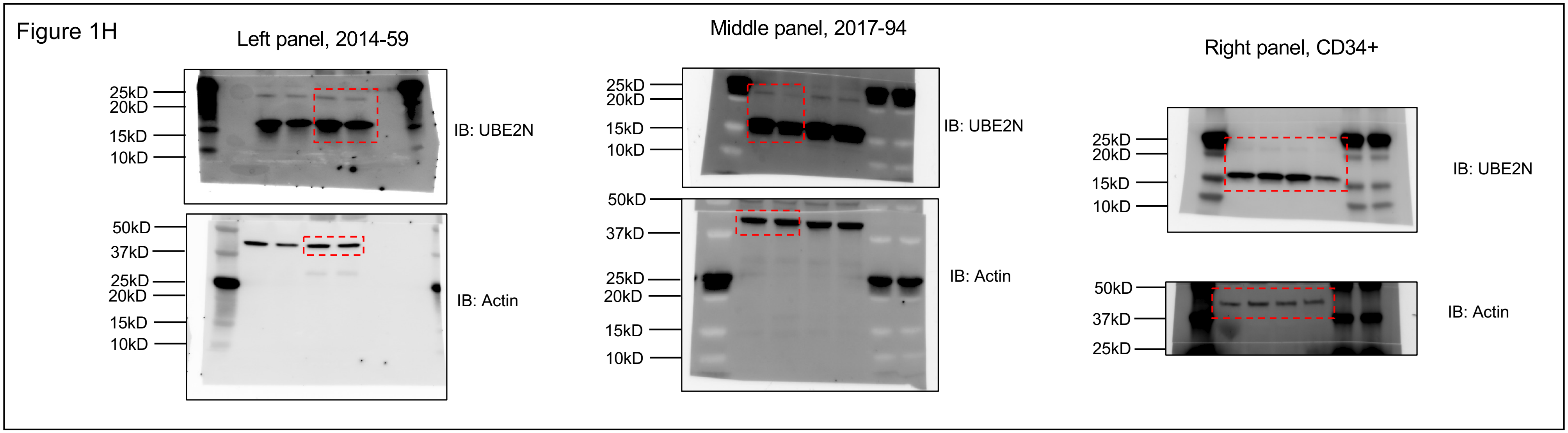
Figure 1H
Left panel, 2014-59
25kD
20kD
15kD
10kD
IB: UBE2N
50kD
37kD
25kD
20kD
15kD
10kD
IB: Actin
Middle panel, 2017-94
25kD
20kD
15kD
10kD
IB: UBE2N
50kD
37kD
25kD
20kD
15kD
10kD
IB: Actin
Right panel, CD34+
25kD
20kD
15kD
10kD
IB: UBE2N
50kD
37kD
25kD
IB: Actin

Figure 2E

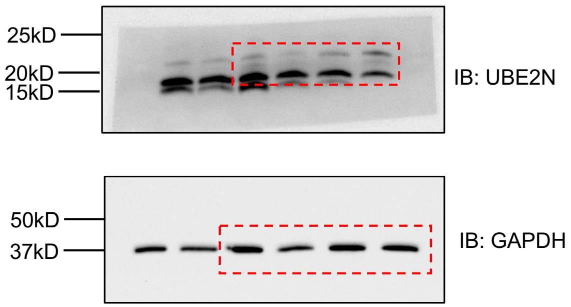

Figure 4A

From left: MOLM13, MV4;11, HL60

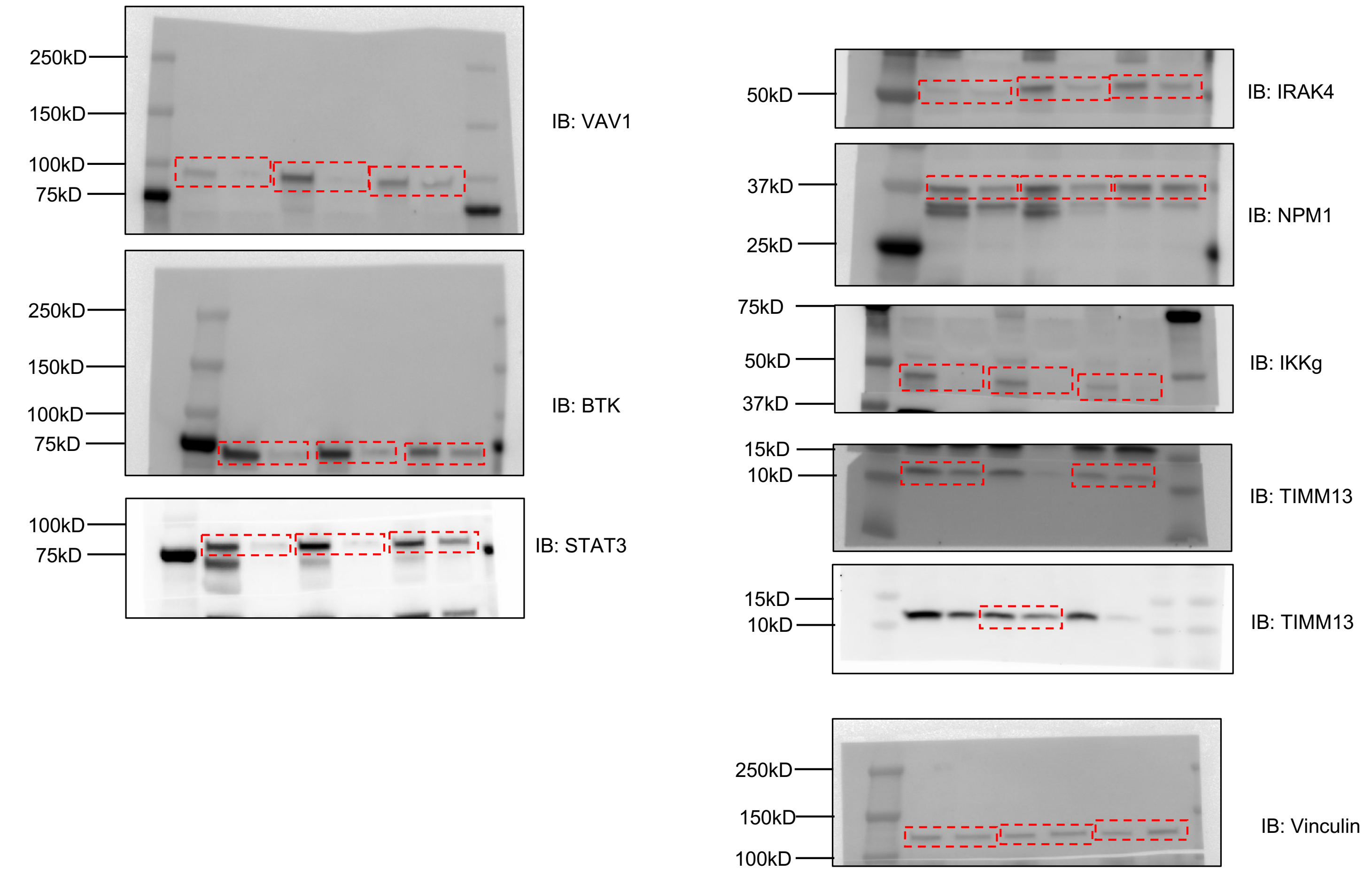

Figure 4B

Left panel: 2017-94, Right panel: 2016-1

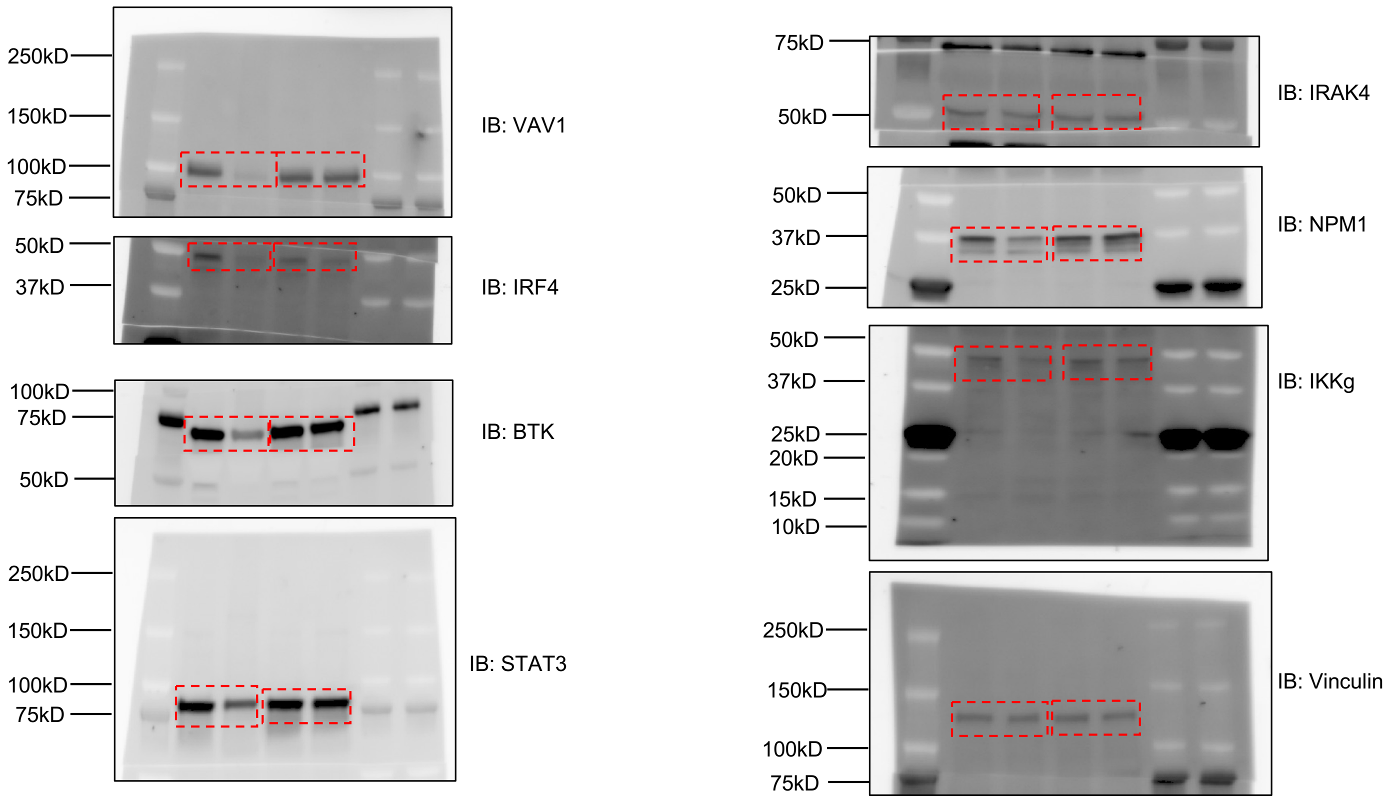

Figure 4B

Middle panel, 2017-78

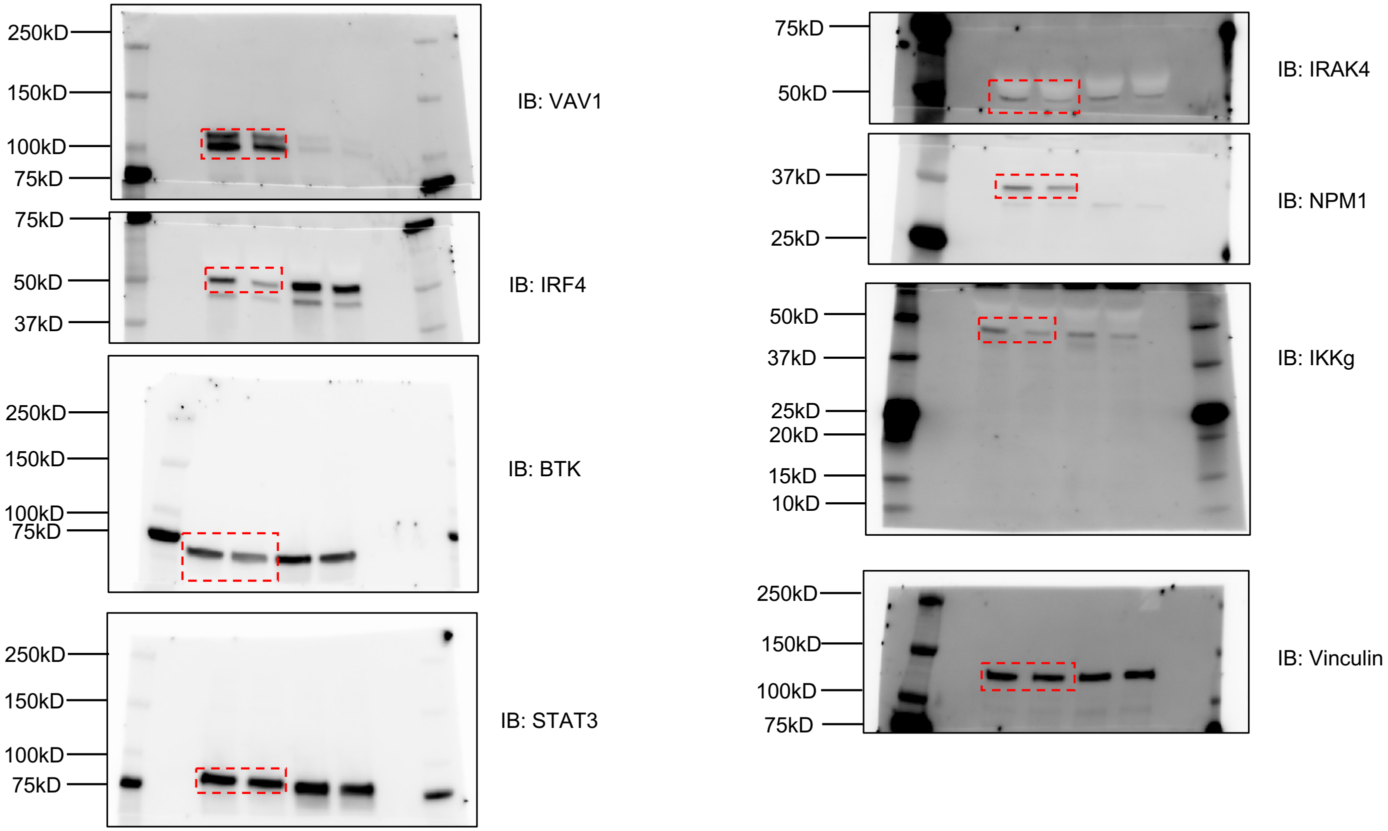

Figure 4C

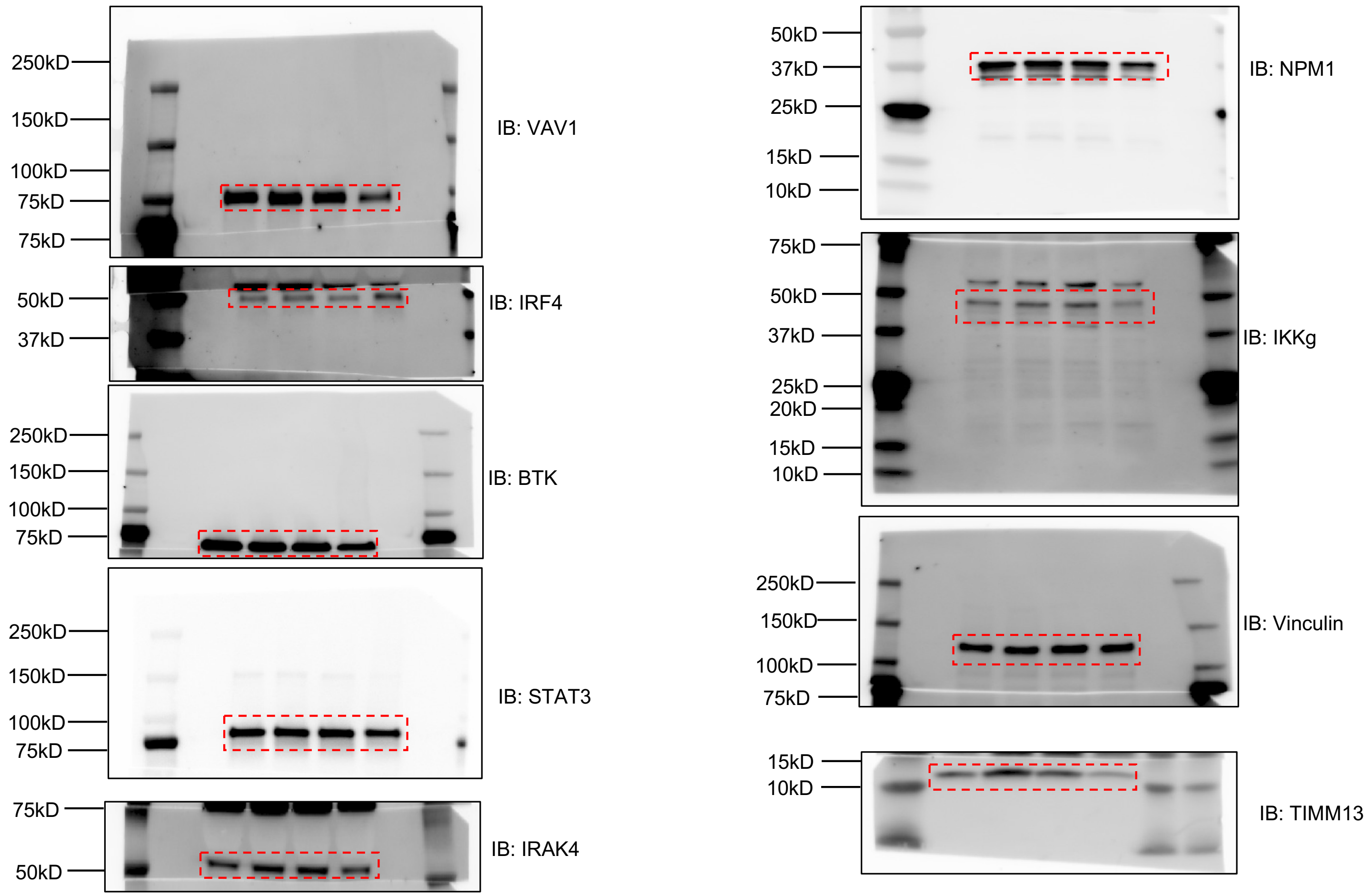
250kD
150kD
100kD
75kD
75kD
IB: VAV1
50kD
37kD
IB: IRF4
250kD
150kD
100kD
75kD
IB: BTK
250kD
150kD
100kD
75kD
IB: STAT3
75kD
50kD
IB: IRAK4
50kD
37kD
25kD
15kD
10kD
IB: NPM1
75kD
50kD
37kD
25kD
20kD
15kD
10kD
IB: IKKg
250kD
150kD
100kD
75kD
IB: Vinculin
15kD
10kD
IB: TIMM13

Figure 4D

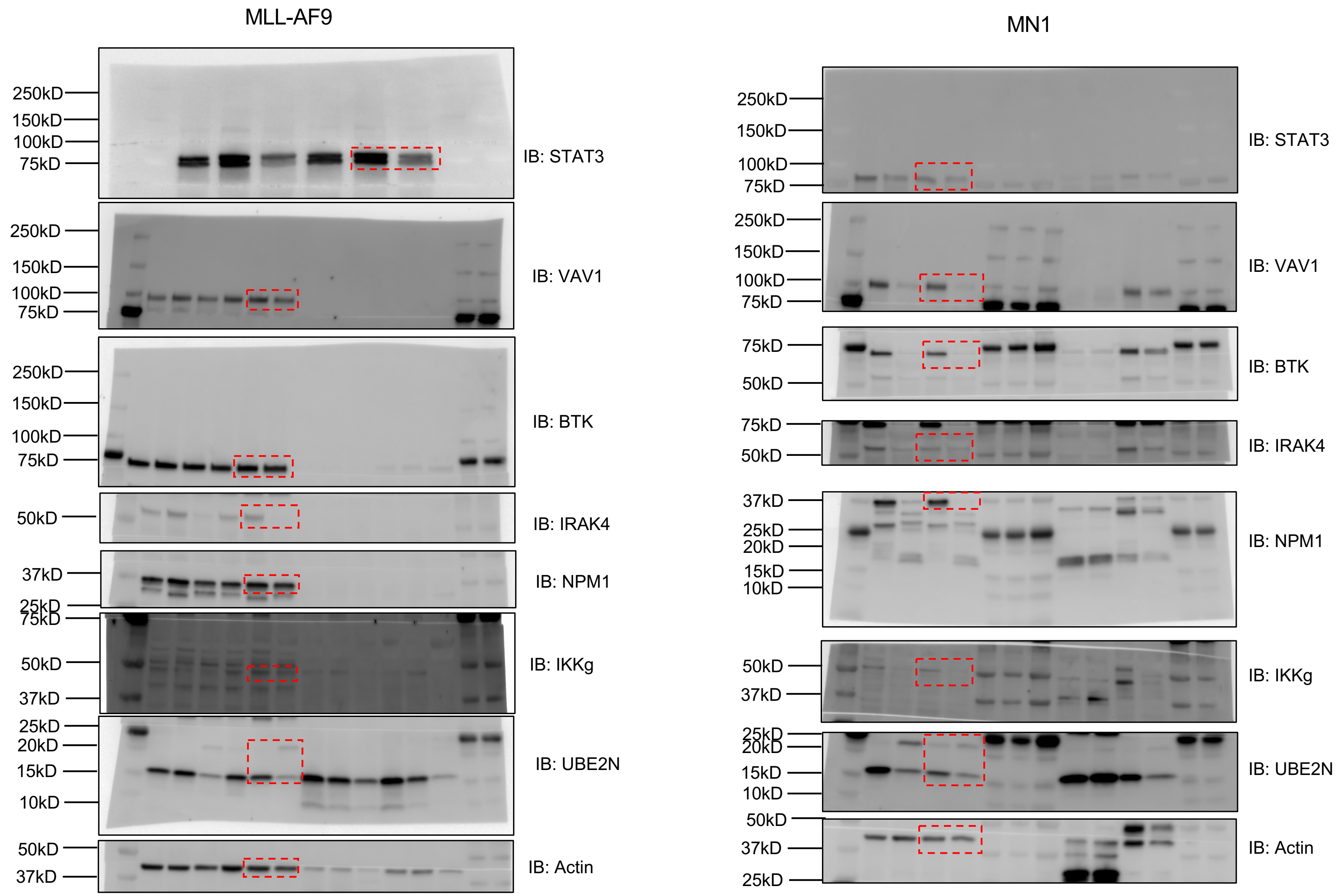
MLL-AF9
250kD
150kD
100kD
75kD
IB: STAT3
250kD
150kD
100kD
75kD
IB: VAV1
250kD
150kD
100kD
75kD
IB: BTK
50kD
IB: IRAK4
37kD
25kD
IB: NPM1
75kD
50kD
37kD
IB: IKKg
25kD
20kD
15kD
10kD
IB: UBE2N
50kD
37kD
IB: Actin
MN1
250kD
150kD
100kD
75kD
IB: STAT3
250kD
150kD
100kD
75kD
IB: VAV1
75kD
50kD
IB: BTK
75kD
50kD
IB: IRAK4
37kD
25kD
20kD
15kD
10kD
IB: NPM1
50kD
37kD
IB: IKKg
25kD
20kD
15kD
10kD
IB: UBE2N
50kD
37kD
25kD
IB: Actin

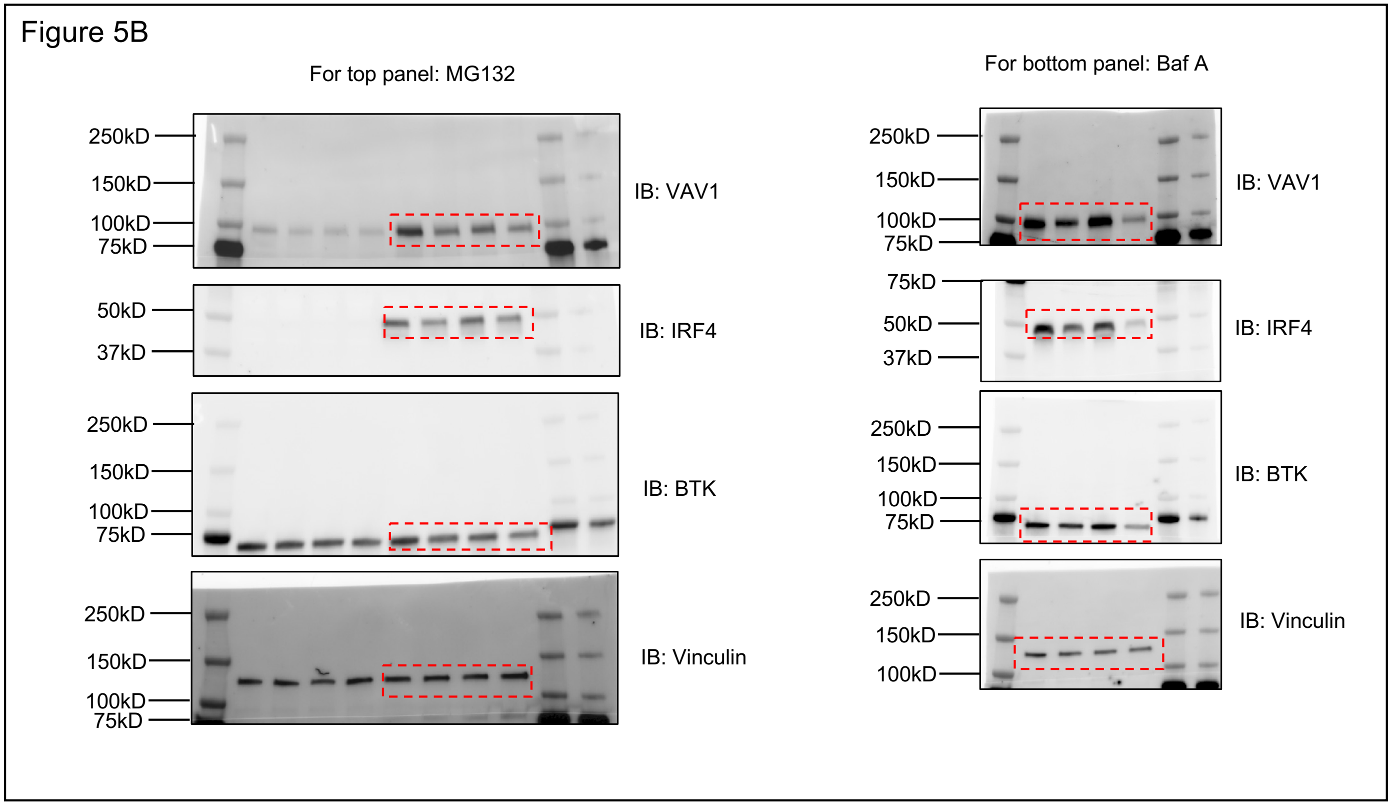
Figure 5B
For top panel: MG132
For bottom panel: Baf A
250kD
150kD
100kD
75kD
IB: VAV1
50kD
37kD
IB: IRF4
IB: BTK
IB: Vinculin

Figure 5C

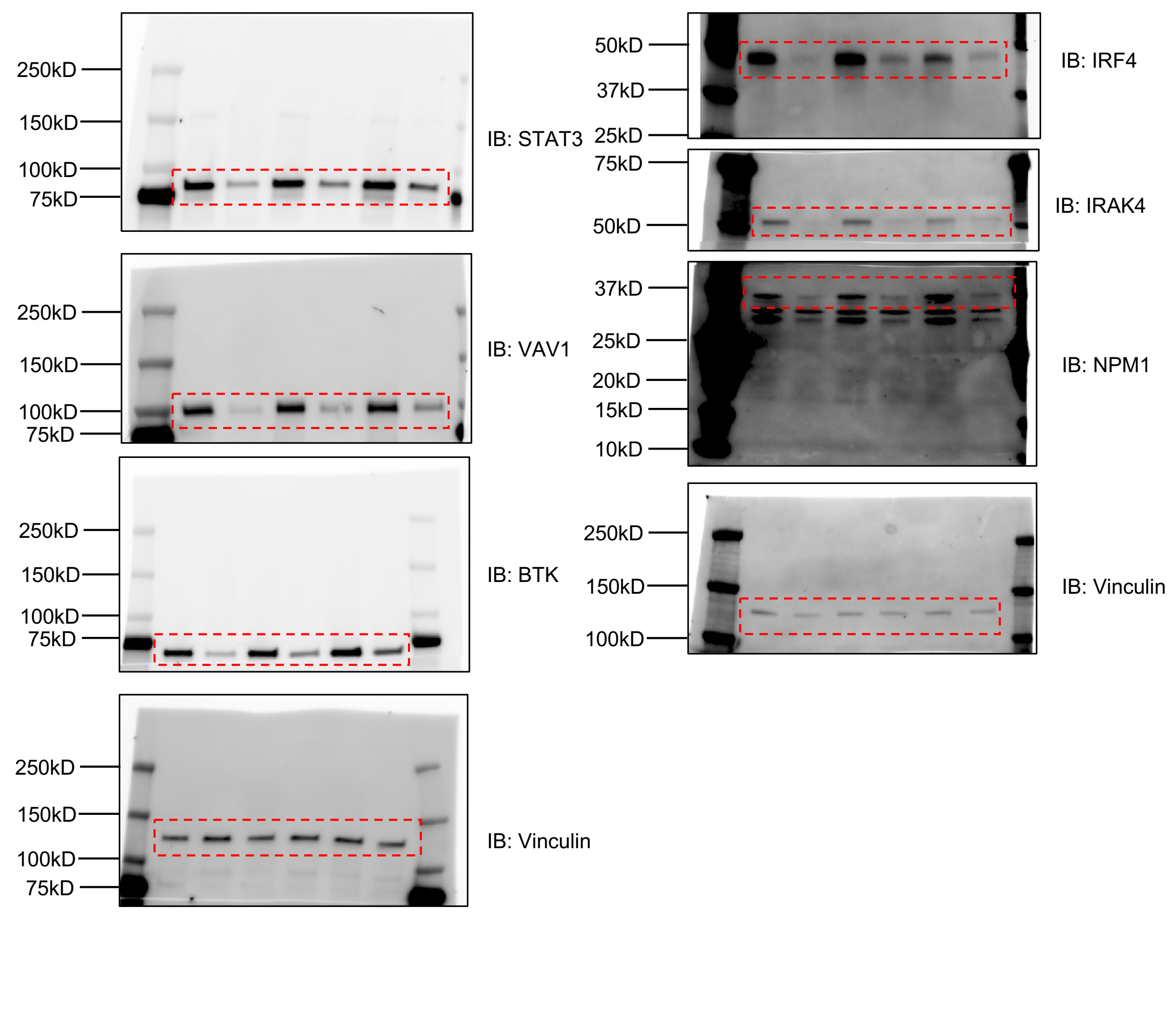

Figure 5D

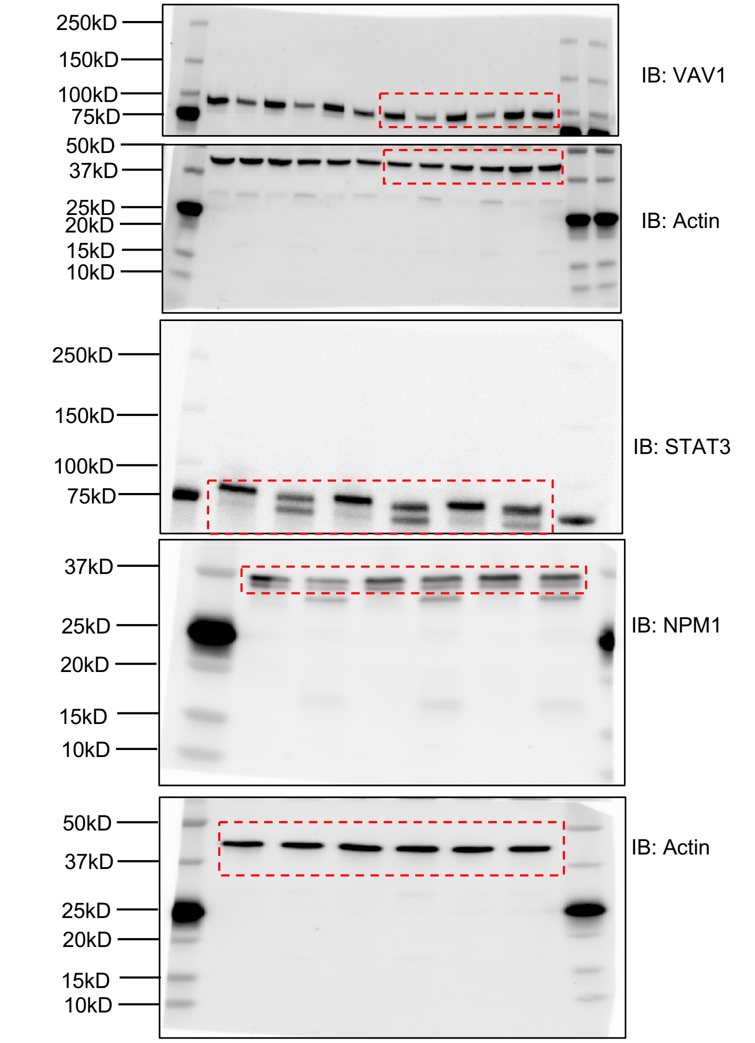

Figure 5E

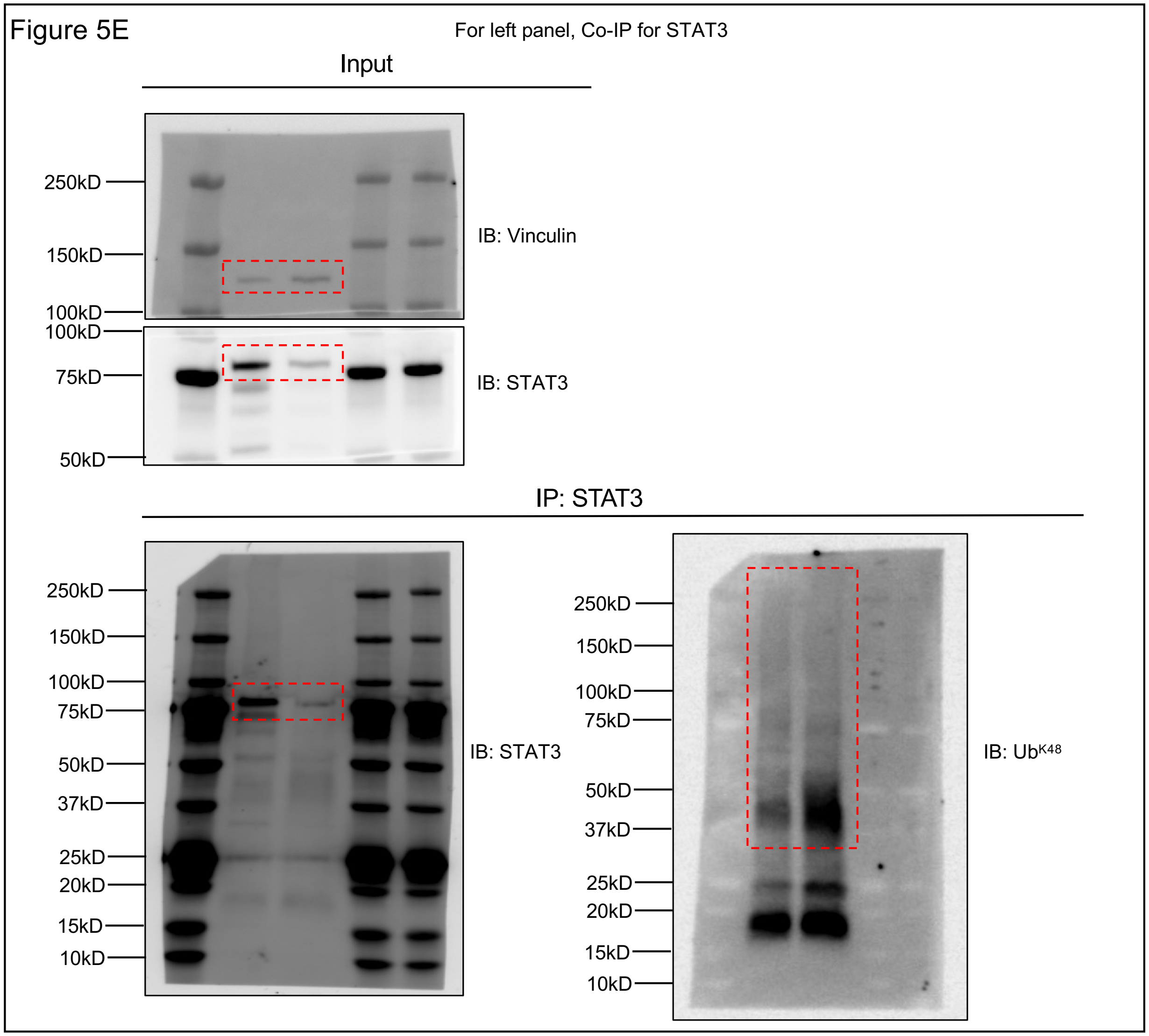
For left panel, Co-IP for STAT3
Input
250kD
150kD
100kD
100kD
75kD
50kD
IB: Vinculin
IB: STAT3
IP: STAT3
250kD
150kD
100kD
75kD
50kD
37kD
25kD
20kD
15kD
10kD
IB: STAT3
IB: Ub^K48

Figure 5E

For right panel, Co-IP for NPM1

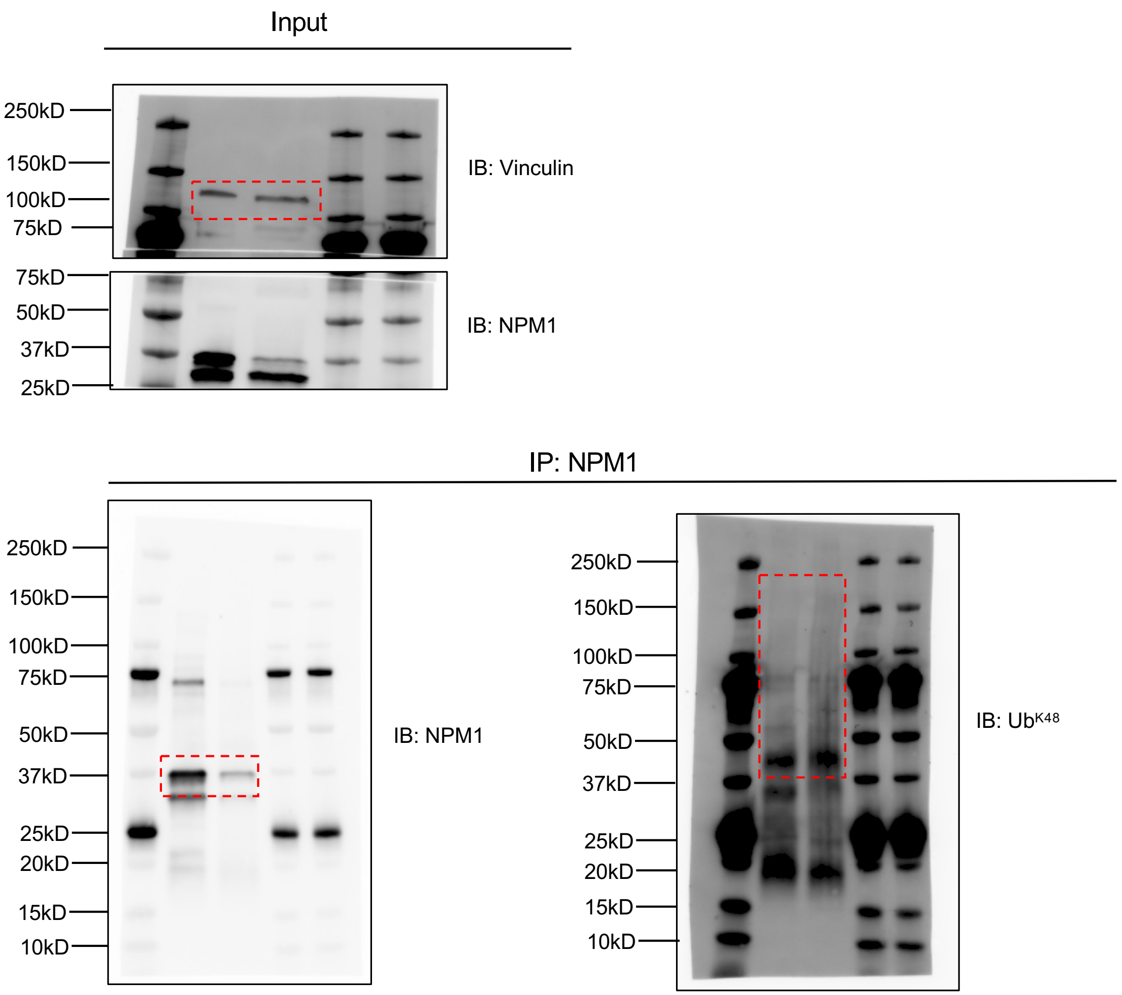

Figure 6D

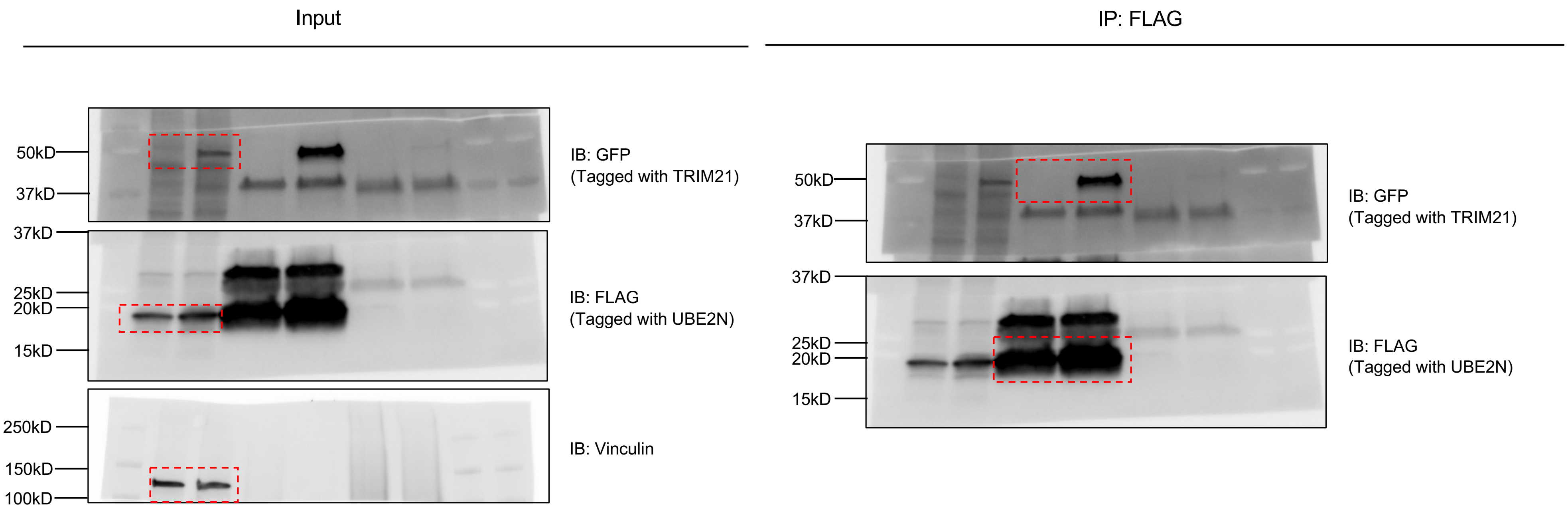
Input
IP: FLAG
50kD
37kD
37kD
25kD
20kD
15kD
250kD
150kD
100kD
IB: GFP
(Tagged with TRIM21)
IB: FLAG
(Tagged with UBE2N)
IB: Vinculin
50kD
37kD
37kD
25kD
20kD
15kD
IB: GFP
(Tagged with TRIM21)
IB: FLAG
(Tagged with UBE2N)

Figure 6E

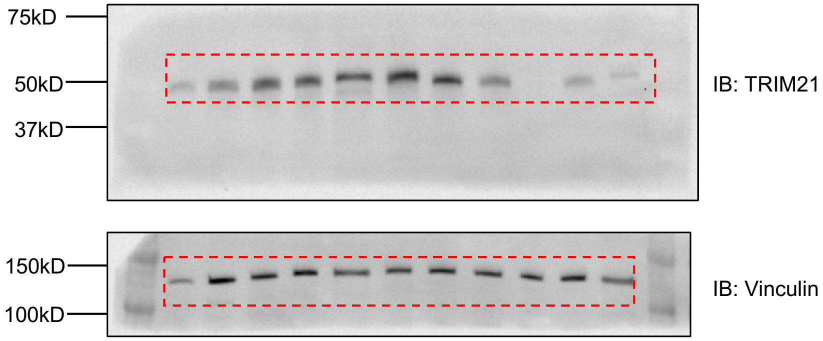

Figure 6L

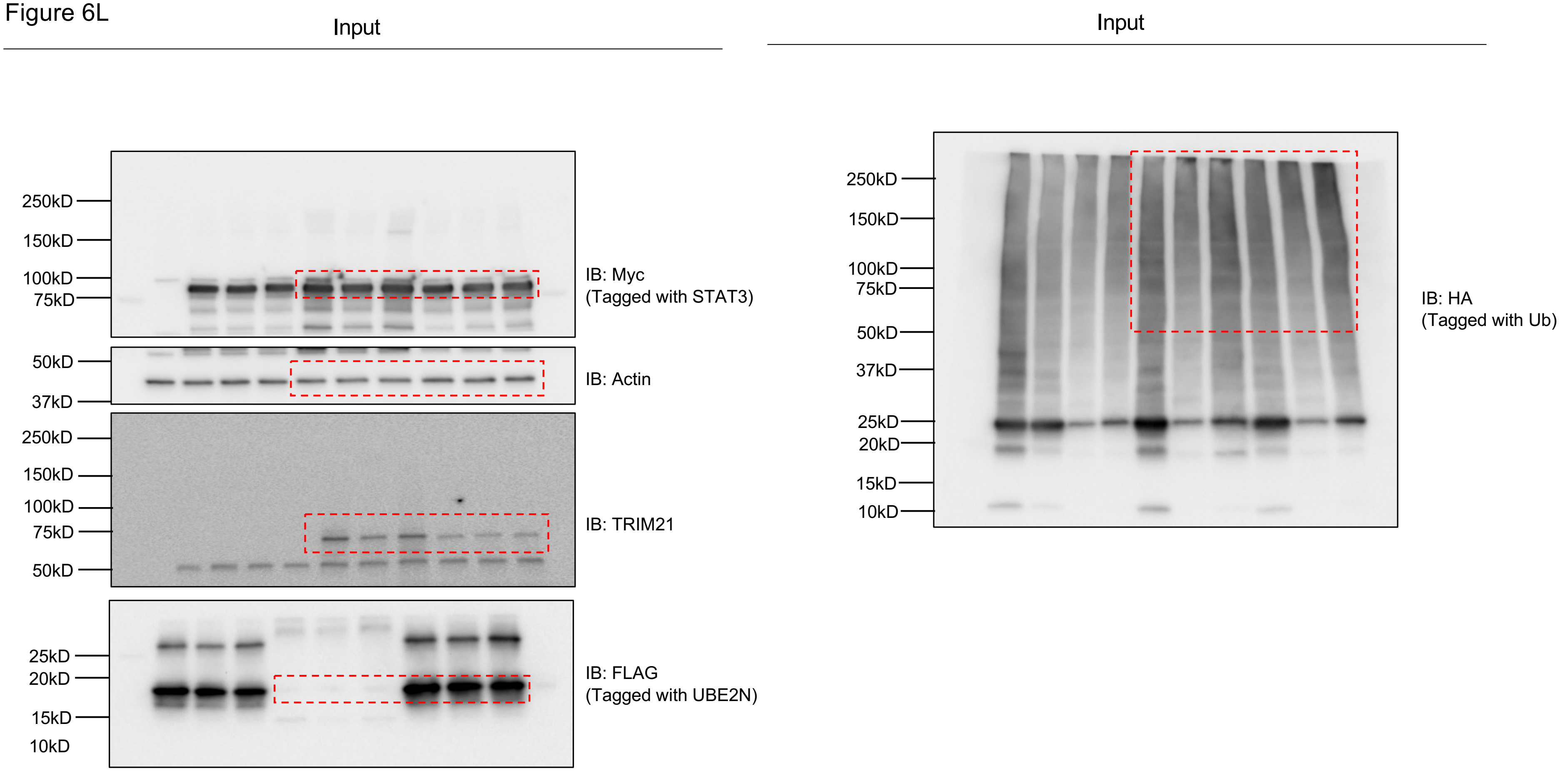
Input
250kD
150kD
100kD
75kD
IB: Myc
(Tagged with STAT3)
50kD
37kD
IB: Actin
250kD
150kD
100kD
75kD
50kD
IB: TRIM21
25kD
20kD
15kD
10kD
IB: FLAG
(Tagged with UBE2N)
Input
250kD
150kD
100kD
75kD
50kD
37kD
25kD
20kD
15kD
10kD
IB: HA
(Tagged with Ub)

Figure 6L (Continued)

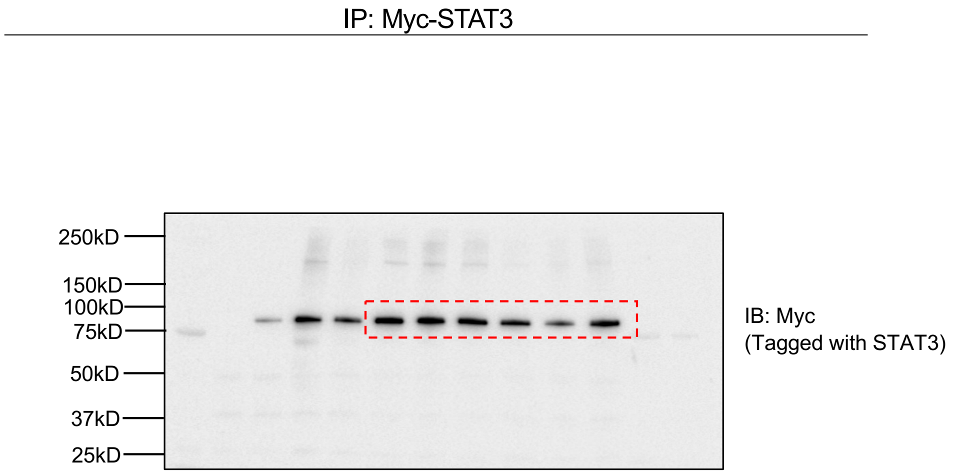

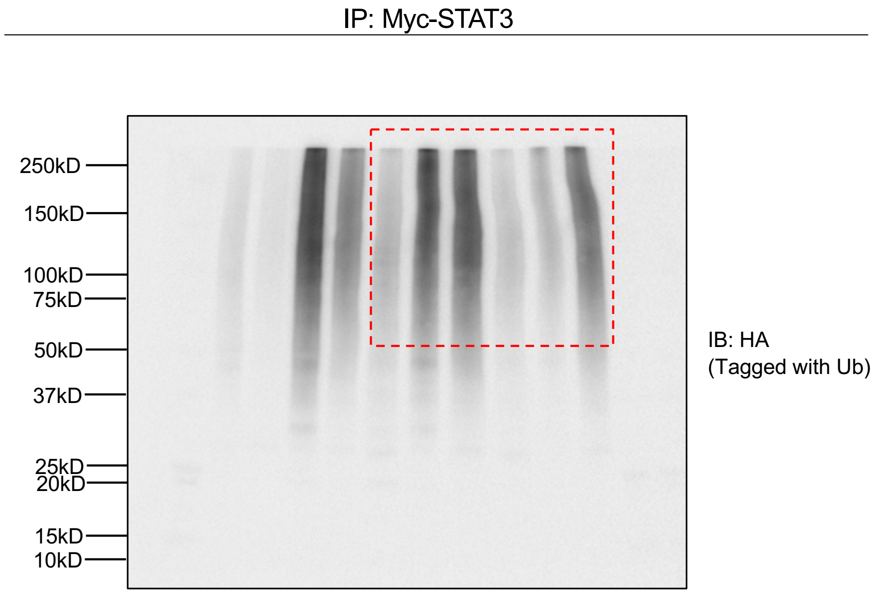

Figure 6M

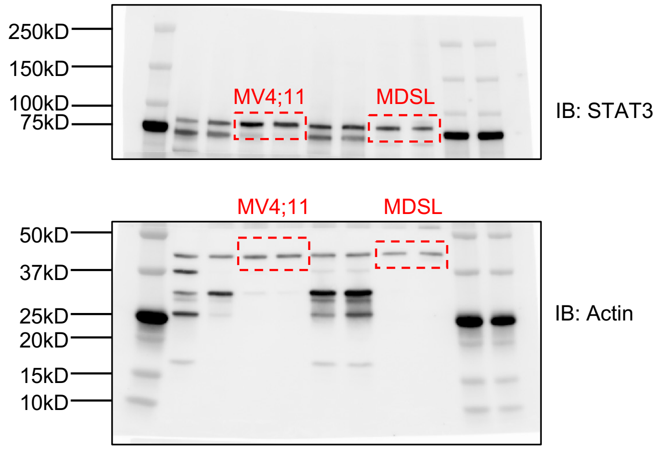

250kD
150kD
100kD
75kD
MV4;11
MDSL
IB: STAT3
MV4;11
MDSL
50kD
37kD
25kD
20kD
15kD
10kD
IB: Actin

Supplemental Figure 1E

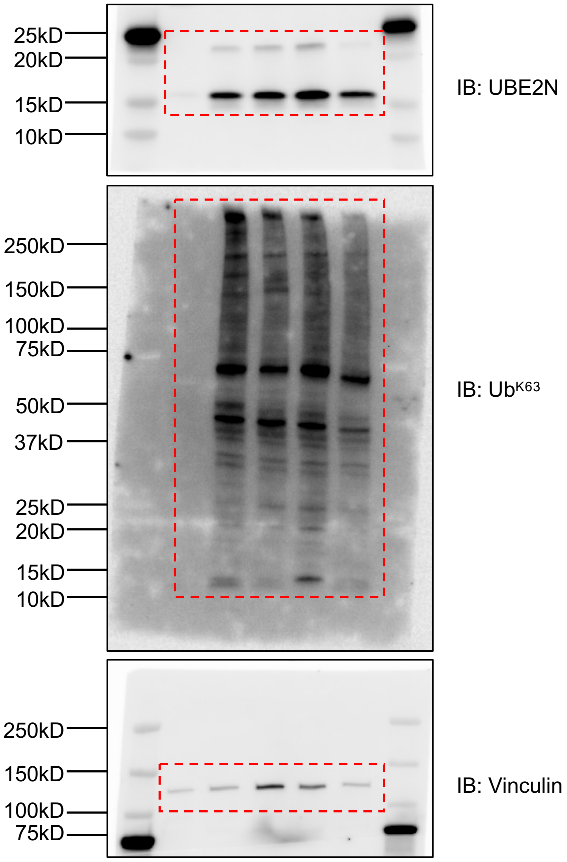

Supplemental Figure 2B

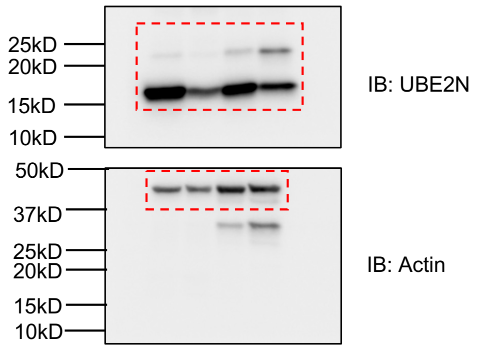

Supplemental Figure 2C

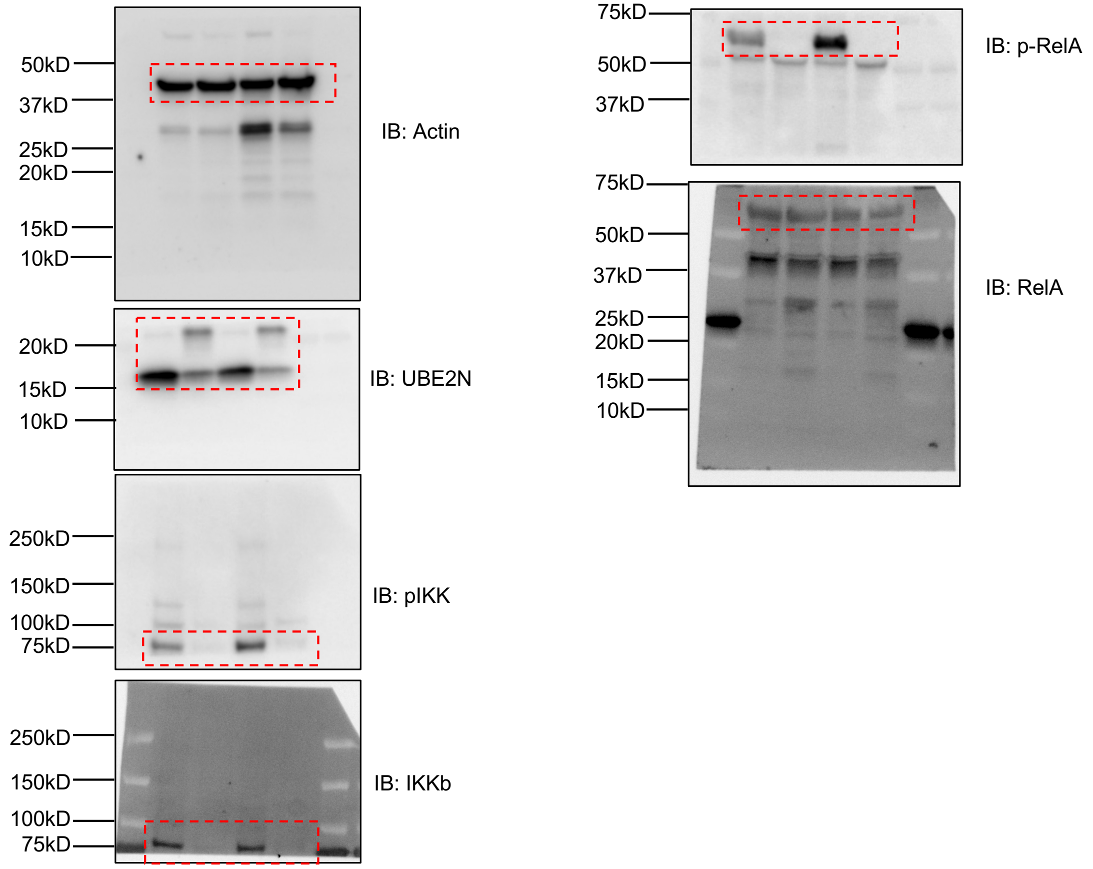

Supplemental Figure 2H

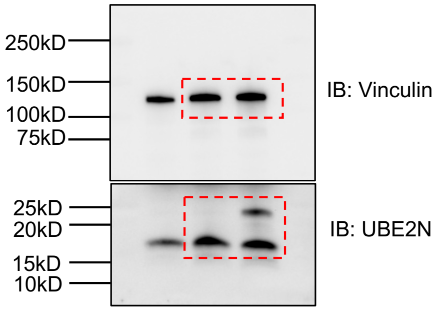

Supplemental Figure 3B

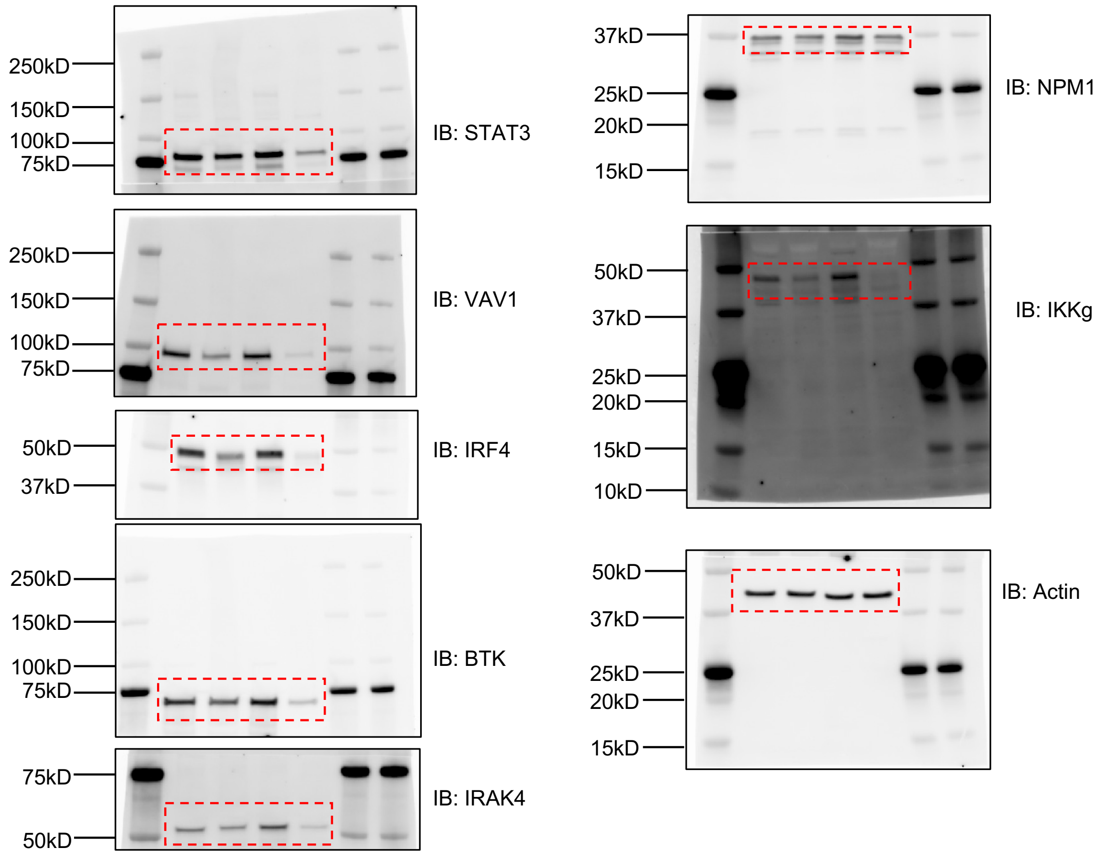

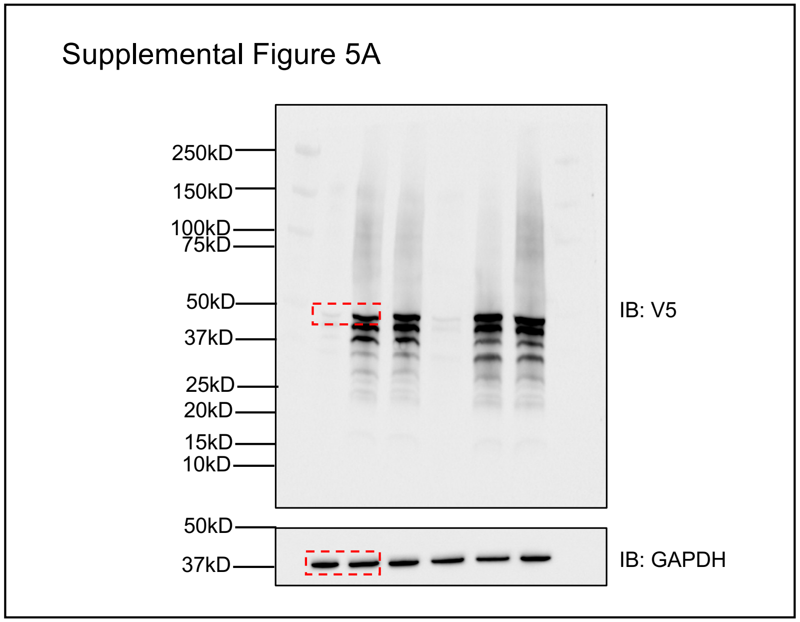
Supplemental Figure 5A
250kD
150kD
100kD
75kD
50kD
37kD
25kD
20kD
15kD
10kD
IB: V5
50kD
37kD
IB: GAPDH

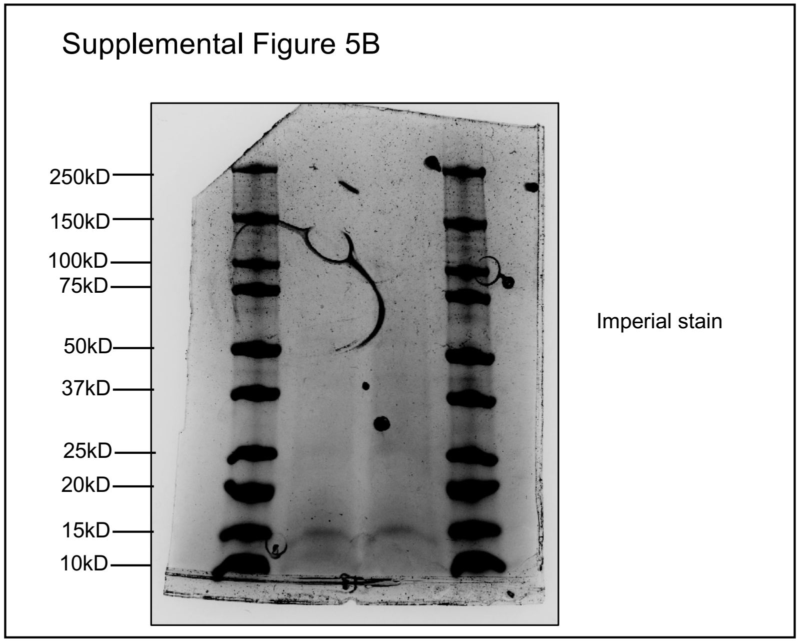
Supplemental Figure 5B
250kD
150kD
100kD
75kD
50kD
37kD
25kD
20kD
15kD
10kD
Imperial stain

Supplemental Figure 6D

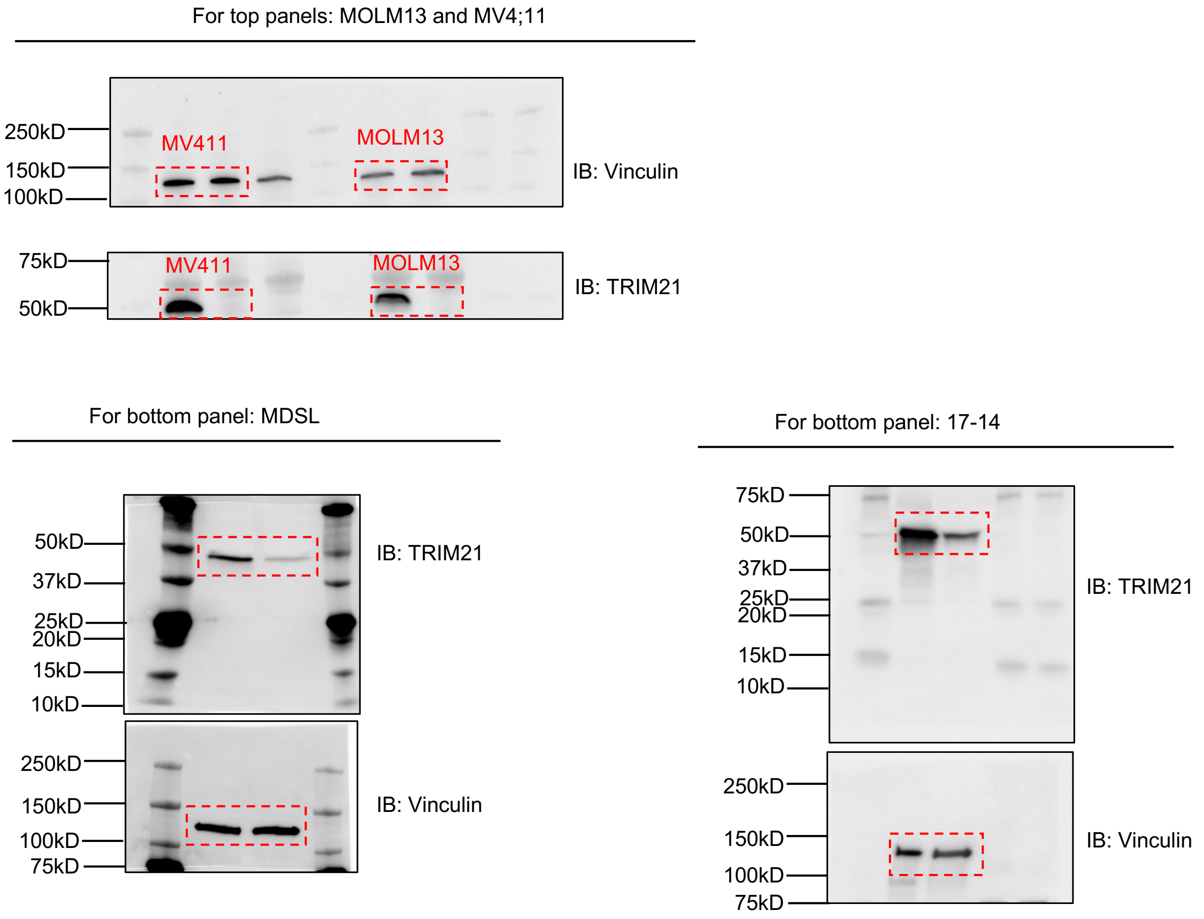

Supplemental Figure 6H

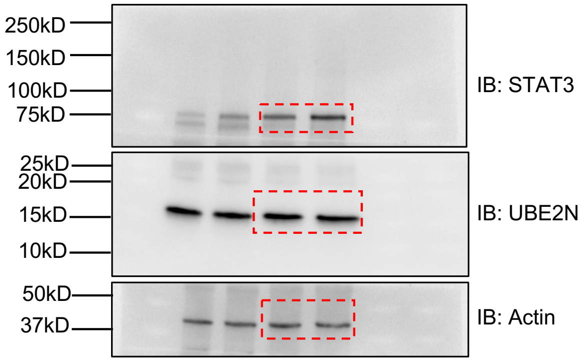

Supplemental Figure 6E

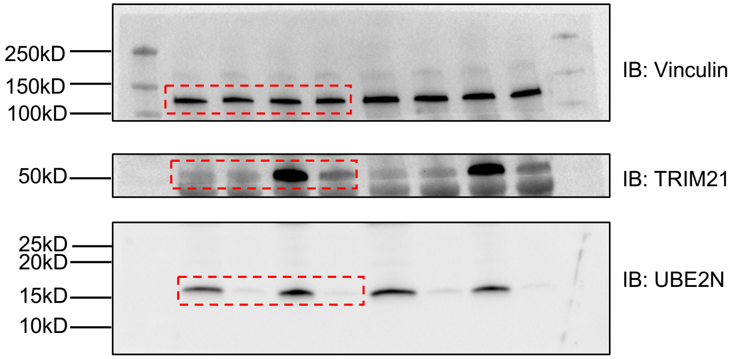

Supplement: Unedited blot and gel images [file jci-135-184665-s033.pdf]
